# Base-Promoted Chemodivergent Formation of 1,4-Benzoxazepin-5(4*H*)-ones and 1,3-Benzoxazin-4(4*H*)-ones Switched by Solvents

**DOI:** 10.3390/molecules24203773

**Published:** 2019-10-19

**Authors:** Qian Chen, Yunpeng Wang, Ruimao Hua

**Affiliations:** Department of Chemistry, Tsinghua University, Key Laboratory of Organic Optoelectronics & Molecular Engineering of Ministry of Education, Beijing 100084, China; chenqian9482@126.com (Q.C.); wangyp16@mails.tsinghua.edu.cn (Y.W.)

**Keywords:** base-promoted, chemodivergent formation, solvent-dependent transformation, 1,4-benzoxazepinones, 1,3-benzoxazinones

## Abstract

The KOH-promoted chemodivergent benzannulation of *ortho*-fluorobenzamides with 2-propyn-1-ol can afford either 1,4-benzoxazepin-5(4*H*)-ones or 1,3-benzoxazin-4(4*H*)-ones in good yields with high selectivity, depending greatly upon the use of solvents. In the case of using DMSO, the intermolecular benzannulation produced seven-membered benzo-fused heterocycles of 1,4-benzoxazepin-5(4*H*)-ones, whereas in MeCN, the six-membered benzo-fused heterocycles of 1,3-benzoxazin-4(4*H*)-ones were formed. The KOH-promoted benzannulation proceeded most probably through the C–F nucleophilic substitution of *ortho*-fluorobenzamides with 2-propyn-1-ol to give the intermediate of *ortho*-[(2-propynyl)oxy]benzamide, which underwent the intramolecular hydroamidation in a different manner to afford either seven- or six-membered benzo-fused heterocycles.

## 1. Introduction

**Scheme 1:**
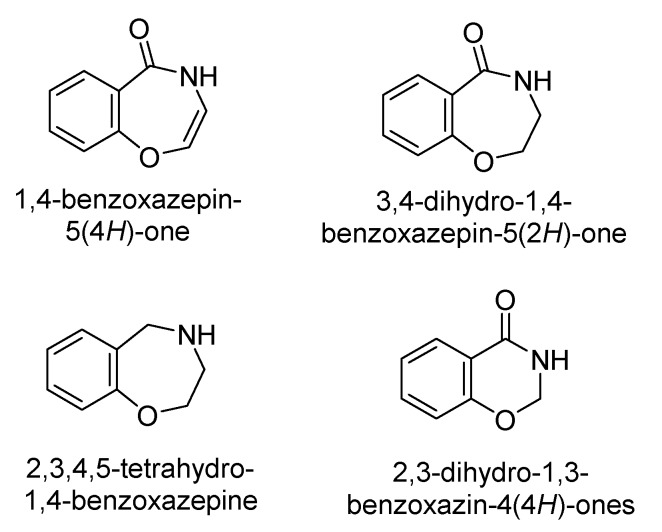
1,4-Benzoxazepin-5(4*H*)-ones, 1,3-benzoxazin-4(4*H*)-ones, and their derivatives.

Chemodivergent reactions are interesting and efficient protocols that form the structurally different heterocyclic compounds from the same starting materials through simple change of reaction conditions [1,2,3,4,5,6,7,8,9,10]. Among them, the solvent-dependent or solvent-controlled chemodivergent reactions have been well applied in the synthesis of heterocyclic compounds [11,12,13,14,15,16,17,18,19,20,21]. On the other hand, benzo-fused seven- and six-membered heterocycles containing two heteroatoms of oxygen and nitrogen such as 1,4-benzoxazepin-5(4*H*)-ones [22], 3,4-dihydro-1,4-benzoxazepin-5(2*H*)-ones [23,24], 2,3,4,5-tetrahydro-1,4-benzoxazepines [25,26,27,28,29], and 2,3-dihydro-1,3-benzoxazin-4(4*H*)-ones [30,31,32], are important and interesting heterocyclic compounds due to their wide spectrum of biological activities (Scheme 1). In addition, as the structures shown in Scheme 1, 1,4-benzoxazepin-5(4*H*)-one is the useful and potential precursor for the synthesis of its derivatives by simple transformation. Therefore, the synthetic approach to 1,4-benzoxazepin-5(4*H*)-one ring has been well investigated. However, the known procedures for the formation of the seven-membered benzo-fused heterocycle either are multi-step with low atom-utilization or using uneasily available starting materials catalyzed by palladium complexes [33,34,35].

As part of our continued interest in the development of the application of base/DMSO-promoted S_N_Ar reaction for the formation of C–N bond under transition-metal-free conditions [36,37,38], and the chemodivergent transformations of alkynes [39,40,41], as well as the new synthetic methods of heterocyclic compounds [42,43,44], we herein describe an efficient protocol for the formation of 1,4-benzoxazepin-5(4*H*)-ones and 2-vinyl-1,3-benzoxazin-4(4*H*)-ones via KOH-promoted solvent-controlled intermolecular cyclization reaction between substituted *ortho*-fluorobenzamide and 2-propyn-1-ol [45].

## 2. Results and Discussion

Table 1 concludes the results from the reaction of *ortho*-fluoro-*N*-propylbenzamide (**1a**) with 2-propyn-1-ol. We firstly examined the reaction of **1a** with 2-propyn-1-ol (1.2 equivalents) in the presence of KOH (3.0 equivalents) in DMSO at 50 °C for 12 h. Two products could be isolated from the reaction mixture, and their structures were confirmed to be *N*-propyl-3-methyl-1,4- benzoxazepin-5(4*H*)-one (**2a**, 45%) and *N*-propyl-2-vinyl-2,3-dihydro-1,3-benzoxazin-4(4*H*)-ones (**3a**, 12%) (entry 1) [46]. When the same reaction was repeated at 30 °C, the yield of **2a** was slightly increased (entry 2), and if the reaction was carried out at 30 °C for 12 h firstly and then 50 °C for 12 h, **2a** was isolated in 54% yield (entry 3). In this case, **3a** was determined only in a small amount (<5%) in the reaction mixture with the complete conversion of **1a**. The use of either 1.0 equivalent or 1.5 equivalents of 2-propyn-1-ol resulted in the decrease of **2a** yield (entries 4 and 5). Increase of the reaction temperature to 70 °C for 12 h also led to the lower yield of **2a** (entry 6). Decreasing the amount of KOH to 1.0 or 2.0 equivalents affected the formation of **2a** (entries 7 and 8). Other inorganic bases, such as NaOH, K_2_CO_3_, Cs_2_CO_3_, and *t*-BuOK were not efficient in promoting the cyclocondensation (entries 9–12). Very interestingly, the screening of solvents disclosed that the selective chemodivergent formation of **2a** and **3a** greatly depended on the use of solvents (entries 13–18). In the cases of THF, DMF and a mixture solvent of DMSO/MeCN used, **3a** was the major product, and in MeCN, **3a** could be isolated in 83% yield with a small amount of **2a** formation. In addition, either increasing the amount of 2-propyn-1-ol or increasing the reaction temperature did not improve the yield of **3a** (entries 19 and 20).

**Scheme 2:**
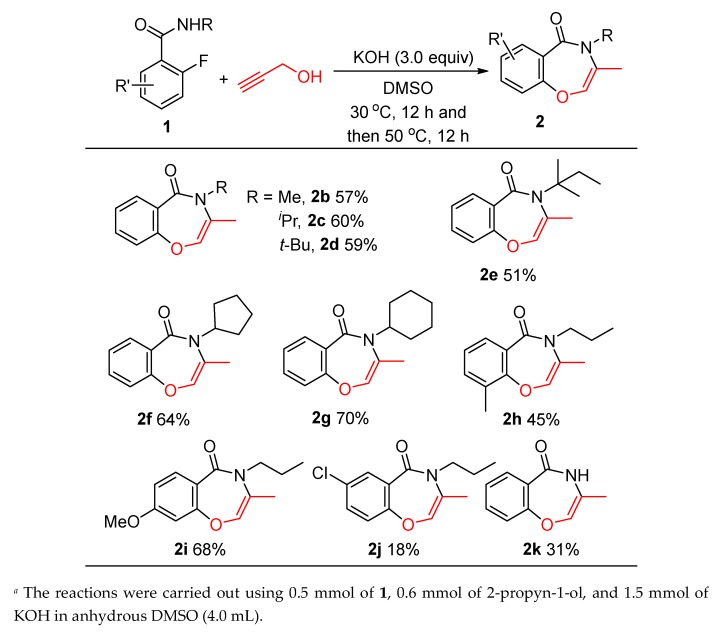
Formation of 1,4-benzoxazepin-5(4*H*)-ones in KOH/DMSO *^a^.*

The cyclocondensation of other substrates **1b**–**k**, bearing different substituents on nitrogen or benzene ring with 2-propyn-1-ol, was then studied under the conditions of entry 3 in Table 1. As can be seen from Scheme 2, *N*-alkyl-2-fluorobenzamides (alkyl = Me (**1b**), isopropyl (**1c**), *t*-butyl (**1d**), *t*-amyl (**1e**), cyclopentyl (**1f**), cyclohexyl (**1g**)) showed similar reactivity to **1a** affording the corresponding *N*-alkyl-3-methyl-1,4-benzoxazepin-5(4*H*)-ones (**2b**–**2g**) in good yields. It was also noted that *N*-phenyl-2-fluorobenzamide showed unexpectedly sluggish reactivity, and almost all starting materials could be recovered after the reaction under the same conditions. In addition, the results from the reactions of 2-fluoro-3-methyl-*N*-propylbenzamide (**1h**), 2-fluoro-4-methoxy-*N*- propylbenzamide (**1i**), and 4-chloro-2-fluoro-*N*-propylbenzamide (**1j**) with 2-propyn-1-ol apparently indicated that the electron-withdrawing group in **1j** was unfavorable in the formation of 1,4-benzoxazepin-5(4*H*)-one ring. Moreover, the reaction of 2-fluorobenzamide (**1k**), bearing an unprotected NH_2_ group, afforded the corresponding product (**2k**) in only 31% yield. It should be noted that the reaction of *ortho*-fluorobenzamides bearing a strong electron-withdrawing group, such as 2-fluoro-5-nitro-*N*-propylbenzamide (**1l**) and 2-fluoro-*N*-propyl-5-trifluoromethyl- benzamide (**1m**), resulted in a complex mixture.

**Scheme 3:**
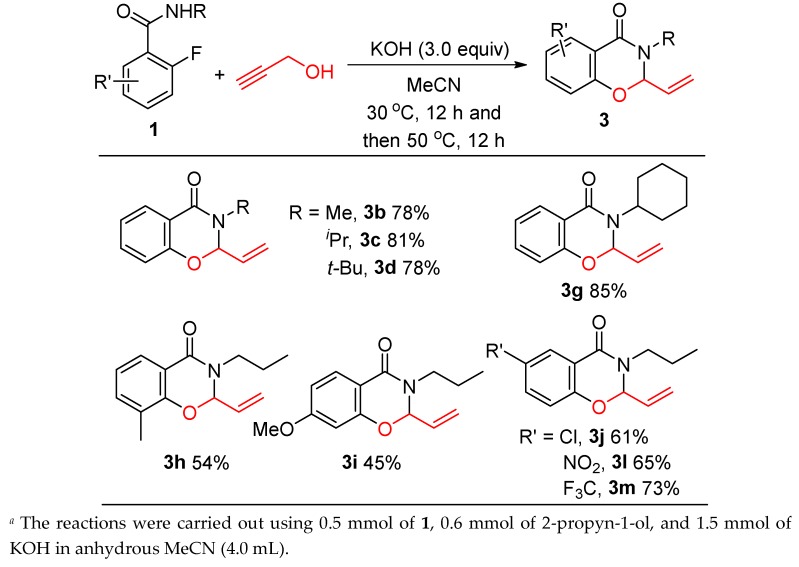
Formation of 1,3-benzoxazin-4(4*H*)-ones in KOH/MeCN *^a^.*

We also examined the substrate scope under the reaction conditions indicated in entry 17 of Table 1 access to 1,3-benzoxazin-4(4*H*)-ones (**3**) by reacting 2-propyn-1-ol with different *ortho*-fluorobenzamides in MeCN. As summarized in Scheme 3, the reactions of **1b**–**d** and **1g**–**j** afforded the corresponding 2-vinyl-1,3-benzoxazin-4(4*H*)-one derivatives **3b**–**d** and **3g**–**j** in good to high yields. It should be noted that, compared to KOH/DMSO system, *ortho*-fluorobenzamides having electron-withdrawing group displayed a higher reactivity than ones with an electron-donating group (**1j** vs. **1i**) in KOH/MeCN in undergoing the cyclization reaction to give the expected cyclic products in good yield (**3j** vs. **2j** and **3i** vs. **2i**) (vide infra). In addition, both **1l** and **1m** also showed good reactivity, giving the corresponding products of **3l** and **3m** (vide supra). Note that in KOH/MeCN, all the reactions occurred with excellent chemoselectivity, and only a trace amount of the corresponding product **2** formed in the reaction mixtures.

However, when substituted propargyl alcohols, such as 1-methyl-2-propyn-1-ol, 1-phenyl- 2-propyn-1-ol, 3-methyl-2-propyn-1-ol, and 3-phenyl-2-propyn-1-ol, were subjected to the similar reaction conditions, although the formation of the corresponding cyclic compounds **2** and **3** could be determined by GC-MS, the reactions unfortunately occurred not only with low chemoselectivity in both DMSO and MeCN solvents, but also with low total yields of **2** and **3**.

**Scheme 4:**
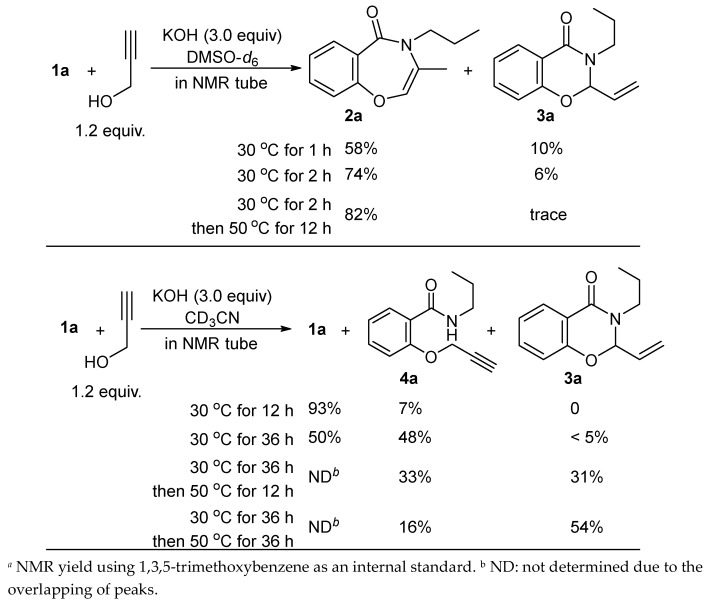
Real-time monitoring of **1a** with 2-propyn-1-ol reaction ^a.^

In order to understand the chemodivergent formation of **2** and **3** switched by solvents, the observation of real-time reactions of **1a** with 2-propyn-1-ol in NMR tube using DMSO-*d*_6_ or CD_3_CN were introduced. As shown in Scheme 4, in KOH/DMSO-*d*_6_, the cyclization reaction occurred fast, and a 1 h reaction at 30 °C resulted in the formation of **2a** and **3a** in 58% and 10% NMR yields, respectively. In this case, no considerable amount of intermediates could be observed in ^1^H-NMR. An additional 1 h reaction led to the yield increase of **2a** to 74%, whereas the NMR yield of **3a** was decreased to 6%. A subsequent 2 h reaction at 50 °C afforded **2a** in 82% NMR yield, and **3a** was determined in a small amount (<5%). In addition, we also examined the conversion of **3a** in KOH/DMSO at 50 °C for 2 h, and found that **3a** completely disappeared due possibly to its polymerization, as the formation of **2a** could not be observed at all in the reaction mixture. Therefore, it can be concluded that in the KOH/DMSO system, the excellent regioselectivity for the formation of **2a** resulted from the easy formation of **2a** and the side-reaction of **3a**.

On the other hand, in KOH/CD_3_CN, the reaction of **1a** with 2-propyn-1-ol in NMR tube without stirring occurred very slowly, and only small amount of intermediate **4a** could be determined from the S_N_Ar reaction. **1a** was almost remained, and neither **2a** nor **3a** formed at all at 30 °C for 12 h. Even a prolonged reaction time (at 30 °C for 36 h) did not result in the formation of considerable amount of **3a**, and in this case, **4a** was the major product. With the subsequent reaction at 50 °C for 12 h, **3a** formed in 31% NMR yield, and in the sequent reaction for 36 h, the yield of **3a** was increased to 54%.

Taking into consideration of the results shown in Scheme 2, Scheme 3 and Scheme 4, it might be concluded that the chemodivergent formation of either **2** or **3** switched by using DMSO or MeCN as solvents resulted from the base strength of the reaction mixture. KOH/DMSO was well-applied as the superbase medium to promote the diverse organic transformation due to the high solubility of KOH in DMSO [47], whereas KOH/MeCN is a medium alkaline condition owing to the low solubility of KOH in MeCN. In fact, the present reaction mixtures in DMSO were homogeneous to afford **2**, but the reaction mixtures in MeCN were heterogeneous to give **3**. Thus, it is also easy to understand why in NMR tube, without stirring, a very low rate of reaction in CD_3_CN was observed (Scheme 4 vs. Scheme 3).

**Scheme 5:**
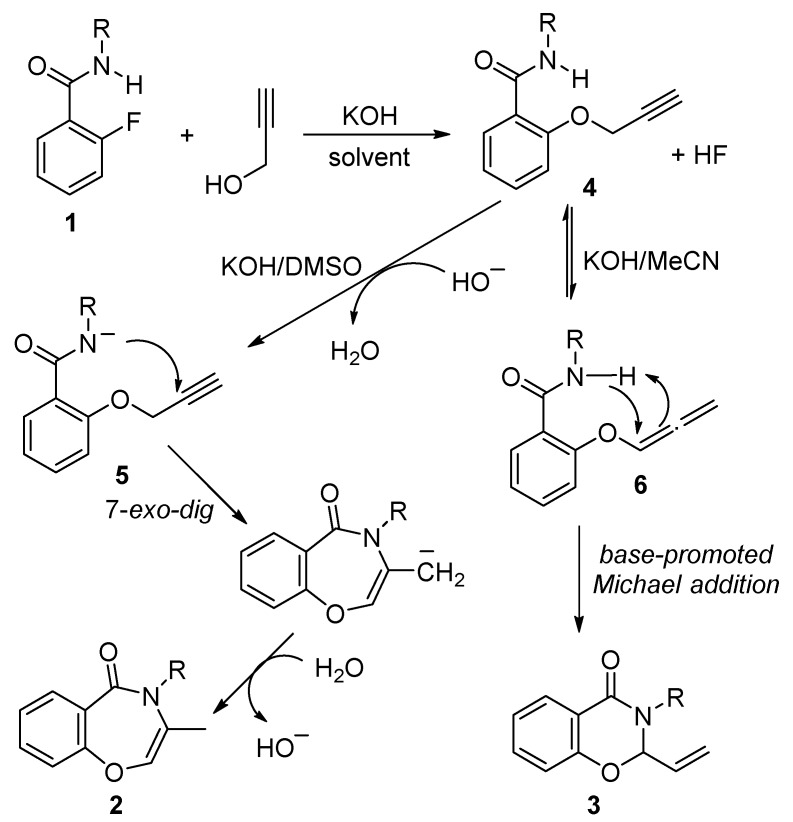
Proposed mechanism for the chemodivergent formation of 1,4-benzoxazepin-5(4*H*)-ones and 1,3-benzoxazin-4(4*H*)-ones.

On the basis of the obtained results and above discussion, a proposed mechanism for the formation of either **2** in DMSO or **3** in MeCN is depicted in Scheme 5. It involves the S_N_Ar reaction of **1** with 2-propyn-1-ol, giving the intermediate of *ortho*-[(2-propynyl)oxy]benzamide **4**. In the KOH/DMSO system, a superbase medium [47], the formation of **4** and the subsequent formation of a nitrogen anion **5** are very fast and favorable, leading to the quickly intramolecular nucleophilic addition to alkyne concurrently to construct the seven-membered ring via a *7-exo-dig* cyclization, and the final protonation step afforded **2**. On the other hand, in the KOH/MeCN system, a relatively weak base medium compared to KOH/DMSO, not only is the formation of **4** slow, but also the isomerization of **4** into allenyl intermediate **6** is possible [48], which undergoes the base-promoted intramolecular Michael addition of N–H to allene giving **3** [49].

**Scheme 6:**
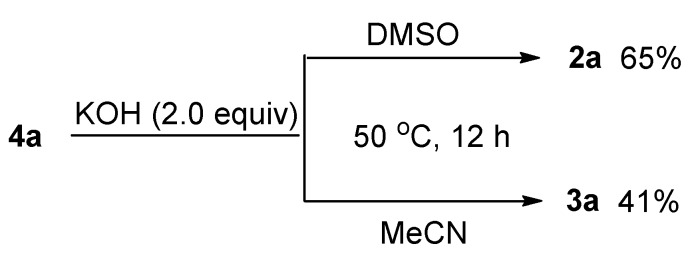
Formation of **2a** and **3a** from intermediate **4a**.

We also examined the conversion of the isolated intermediate **4a** under the similar reaction conditions as shown in Scheme 2 and Scheme 3. As expected, **2a** and **3a** could be isolated in 65% and 41% yields, respectively (Scheme 6).

## 3. Materials and Methods 

### 3.1. General Methods

All commercial reagents and metal salts are analytically pure and used directly without further purification. **1k** is commercially available and other *ortho*-fluoro-*N*-alkylbenzamides are known compounds and were prepared by a modified procedure via the reactions of *ortho*-fluorobenzoyl chlorides with primary amines (the procedure, yields in both weight and percentage, and ^1^H-NMR charts are reported in the Supplementary Information) [50]. KOH (99.99%) was obtained from Sigma-Aldrich (St. Louis, MO, USA). Nuclear magnetic resonance (NMR) spectra were recorded on an ECA-400 spectrometer (JEOL, Tokyo, Japan) using CDCl_3_ as solvent at 298 K. ^1^H NMR (400 MHz) chemical shifts (δ) were referenced to internal standard TMS (for ^1^H, δ = 0.00). ^13^C NMR (100 MHz) chemical shifts were referenced to internal solvent CDCl_3_ (for ^13^C, δ = 77.16). The high-resolution mass spectra (HRMS) with electron spray ionization (ESI) were obtained with a micrOTOF-Q spectrometer (Agilent, Santa Clara, CA, USA). The melting points were uncorrected. Single crystals of **2g** and **3g** were obtained by slow evaporation of their solution in a mixture solvent of acetone and *n*-hexane.

### 3.2. Typical Experimental Procedure for the Synthesis of N-Propyl-3-methyl-1,4-benzoxazepin-5(4H)-one *(**2a**)*

A mixture of 2-fluoro-*N*-propylbenzamide (**1a**, 90.5 mg, 0.5 mmol), 2-propyl-1-ol (*ca*. 34.0 mg, 0.6 mmol), KOH (84.0 mg, 1.5 mmol), and DMSO (4.0 mL) in a 25 mL screw-capped thick-walled Pyrex tube was stirred at 30 °C for 12 h, and then at 50 °C for 12 h. After the reaction mixture was cooled to room temperature, water (10 mL) was added with stirring, and the mixture was extracted with ethyl acetate three times (3 × 10 mL). The combined organic phases were dried over anhydrous MgSO_4_. The filtered solution was then concentrated under reduced pressure, and the crude residue was purified by column chromatography on silica gel with the use of petroleum ether/ethyl acetate (gradient mixture ratio from 20:1 to 4:1 in volume) to afford **2a** as a pale yellow oil in 54% yield (58.5 mg).

When MeCN was used as solvent to replace DMSO, the similar operation afforded *N*-propyl-2-vinyl-2,3-dihydro-1,3-benzoxazin-4(4*H*)-ones (**3a**) as a pale yellow oil in 83% yield (90.5 mg).

### 3.3. Characterization Data of Products:

*N-Propyl-3-methyl-1,4-benzoxazepin-5(4H)-one* (**2a**): Pale yellow oil (58.5 mg, 54%). ^1^H NMR (400 MHz, CDCl_3_): δ 7.86 (dd, *J* = 7.8, 1.6 Hz, 1H), 7.40 (apparent td, *J* = 7.8, 1.6 Hz, 1H), 7.18 (apparent t, *J* = 7.8 Hz, 1H), 6.96 (d, *J* = 7.8 Hz, 1H), 5.43 (s, 1H), 3.57 (t, *J* = 7.4 Hz, 2H), 1.93 (s, 3H), 1.64–1.73 (m, 2H), 0.96 (t, *J* = 7.4 Hz, 3H). ^13^C NMR (100 MHz, CDCl_3_): δ 166.6, 160.5, 148.5, 133.1, 132.2, 127.3, 124.8, 120.1, 115.0, 49.8, 21.4, 17.7, 11.2. HRMS (ESI) *m/z*: [M + H]^+^ calcd for C_13_H_16_NO_2_, 218.1176; found, 218.1174.

*N-Methyl-3-methyl-1,4-benzoxazepin-5(4H)-one* (**2b**): Pale yellow oil (54.1 mg, 57%). ^1^H NMR (400 MHz, CDCl_3_) δ 7.87 (dd, *J* = 7.8, 1.8 Hz, 1H), 7.41 (apparent td, *J* = 7.8, 1.8 Hz, 1H), 7.19 (apparent td, *J* = 7.8, 1.8 Hz, 1H), 6.97 (dd, *J* = 7.8, 1.8 Hz, 1H), 5.46 (s, 1H), 3.19 (s, 3H), 1.93 (s, 3H). ^13^C NMR (100 MHz, CDCl_3_) δ 166.8, 160.4, 147.9, 133.1, 132.1, 126.9, 124.8, 120.1, 115.8, 35.9, 17.5. HRMS (ESI) *m/z*: [M + H]^+^ calcd for C_11_H_12_NO_2_, 190.0863; found, 190.0862.

*N-Isopropyl-3-methyl-1,4-benzoxazepin-5(4H)-one* (**2c**): Pale yellow oil (64.7 mg, 60%). ^1^H NMR (400 MHz, CDCl_3_): δ 7.88 (dd, *J* = 7.8, 1.6 Hz, 1H), 7.39 (apparent td, *J* = 7.8, 1.6 Hz, 1H), 7.18 (apparent td, *J* = 7.8, 1.6 Hz, 1H), 6.96 (dd, *J* = 7.8, 1.6 Hz, 1H), 5.49 (s, 1H), 5.04 (hept, *J* = 6.8 Hz, 1H), 1.95 (s, 3H), 1.23 (d, *J* = 6.8 Hz, 6H). ^13^C NMR (100 MHz, CDCl_3_): δ 166.2, 160.5, 149.8, 132.9, 132.4, 127.3, 124.7, 112.0, 109.9, 45.7, 20.3, 17.7. HRMS (ESI) *m/z*: [M + H]^+^ calcd for C_13_H_16_NO_2_, 218.1176; found, 218.1173.

*N-t-Butyl-3-methyl-1,4-benzoxazepin-5(4H)-one* (**2d**): Pale yellow oil (68.2 mg, 59%). ^1^H NMR (400 MHz, CDCl_3_): δ 7.92 (dd, *J* = 7.8, 1.6 Hz, 1H), 7.37 (apparent td, *J* = 7.8, 1.6 Hz, 1H), 7.17 (apparent td, *J* = 7.8, 1.6 Hz, 1H), 6.96 (dd, *J* = 7.8, 1.6 Hz, 1H), 5.59 (q, *J* = 0.8 Hz, 1H), 1.90 (d, *J* = 0.8 Hz, 3H), 1.54 (s, 9H). ^13^C NMR (100 MHz, CDCl_3_): δ 166.7, 161.2, 150.0, 132.5, 128.2, 124.5, 119.7, 113.7, 58.9, 28.7, 17.2. HRMS (ESI) *m/z*: [M + H]^+^ calcd for C_14_H_18_NO_2_, 232.1332; found, 232.1332.

*N-t-Amyl-3-methyl-1,4-benzoxazepin-5(4H)-one* (**2e**): Pale yellow oil (62.1 mg, 51%). ^1^H NMR (400 MHz, CDCl_3_): δ 7.90 (dd, *J* = 7.8, 1.6 Hz, 1H), 7.36 (apparent td, *J* = 7.8, 1.6 Hz, 1H), 7.16 (apparent td, *J* = 7.8, 1.6 Hz, 1H), 6.96 (dd, *J* = 7.8, 1.6 Hz, 1H), 5.56 (s, 1H), 2.02 (q, *J* = 7.5 Hz, 2H), 1.89 (s, 3H), 1.48 (s, 6H), 0.89 (t, *J* = 7.5 Hz, 3H). ^13^C NMR (100 MHz, CDCl_3_): δ 166.8, 161.3, 150.2, 132.5, 128.3, 124.6, 119.7, 114.3, 62.0, 32.4, 27.0, 17.2, 8.6. HRMS (ESI) *m/z*: [M + H]^+^ calcd for C_15_H_20_NO_2_, 246.1489; found, 246.1487.

*N-Cyclopentyl-3-methyl-1,4-benzoxazepin-5(4H)-one* (**2f**): Pale yellow oil (78.1 mg, 64%). ^1^H NMR (400 MHz, CDCl_3_): δ 7.86 (dd, *J* = 7.8, 1.6 Hz, 1H), 7.37 (apparent td, *J* = 7.8, 1.6 Hz, 1H), 7.16 (apparent td, *J* = 7.8, 1.6 Hz, 1H), 6.94 (dd, *J* = 7.8, 1.6 Hz, 1H), 5.44 (s, 1H), 5.10 (pent, *J* = 8.2 Hz, 1H), 2.00–1.95 (m, 2H), 1.93 (s, 3H), 1.74–1.53 (m, 6H). ^13^C NMR (100 MHz, CDCl_3_): δ 166.7, 160.5, 149.8, 132.9, 132.5, 127.3, 124.7, 112.0, 110.8, 55.6, 29.6, 24.6, 17.8. HRMS (ESI) *m/z*: [M + H]^+^ calcd for C_15_H_18_NO_2_, 244.1332; found, 244.1331.

*N-Cyclohexyl-3-methyl-1,4-benzoxazepin-5(4H)-one* (**2g**): Pale yellow solid (89.4 mg, 70%). mp 63–65 °C. ^1^H NMR (400 MHz, CDCl_3_): δ 7.87 (dd, *J* = 7.8, 1.6 Hz, 1H), 7.40 (apparent td, *J* = 7.8, 1.6 Hz, 1H), 7.19 (apparent td, *J* = 7.8, 1.6 Hz, 1H), 6.96 (dd, *J* = 7.8, 1.6 Hz, 1H), 5.51 (q, *J* = 0.9 Hz, 1H), 4.67–4.55 (m, 1H), 1.94 (d, *J* = 0.9 Hz, 3H), 1.91–1.78 (m, 4H), 1.73–1.62 (m, 2H), 1.50–1.40 (m, 4H). ^13^C NMR (100 MHz, CDCl_3_): δ 166.2, 160.5, 149.3, 132.8, 132.4, 127.4, 124.7, 119.9, 110.9, 53.9, 30.7, 25.8, 25.6, 17.7. HRMS (ESI) *m/z*: [M + H]^+^ calcd for C_16_H_20_NO_2_, 258.1489; found, 258.1488.

*N-Propyl-3,9-dimethyl-1,4-benzoxazepin-5(4H)-one* (**2h**): Pale yellow oil (52.3 mg, 45%). ^1^H NMR (400 MHz, CDCl_3_): δ 7.67 (dd, *J* = 7.8, 1.5 Hz, 1H), 7.27 (d, *J* = 7.8 Hz, 1H), 7.07 (apparent t, *J* = 7.8 Hz, 1H), 5.45 (s, 1H), 3.57 (t, *J* = 7.4 Hz, 2H), 2.32 (s, 3H), 1.97 (s, 3H), 1.76–1.65 (m, 2H), 0.96 (t, *J* = 7.4 Hz, 3H). ^13^C NMR (100 MHz, CDCl_3_): δ 167.0, 159.3, 148.7, 134.3, 129.9, 129.0, 127.4, 124.3, 115.5, 49.9, 21.5, 18.3, 16.1, 11.3. HRMS (ESI) *m/z*: [M + H]^+^ calcd for C_14_H_18_NO_2_, 232.1332; found, 232.1331.

*8-Methoxyl-N-propyl-3-methyl-1,4-benzoxazepin-5(4H)-one* (**2i**): Pale yellow oil (84.1 mg, 68%). ^1^H NMR (400 MHz, CDCl_3_): δ 7.81 (d, *J* = 8.7 Hz, 1H), 6.73 (dd, *J* = 8.7, 2.8 Hz 1H), 6.48 (d, *J* = 2.8 Hz, 1H), 5.43 (s, 1H), 3.83 (s, 3H), 3.55 (t, *J* = 7.4 Hz, 2H), 1.94 (s, 3H), 1.75–1.62 (m, 2H), 0.96 (td, *J* = 7.4, 3.5 Hz, 3H). ^13^C NMR (100 MHz, CDCl_3_): δ 166.2, 163.7, 161.6, 147.7, 133.5, 119.5, 115.1, 111.0, 105.1, 55.7, 49.8, 21.5, 17.8, 11.3. HRMS (ESI) *m/z*: [M + H]^+^ calcd for C_14_H_18_NO_3_, 248.1281; found, 248.1279.

*7-Chloro-N-propyl-3-methyl-1,4-benzoxazepin-5(4H)-one* (**2j**): Pale yellow oil (22.3 mg, 18%). ^1^H NMR (400 MHz, CDCl_3_): δ 7.83 (d, *J* = 2.8 Hz, 1H), 7.34 (dd, *J* = 8.0, 2.8 Hz, 1H), 6.91 (d, *J* = 8.0 Hz, 1H), 5.43 (s, 1H), 3.56 (t, *J* = 7.4 Hz, 2H), 1.93 (s, 3H), 1.70–1.62 (m, 2H), 0.96 (t, *J* = 7.4 Hz, 3H). ^13^C NMR (100 MHz, CDCl_3_): δ 165.3, 159.0, 148.8, 132.9, 131.9, 130.2, 128.6, 121.6, 115.0, 50.0, 21.4, 17.7, 11.3. HRMS (ESI) *m/z*: [M + H]^+^ calcd for C_13_H_15_ClNO_2_, 252.0786; found, 252.0786.

*3-Methyl-1,4-benzoxazepin-5(4H)-one (**2k**)* [23]: Pale yellow oil (26.9 mg, 31%). ^1^H NMR (400 MHz, CDCl_3_): δ 11.18 (s, 1H), 7.71 (dd, *J* = 7.8, 1.6 Hz, 1H), 7.24 (ddd, *J* = 8.3, 7.8, 1.6 Hz, 1H), 6.97 (dd, *J* = 7.8, 1.6 Hz, 1H), 6.88 (ddd, *J* = 8.3, 7.8, 1.6 Hz, 1H), 6.75 (q, *J* = 1.2 Hz, 1H), 2.33 (d, *J* = 1.2 Hz, 3H). ^13^C NMR (100 MHz, CDCl_3_): δ 160.6, 157.1, 147.9, 131.9, 125.7, 122.1, 119.4, 117.2, 111.5, 11.0.

*N-Propyl-2-vinyl-2,3-dihydro-1,3-benzoxazin-4(4H)-one* (**3a**): Pale yellow oil (90.5 mg, 83%). ^1^H NMR (400 MHz, CDCl_3_): δ 7.93 (dd, *J* = 7.8, 1.8 Hz, 1H), 7.41 (apparent td, *J* = 7.8, 1.6 Hz, 1H), 7.07 (apparent td, *J* = 7.8, 1.6 Hz, 1H), 6.91 (dd, *J* = 7.8, 1.8 Hz, 1H), 5.97 (ddd, *J* = 16.8, 10.2, 5.8 Hz, 1H), 5.64 (d, *J* = 5.8 Hz, 1H), 5.40 (d, *J* = 16.8 Hz, 1H), 5.36 (d, *J* = 10.2 Hz, 1H), 3.93 (ddd, *J* = 14.0, 8.0, 6.8 Hz, 1H), 2.97 (ddd, *J* = 14.0, 8.0, 6.8 Hz, 1H), 1.77–1.62 (m, 2H), 0.97 (t, *J* = 7.4 Hz, 3H). ^13^C NMR (100 MHz, CDCl_3_): δ 161.4, 155.4, 134.1, 132.6, 128.0, 122.4, 120.9, 118.7, 116.8, 87.7, 45.8, 21.7, 11.4. HRMS (ESI) *m/z*: [M + H]^+^ calcd for C_13_H_16_NO_2_, 218.1176; found, 218.1175.

*N-Methyl-2-vinyl-2,3-dihydro-1,3-benzoxazin-4(4H)-one* (**3b**) [45]: Pale yellow oil (73.7 mg, 78%). ^1^H NMR (400 MHz, CDCl_3_): δ 7.93 (dd, *J* = 7.8, 1.7 Hz, 1H), 7.41 (apparent td, *J* = 7.8, 1.7 Hz, 1H), 7.07 (apparent td, *J* = 7.8, 1.7 Hz, 1H), 6.92 (dd, *J* = 7.8, 1.7 Hz, 1H), 5.97 (ddd, *J* = 16.8, 10.2, 6.0 Hz, 1H), 5.61 (d, *J* = 6.0 Hz, 1H), 5.45–5.34 (m, 2H), 3.07 (s, 3H). ^13^C NMR (100 MHz, CDCl_3_): δ 161.8, 155.5, 134.2, 131.9, 128.0, 122.4, 121.1, 118.3, 116.8, 89.2, 31.1. HRMS (ESI) *m/z*: [M + H]^+^ calcd for C_11_H_12_NO_2_, 190.0863; found, 190.0861.

*N-Isopropyl-2-vinyl-2,3-dihydro-1,3-benzoxazin-4(4H)-one* (**3c**): Pale yellow oil (89.0 mg, 81%). ^1^H NMR (400 MHz, CDCl_3_): δ 7.92 (dd, *J* = 7.8, 1.6 Hz, 1H), 7.40 (apparent td, *J* = 7.8, 1.6 Hz, 1H), 7.05 (apparent td, *J* = 7.8, 1.6 Hz, 1H), 6.88 (dd, *J* = 7.8, 1.6 Hz, 1H), 5.97 (ddd, *J* = 16.8, 10.0, 6.0 Hz, 1H), 5.78 (d, *J* = 6.0 Hz, 1H), 5.40 (d, *J* = 16.8 Hz, 1H), 5.29 (d, *J* = 10.0 Hz, 1H), 4.85 (hept, *J* = 6.8 Hz, 1H), 1.33 (d, *J* = 6.8 Hz, 3H), 1.21 (d, *J* = 6.8 Hz, 3H). ^13^C NMR (100 MHz, CDCl_3_): δ 160.7, 154.9, 134.6, 133.9, 128.0, 122.2, 120.5, 119.5, 116.7, 82.9, 45.0, 20.9, 20.7. HRMS (ESI) *m/z*: [M + H]^+^ calcd for C_13_H_16_NO_2_, 218.1176; found, 218.1175.

*N-t-Butyl-2-vinyl-2,3-dihydro-1,3-benzoxazin-4(4H)-one* (**3d**): Pale yellow oil (89.9 mg, 78%). ^1^H NMR (400 MHz, CDCl_3_): δ 7.90 (dd, *J* = 7.8, 1.6 Hz, 1H), 7.38 (apparent td, *J* = 7.8, 1.6 Hz, 1H), 7.03 (apparent td, *J* = 7.8, 1.6 Hz, 1H), 6.86 (dd, *J* = 7.8, 1.6 Hz, 1H), 6.05–5.92 (m, 2H), 5.41 (d, *J* = 16.8 Hz, 1H), 5.29 (d, *J* = 10.0 Hz, 1H), 1.56 (s, 9H). ^13^C NMR (100 MHz, CDCl_3_): δ 162.1, 154.7, 134.9, 133.8, 127.9, 122.1, 120.5, 120.4, 116.4, 84.6, 57.4, 28.8. HRMS (ESI) *m/z*: [M + H]^+^ calcd for C_14_H_18_NO_2_, 232.1332; found, 232.1331.

*N-Cyclohexyl-2-vinyl-2,3-dihydro-1,3-benzoxazin-4(4H)-one* (**3g**): White solid (109.6 mg, 85%). mp 84–86 °C. ^1^H NMR (400 MHz, CDCl_3_): δ 7.91 (d, *J* = 7.8 Hz, 1H), 7.39 (apparent t, *J* = 7.8 Hz, 1H), 7.05 (apparent t, *J* = 7.8 Hz, 1H), 6.88 (d, *J* = 7.8 Hz, 1H), 5.95 (ddd, *J* = 16.8, 10.6, 5.7 Hz, 1H), 5.80 (d, *J* = 5.7 Hz, 1H), 5.40 (d, *J* = 16.8 Hz, 1H), 5.28 (d, *J* = 10.6 Hz, 1H), 4.48 (tt, *J* = 12.2, 3.4 Hz, 1H), 1.98–1.01 (m, 10H). ^13^C NMR (100 MHz, CDCl_3_): δ 160.8, 155.0, 134.7, 134.0, 128.1, 122.3, 120.1, 119.7, 116.8, 83.2, 52.9, 31.4, 31.2, 26.0, 25.9, 25.6. HRMS (ESI) *m/z*: [M + H]^+^ calcd for C_16_H_20_NO_2_, 258.1489; found, 258.1487.

*N-Propyl-2-vinyl-8-methyl-2,3-dihydro-1,3-benzoxazin-4(4H)-one* (**3h**): Pale yellow oil (65.8 mg, 54%). ^1^H NMR (400 MHz, CDCl_3_): δ 7.77 (dd, *J* = 7.8, 1.5 Hz, 1H), 7.25 (dd, *J* = 7.8, 1.5 Hz, 1H), 6.95 (apparent t, *J* = 7.8 Hz, 1H), 5.95 (ddd, *J* = 17.0, 10.6, 6.4 Hz, 1H), 5.67 (d, *J* = 6.4 Hz, 1H), 5.38 (d, *J* = 17.0 Hz, 1H), 5.32 (d, *J* = 10.6 Hz, 1H), 3.94 (ddd, *J* = 14.0, 8.0, 6.4 Hz, 1H), 2.97 (ddd, *J* = 14.0, 8.0, 6.4 Hz, 1H), 2.21 (s, 3H), 1.78–1.59 (m, 2H), 0.97 (t, *J* = 7.4 Hz, 3H). ^13^C NMR (100 MHz, CDCl_3_): δ 161.6, 153.5, 135.0, 132.7, 126.0, 125.4, 121.8, 120.4, 118.3, 87.4, 45.7, 21.6, 15.2, 11.4. HRMS (ESI) *m/z*: [M + H]^+^ calcd for C_14_H_18_NO_2_, 232.1332; found, 232.1331.

*7-Methoxyl-N-propyl-2-vinyl-2,3-dihydro-1,3-benzoxazin-4(4H)-one* (**3i**): Pale yellow oil (55.5 mg, 45%). ^1^H NMR (400 MHz, CDCl_3_): δ 7.84 (d, *J* = 8.8 Hz, 1H), 6.62 (dd, *J* = 8.8, 2.4 Hz, 1H), 6.40 (d, *J* = 2.4 Hz, 1H), 5.98 (ddd, *J* = 16.8, 10.4, 5.9 Hz, 1H), 5.61 (d, *J* = 5.9 Hz, 1H), 5.40 (d, *J* = 16.8 Hz, 1H), 5.35 (d, *J* = 10.4 Hz, 1H), 3.91 (ddd, *J* = 14.0, 8.0, 6.8 Hz, 1H), 3.81 (s, 3H), 2.95 (ddd, *J* = 14.0, 8.0, 6.8 Hz, 1H), 1.72–1.58 (m, 2H), 0.96 (t, *J* = 7.4 Hz, 3H). ^13^C NMR (100 MHz, CDCl_3_): δ 164.5, 161.5, 157.1, 132.6, 129.5, 120.8, 111.8, 109.4, 101.1, 88.0, 55.7, 45.6, 21.7, 11.4. HRMS (ESI) *m/z*: [M + H]^+^ calcd for C_14_H_18_NO_3_, 248.1281; found, 248.1279.

*6-Chloro-N-propyl-2-vinyl-2,3-dihydro-1,3-benzoxazin-4(4H)-one* (**3j**): Pale yellow oil (76.2 mg, 61%). ^1^H NMR (400 MHz, CDCl_3_): δ 7.89 (d, *J* = 2.7 Hz, 1H), 7.35 (dd, *J* = 8.7, 2.7 Hz, 1H), 6.86 (d, *J* = 8.7 Hz, 1H), 5.94 (ddd, *J* = 17.0, 10.3, 5.5 Hz, 1H), 5.65 (d, *J* = 5.5 Hz, 1H), 5.40 (d, *J* = 17.0 Hz, 1H), 5.38 (d, *J* = 10.3 Hz, 1H), 3.92 (ddd, *J* = 14.0, 8.4, 7.2 Hz, 1H), 2.96 (ddd, *J* = 14.0, 8.4, 7.2 Hz, 1H), 1.72–1.63 (m, 2H), 0.97 (t, *J* = 7.4 Hz, 3H). ^13^C NMR (100 MHz, CDCl_3_): δ 160.3, 153.9, 133.9, 132.2, 127.8, 127.7, 121.3, 119.9, 118.4, 87.7, 46.0, 21.6, 11.4. HRMS (ESI) *m/z*: [M + H]^+^ calcd for C_13_H_15_ClNO_2_, 252.0786; found, 252.0785.

*6-Nitro-N-propyl-2-vinyl-2,3-dihydro-1,3-benzoxazin-4(4H)-one* (**3l**): Pale yellow oil (85.4 mg, 65%). ^1^H NMR (400 MHz, CDCl_3_): δ 8.83 (d, *J* = 2.8 Hz, 1H), 8.30 (dd, *J* = 8.9, 2.8 Hz, 1H), 7.05 (d, *J* = 8.9 Hz, 1H), 5.94 (ddd, *J* = 17.0, 10.3, 5.4 Hz, 1H), 5.78 (dd, *J* = 5.4, 0.8 Hz, 1H), 5.44 (dd, *J* = 17.0, 0.8 Hz, 1H), 5.44 (d, *J* = 10.3 Hz, 1H), 3.97 (ddd, *J* = 14.0, 8.2, 6.8 Hz, 1H), 3.01 (ddd, *J* = 14.0, 8.2, 6.8 Hz, 1H), 1.83–1.67 (m, 2H), 0.98 (t, *J* = 7.4 Hz, 3H). ^13^C NMR (100 MHz, CDCl_3_): δ 159.9, 159.5, 143.0, 131.6, 129.2, 124.5, 121.9, 118.8, 118.0, 88.2, 46.2, 21.5, 11.4. HRMS (ESI) *m/z*: [M + H]^+^ calcd for C_13_H_15_N_2_O_4_, 263.1026; found, 263.1023.

*6-Trifluoromethyl-N-propyl-2-vinyl-2,3-dihydro-1,3-benzoxazin-4(4H)-one* (**3m**): Pale yellow oil (103.8 mg, 73%). ^1^H NMR (400 MHz, CDCl_3_): δ 8.22 (d, *J* = 2.1 Hz, 1H), 7.63 (dd, *J* = 8.6, 2.1 Hz, 1H), 7.01 (d, *J* = 8.6 Hz, 1H), 5.94 (ddd, *J* = 16.4, 10.4, 5.5 Hz, 1H), 5.71 (d, *J* = 5.5 Hz, 1H), 5.40 (d, *J* = 16.4 Hz, 1H), 5.40 (d, *J* = 10.4 Hz, 1H), 3.95 (ddd, *J* = 14.1, 8.0, 6.5 Hz, 1H), 2.98 (ddd, *J* = 14.1, 8.0, 6.5 Hz, 1H), 1.72–1.62 (m, 2H), 0.97 (t, *J* = 7.4 Hz, 3H). ^13^C NMR (100 MHz, CDCl_3_): δ 160.2, 157.7, 132.0, 130.84 (q, *J* = 13.0 Hz), 125.8 (q, *J* = 13.0 Hz), 124.9 (q, *J* = 34.0 Hz), 123.9 (q, *J* = 270.0 Hz), 121.5, 118.7, 117.6, 87.90, 46.02, 21.55, 11.36. HRMS (ESI) *m/z*: [M + H]^+^ calcd for C_14_H_15_F_3_NO_2_, 286.1049; found, 286.1046.

## 4. Conclusions

In summary, a facile and efficient solvent-controlled chemodivergent synthesis of 1,4-benzoxazepin-5(4*H*)-ones and 1,3-benzoxazin-4(4*H*)-ones via the KOH-promoted cyclization of *ortho*-fluorobenzamides with 2-propyn-1-ol was developed. The cyclization reaction was proposed to involve the S_N_Ar reaction of C–F bond with 2-propyn-1-ol to give *ortho*-[(2-propynyl)oxy]benzamide intermediates, which underwent the intramolecular either *7-exo-dig* cyclization in a superbase medium of KOH/DMSO or a Michael addition of N–H to allenyl intermediate in KOH/MeCN medium to give different benzo-fused cyclic compounds. The present protocol had the significant advantages of high atom-utilization and high selectivity of product output controlled by simple changing the solvents. 

## Figures and Tables

**Table 1:**
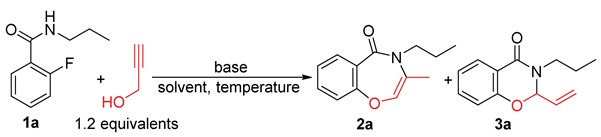
Effects of reaction conditions on the chemodivergent formation of 1,4-benzoxazepin-5(4*H*)-ones and 1,3-benzoxazin-4(4*H*)-ones *^a^*.

Entry	Base (equivalent)	Solvent	Temperature (°C) /Time (h)	Yield (%) *^b^*
1	KOH (3)	DMSO	50/12	45% (**2a**) + 12% (**3a**)
2	KOH (3)	DMSO	30/12	52% (**2a**) + 7% (**3a**)
3	KOH (3)	DMSO	30/12 + 50/12	54% (**2a**)
4 *^c^*	KOH (3)	DMSO	30/12 + 50/12	45% (**2a**)
5 *^d^*	KOH (3)	DMSO	30/12 + 50/12	47% (**2a**)
6	KOH (3)	DMSO	30/12 + 70/12	41% (**2a**)
7	KOH (1)	DMSO	30/12 + 50/12	44% (**2a**)
8	KOH (2)	DMSO	30/12 + 50/12	47% (**2a**)
9	NaOH (3)	DMSO	30/12 + 50/12	33% (**2a**)
10	K_2_CO_3_ (3)	DMSO	30/12 + 50/12	~ 10% (**2a**)
11	Cs_2_CO_3_ (3)	DMSO	30/12 + 50/12	comlex mixture
12	*t*-BuOK (3)	DMSO	30/12 + 50/12	complex mixture
13 *^e^*	KOH (3)	DMAc	30/12 + 50/12	52% (**2a**) + 26% (**3a**)
14	KOH (3)	1,4-dioxane	30/12 + 50/12	35% (**2a**) + ~10% (**3a**)
15	KOH (3)	THF	30/12 + 50/12	~10% (**2a**) + 48% (**3a**)
16	KOH (3)	DMF	30/12 + 50/12	~10% (**2a**) + 48% (**3a**)
17	KOH (3)	MeCN	30/12 + 50/12	trace (**2a**) + 83% (**3a**)
18	KOH (3)	MeCN	30/12 + 50/12	12% (**2a**) + 53% (**3a**)
19^*d*^	KOH (3)	DMSO/MeCN (1:1 in volume)	30/12 + 50/12	trace (**2a**) + 79% (**3a**)
20	KOH (3)	MeCN	30/12 + 70/12	trace (**2a**) + 62% (**3a**)

*^a^* Unless otherwise noted, the reactions were carried out using 0.5 mmol of **1a**, 0.6 mmol of 2-propyn-1-ol, and 1.5 mmol of base in 4.0 mL of solvent. *^b^* Isolated yields. ^c^ 0.5 mmol of 2-propyn-1-ol was used. *^d^* 0.75 mmol of 2-propyn-1-ol was used. *^e^* DMAc: *N*,*N*-dimethyl acetamide.

## Supplementary Materials

The following are available online: Synthesis of known compound **1**, copies of ^1^H NMR spectra of the prepared **1**, copies of NMR spectra of **2**, copies of NMR spectra of **3**, X-ray structural details of **2g** and **3g**, results from the reactions of **1a** with propargyl alcohol in either KOD/D_2_O/DMSO or KOD/D_2_O/MeCN.
