# Supplementary material for: Base-Promoted Chemodivergent Formation of 1,4-Benzoxazepin-5(4H)-ones and 1,3-Benzoxazin-4(4H)-ones Switched by Solvents
Source: Molecules. 2019 Oct 19;24(20):3773. doi: 10.3390/molecules24203773 (PMC6832296; doi:10.3390/molecules24203773)
Supplement: Supplementary file 1 [file molecules-24-03773-s001.pdf]

Supplementary File

Base-Promoted Chemodivergent Formation of 1,4-Benzoxazepin-5(4*H*)-ones and 1,3-Benzoxazin-4(4*H*)-ones Switched by Solvents

Qian Chen, Yunpeng Wang and Ruimao Hua *

Department of Chemistry, Tsinghua University, Key Laboratory of Organic Optoelectronics & Molecular Engineering of Ministry of Education, Beijing 100084, China

E-mail: ruimao@mail.tsinghua.edu.cn

Contents

Contents	Page
1. Synthesis of known compound **1**	S02
2. Copies of ^{1}H NMR spectra of the prepared **1**	S04
3. Copies of NMR spectra of **2**	S16
4. Copies of NMR spectra of **3**	S27
5. X-ray structural details of **2g** and **3g**	S37
6. Results from the reactions of **1a** with propargyl alcohol in either KOD/D_2O/DMSO or KOD/D_2O/MeCN	S55

1. Synthesis of known compound 1 (a modified literature method: [1])

A typical procedure for the synthesis of **1a**:

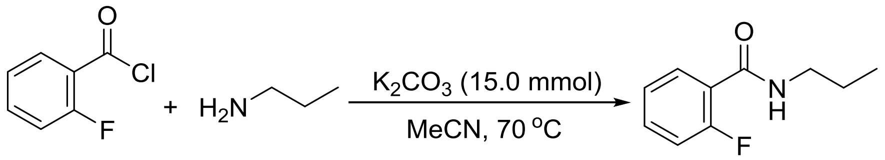

10.0 mmol 15.0 mmol **1a**

A mixture of propylamine (0.885 g, 15.0 mmol), K_2CO_3 (2.070 g, 15.0 mmol) and CH_3CN (25 mL) in a 100-mL round-bottomed flask was stirred at 70 ℃ for a few minutes, and then 2-fluorobenzoyl chloride (1.585 g, 10.0 mmol) was added dropwise to the mixture (*ca.* 0.5 h). The obtained reaction mixture was heated for additional 4 h and then cooled to room temperature, water (50 mL) was added and the mixture was extracted with ethyl acetate (3 × 50 mL). The combined organic phases were dried over $MgSO_4$.The filtered solution was concentrated under reduced pressure, and the crude residue was purified by column chromatography on silica gel with the use of petroleum ether/ethyl acetate (gradient mixture ratio from 20:1 to 4:1 in volume) to afford **1a** as a pale yellow oil in 90% yield (1.635 g).

1b ~ **1m** were prepared in 2.0 mmol-scale of benzoyl chlorides.

Table S1. Yields in both weight and percentage for known compound **1** [a].

Compound	Weight (g)	Yield (%)	Compound	Weight (g)	Yield (%)
**1a** [b]	1.635	90	**1g**	0.383	88
**1b**	0.279	91	**1h**	0.358	92
**1c**	0.325	90	**1i**	0.393	93
**1d**	0.333	85	**1j**	0.348	81
**1e**	0.330	85	**1l**	0.351	78
**1f**	0.353	87	**1m**	0.390	78

[a] Unless otherwise noted, the reactions were carried out by using 2.0 mmol of benzoyl chlorides and 3.0 mmol of amines.

[b] 2-Fluorobenzoyl chloride and *n*-propylamine were used in 10.0 mmol and 15.0 mmol, respectively. Twice reactions were performed, and the yields are almost same.

Structures of Compound **1**

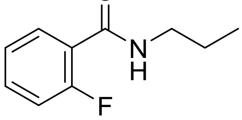

1a

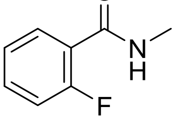

1b

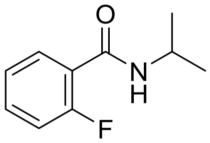

1c

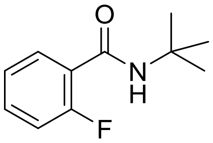

1d

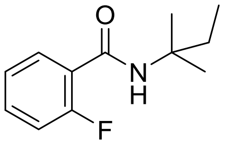

1e

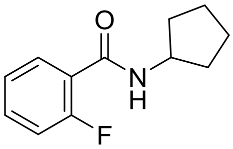

1f

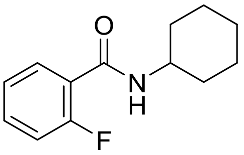

1g

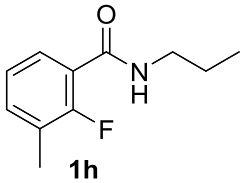

1h

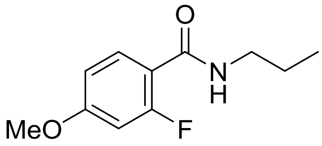

1i

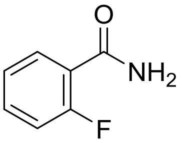

1k

(*commercial product*)

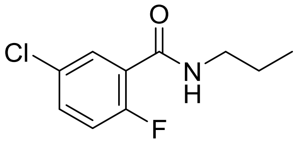

1j

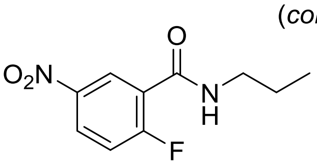

1l

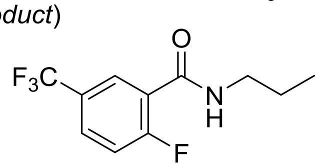

1m

2. Copies of ^{1}H NMR spectra of the prepared 1

^{1}H NMR spectrum of **1a**

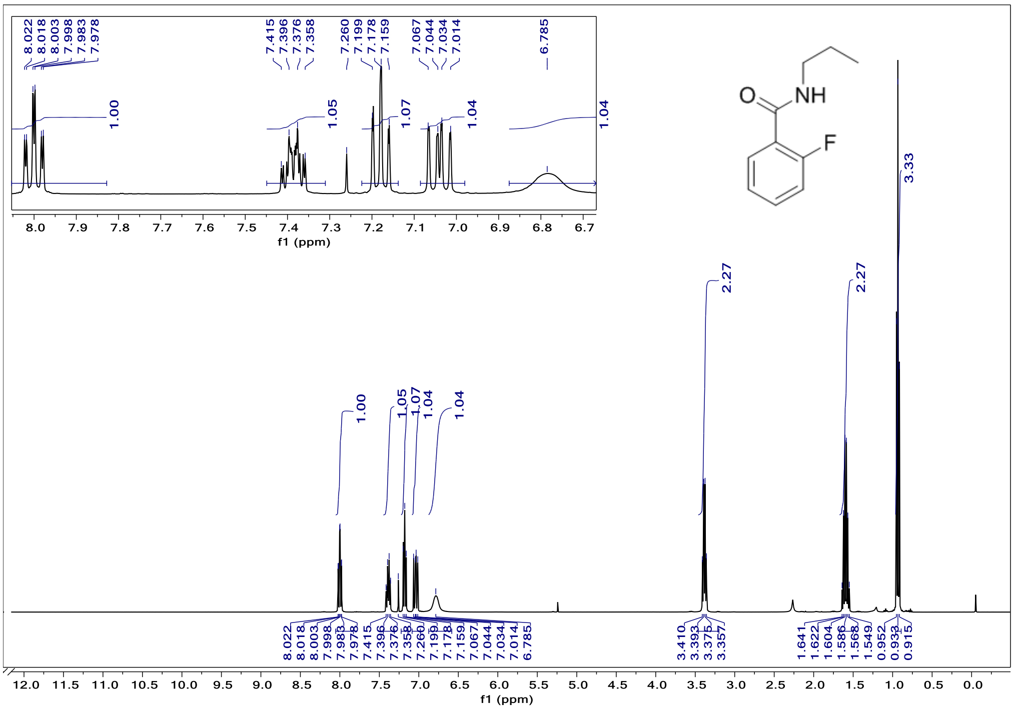

2-Fluoro-N-propylbenzamide (**1a**): ^{1}H NMR (400 MHz, $CDCl_3$): δ 8.00 (apparent td, *J* = 7.8, 1.8 Hz, 1H), 7.35-7.42 (m, 1H), 7.18 (apparent td, *J* = 7.8, 0.8 Hz, 1H), 7.06-7.01 (m, 1H), 6.79 (s_{br}, 1H), 3.38 (tq, *J* = 7.4, 1.6 Hz, 2H), 1.59 (apparent hex, *J* = 7.4 Hz, 2H), 0.93 (t, *J* = 7.4 Hz, 3H).

^{1}H NMR spectrum of **1b**

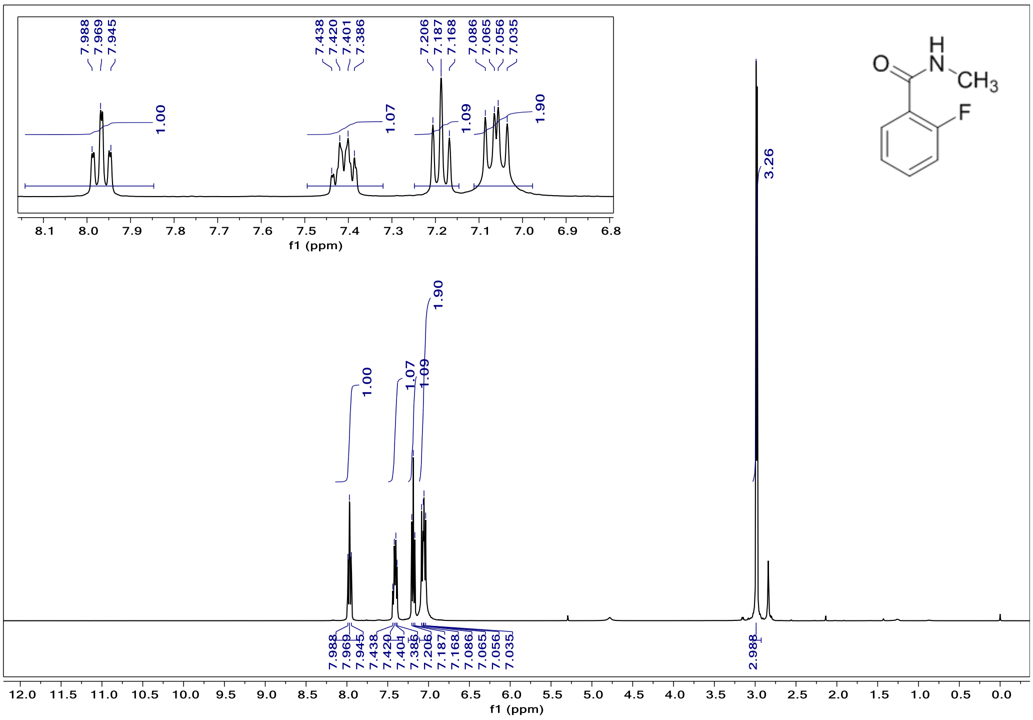

2-Fluoro-N-methylbenzamide (**1b**): ^{1}H NMR (400 MHz, $CDCl_3$): δ 7.97 (apparent td, *J* = 7.8, 1.8 Hz, 1H), 7.37-7.44 (m, 1H), 7.19 (t, *J* = 7.8 Hz, 1H), 7.08-7.03 (m, 2H), 7.06 (s, 1H), 2.99 (d, *J* = 4.8 Hz, 3H).

^{1}H NMR spectrum of **1c**

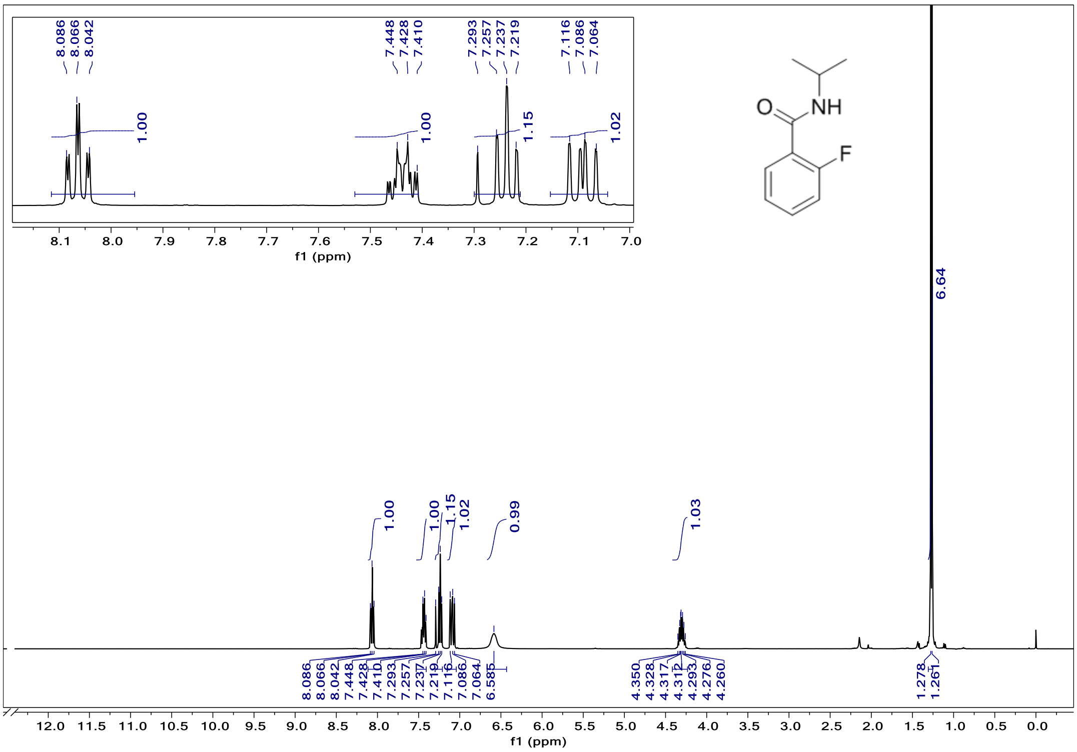

2-Fluoro-N-isopropylbenzamide (**1c**): ^{1}H NMR (400 MHz, $CDCl_3$): δ 8.06 (apparent td, *J* = 8.0, 1.8 Hz, 1H), 7.41-7.46 (m, 1H), 7.24 (t, *J* = 8.0 Hz, 1H), 7.11-7.06 (m, 1H), 6.59 (s_{br}, 1H), 4.26-4.35 (m, 1H), 1.27 (d, *J* = 7.0 Hz, 6H).

^{1}H NMR spectrum of **1d**

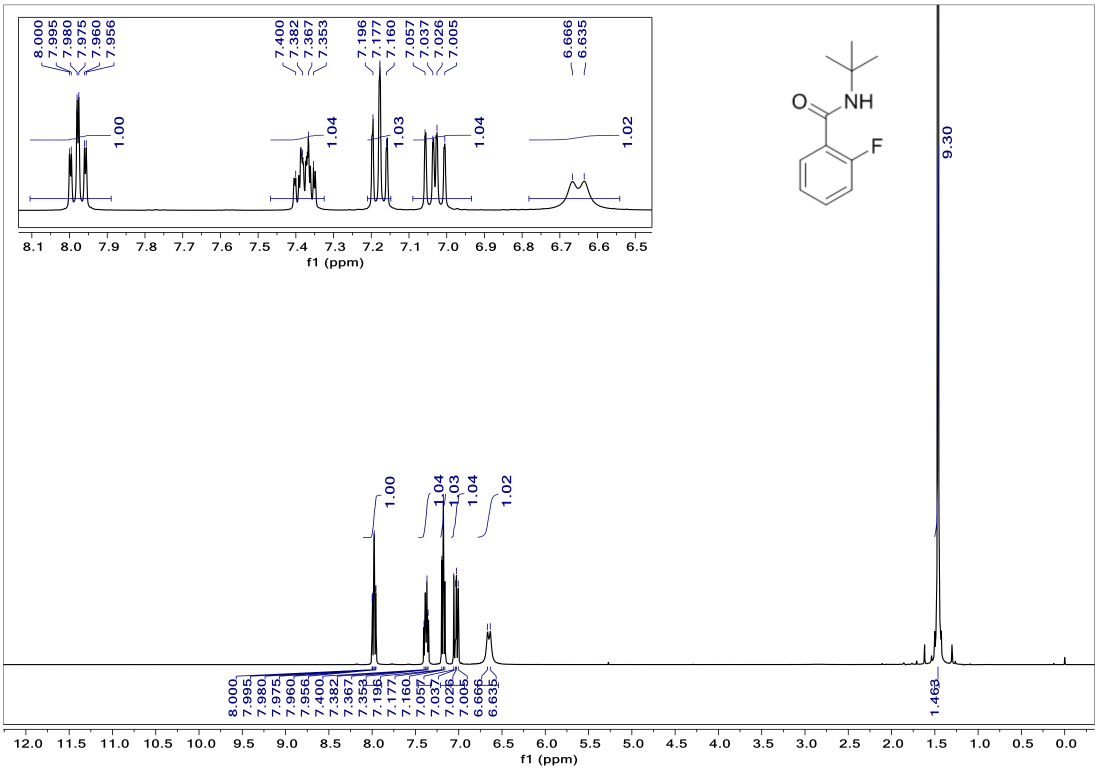

2-Fluoro-N-t-butylbenzamide (**1d**): ^{1}H NMR (400 MHz, $CDCl_3$): δ 7.98 (apparent td, *J* = 8.0, 1.8 Hz, 1H), 7.34-7.41 (m, 1H), 7.18 (apparent td, *J* = 8.0, 0.8 Hz, 1H), 7.05-7.00 (m, 1H), 6.65 (d, *J* = 12.4 Hz, 1H), 1.46 (s, 9H).

^{1}H NMR spectrum of **1e**

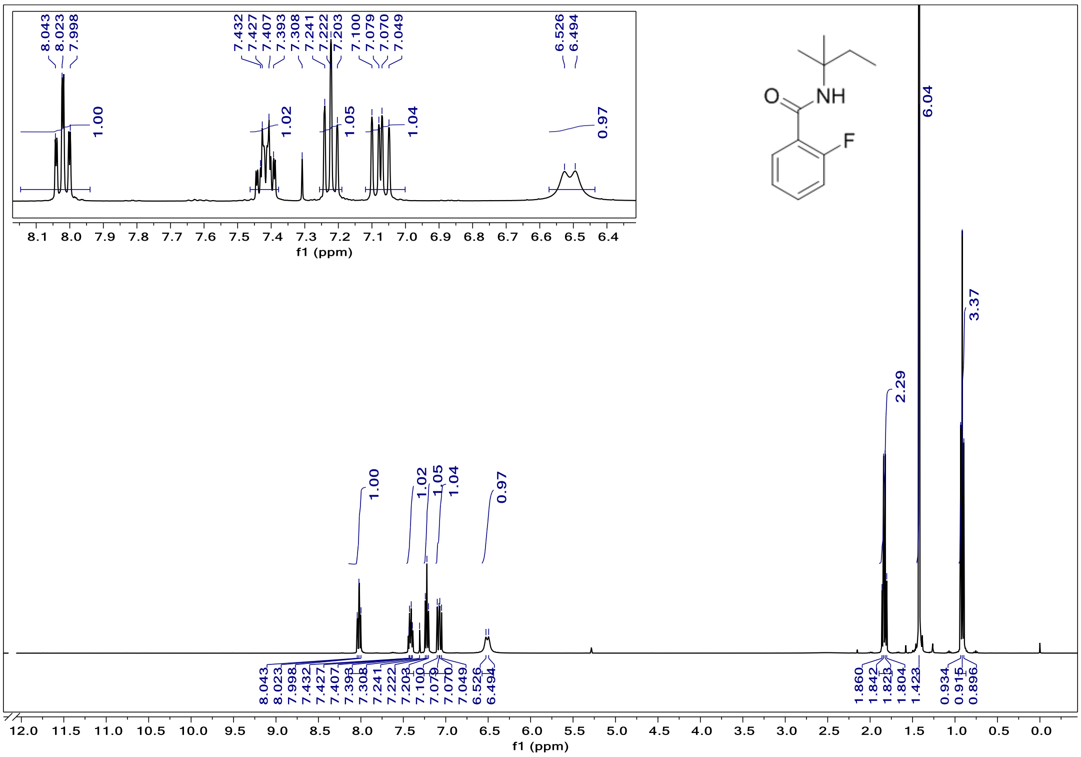

2-Fluoro-N-t-amylbenzamide (**1e**): ^{1}H NMR (400 MHz, $CDCl_3$): δ 8.02 (apparent td, *J* = 8.0, 1.8 Hz, 1H), 7.38-7.45 (m, 1H), 7.22 (t, *J* = 8.0 Hz, 1H), 7.10-7.05 (m, 1H), 6.51 (d, *J* = 12.8 Hz, 1H), 1.83(q, *J* = 7.8 Hz, 2H), 1.42 (s, 6H), 0.92 (t, *J* = 7.8 Hz, 3H).

^{1}H NMR spectrum of **1f**

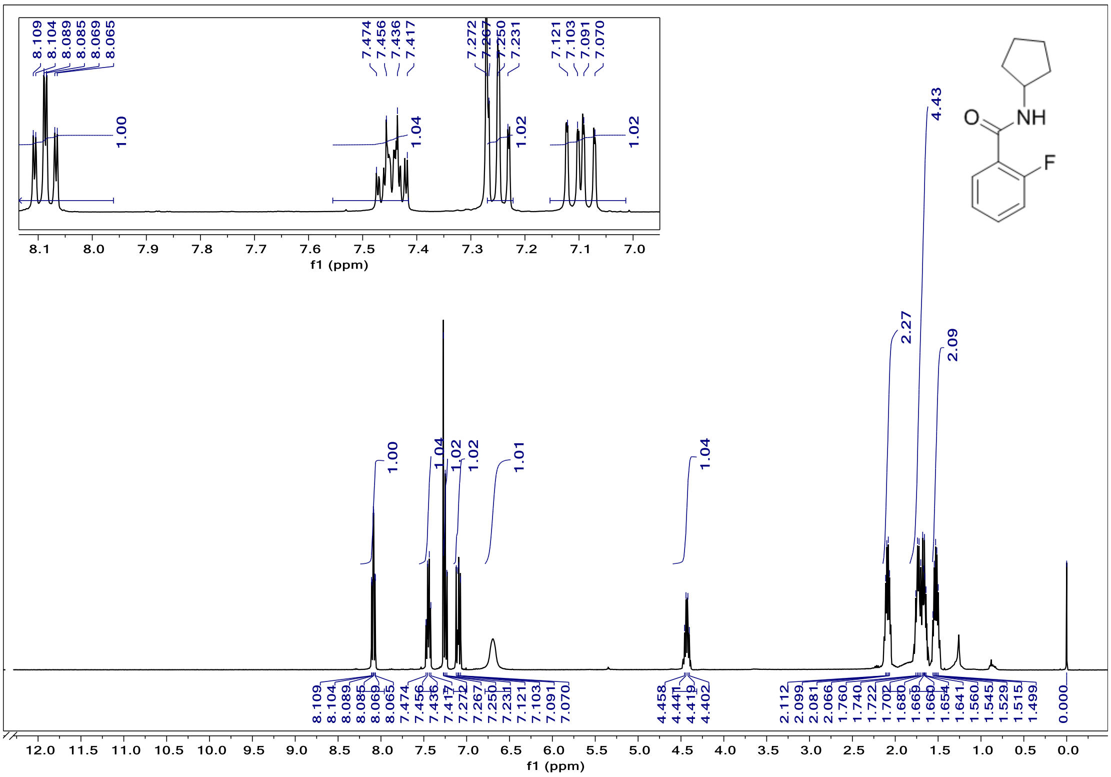

2-Fluoro-N-cyclopentylbenzamide (**1f**): ^{1}H NMR (400 MHz, $CDCl_3$): δ 8.09 (apparent td, *J* = 8.0, 1.8 Hz, 1H), 7.41-7.48 (m, 1H), 7.25 (apparent td, *J* = 8.0, 0.8 Hz, 1H), 7.12-7.07 (m, 1H), 6.69 (s_{br}, 1H), 4.38-4.48 (m, 1H), 2.04-2.14 (m, 2H), 1.60-1.80 (m, 4H), 1.48-1.56 (m, 2H).

^{1}H NMR spectrum of **1g**

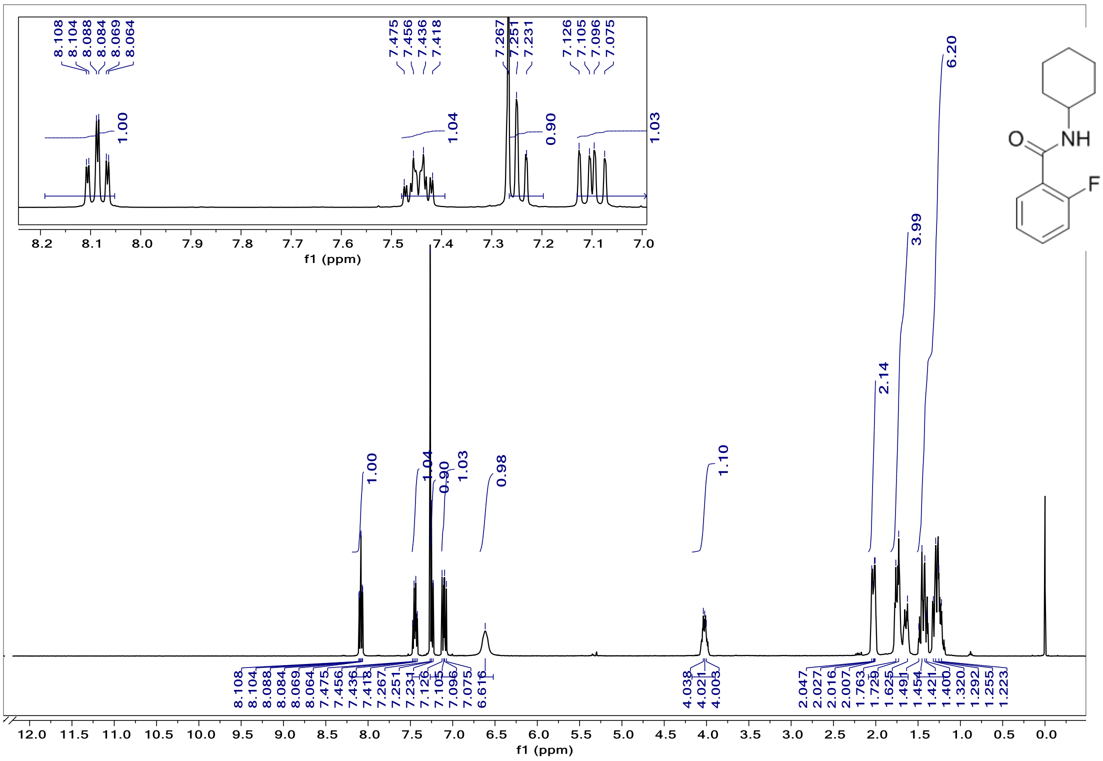

2-Fluoro-N-cyclohexylbenzamide (**1g**): ^{1}H NMR (400 MHz, $CDCl_3$): δ 8.09 (apparent td, *J* = 8.0, 1.8 Hz, 1H), 7.41-7.48 (m, 1H), 7.25 (apparent td, *J* = 8.0, 0.8 Hz, 1H), 7.12-7.07 (m, 1H), 6.62 (s_{br}, 1H), 3.98-4.07 (m, 1H), 1.99-2.06 (m, 2H), 1.60-1.78 (m, 4H), 1.18-1.50 (m, 4H).

^{1}H NMR spectrum of **1h**

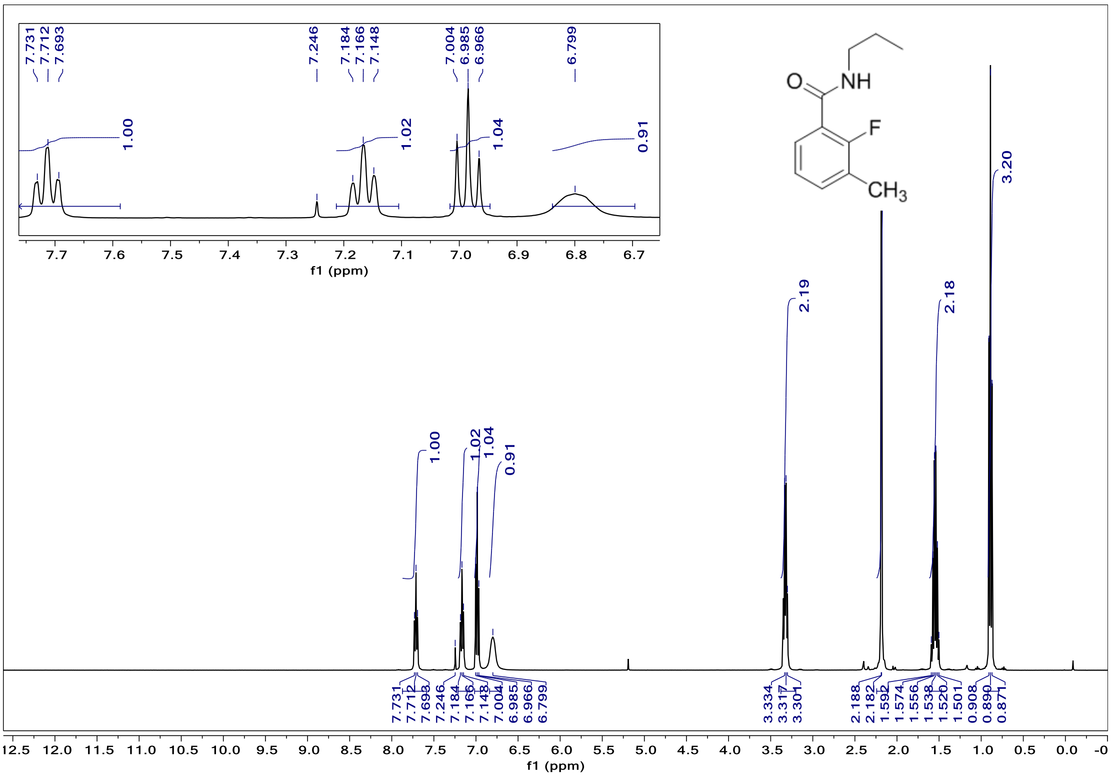

2-Fluoro-N-propyl-3-methylbenzamide (**1h**): ^{1}H NMR (400 MHz, $CDCl_3$): δ 8.00 (apparent t, J = 7.8 Hz, 1H), 7.17 (apparent t, J = 7.8 Hz, 1H), 6.99 (apparent t, J = 7.8 Hz, 1H), 6.80 (s_{br}, 1H), 3.33 (tq, J = 7.3, 1.2 Hz, 2H), 2.18 (d, J = 2.4 Hz, 3H), 1.55 (apparent hex, J = 7.3 Hz, 2H), 0.89 (t, J = 7.3 Hz, 3H).

^{1}H NMR spectrum of **1i**

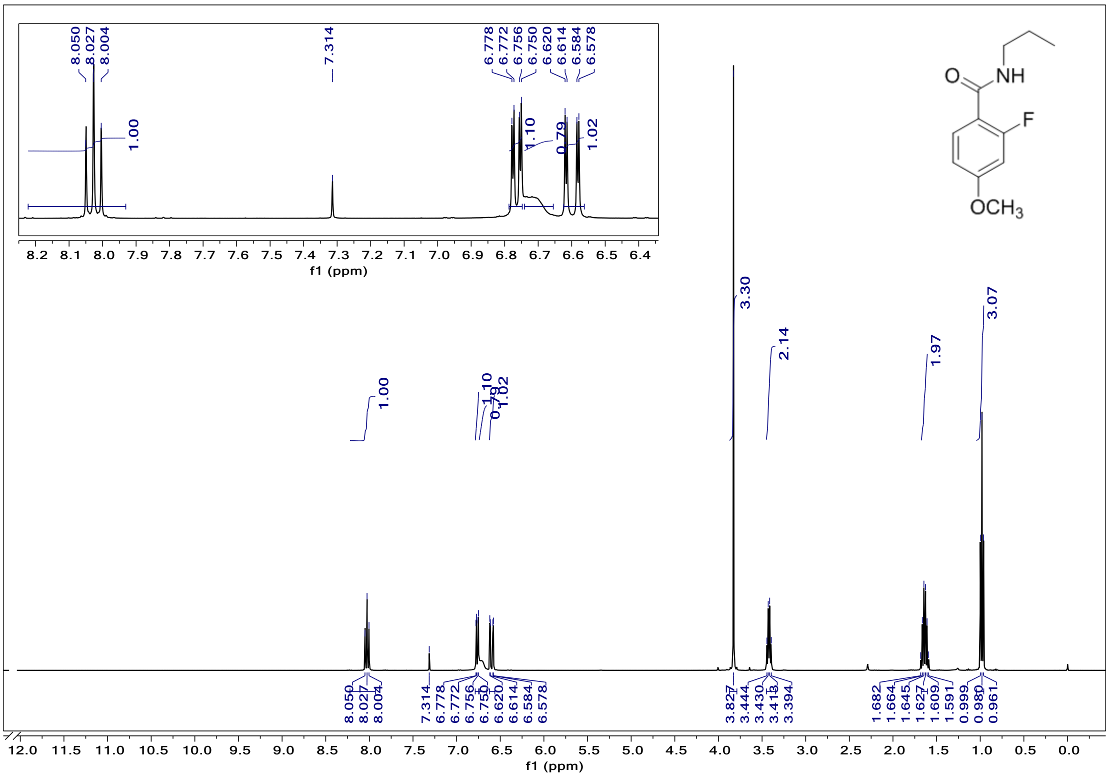

2-Fluoro-4-methoxyl-N-propylbenzamide (**1i**): ^{1}H NMR (400 MHz, $CDCl_3$): δ 8.03 (apparent t, J = 9.6 Hz, 1H), 6.76 (dd, J = 9.6, 2.4 Hz, 1H), 6.71 (s_{br}, 1H), 6.59 (dd, J = 9.6, 2.4 Hz, 1H), 3.83 (s, 3H), 3.42 (tq, J = 7.6, 1.2 Hz, 2H), 1.64 (apparent hex, J = 7.6 Hz, 2H), 0.98 (t, J = 7.6 Hz, 3H).

^{1}H NMR spectrum of **1j**

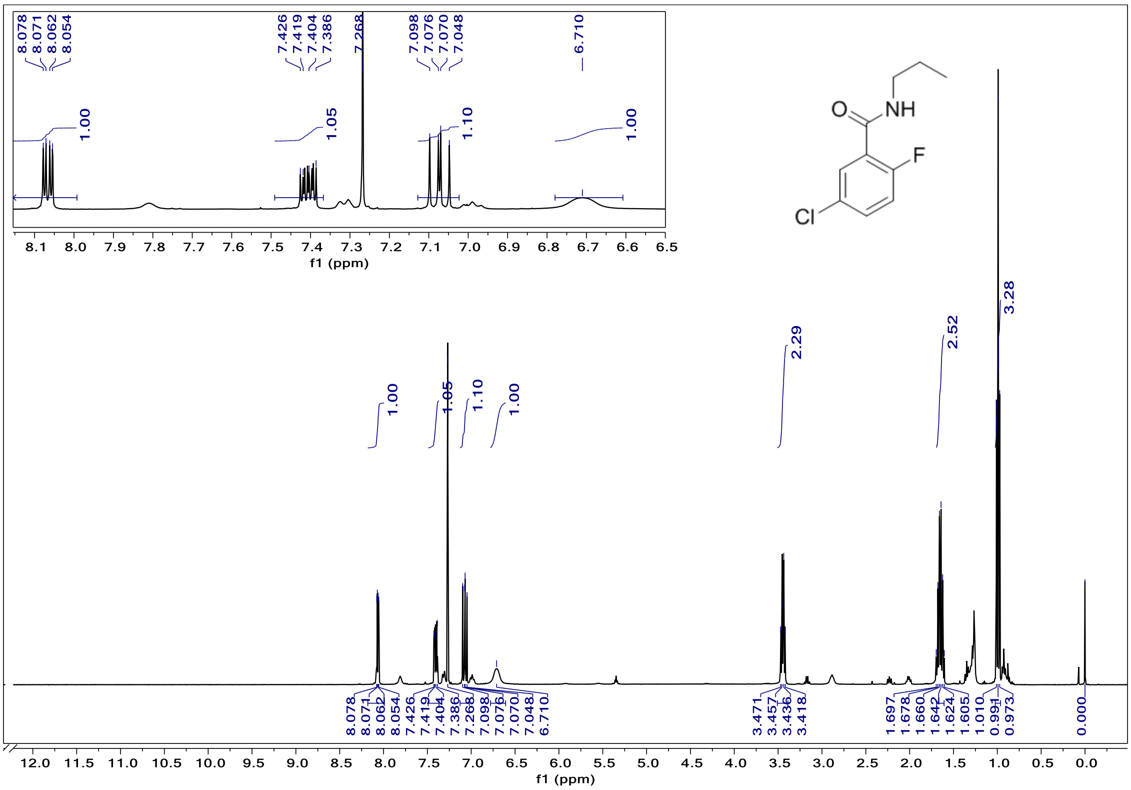

2-Fluoro-5-chloro-N-propylbenzamide (**1j**): ^{1}H NMR (400 MHz, $CDCl_3$): δ 8.07 (dd, J = 6.8, 3.0 Hz, 1H), 7.41 (ddd, J = 8.8, 4,4, 3.0 Hz, 1H), 7.07 (dd, J = 10.4, 8.8 Hz, 1H), 6.71 (s_{br}, 1H), 3.44 (tq, J = 7.6, 1.2 Hz, 2H), 1.65 (apparent hex, J = 7.6 Hz, 2H), 0.99 (t, J = 7.6 Hz, 3H).

^{1}H NMR spectrum of **1l**

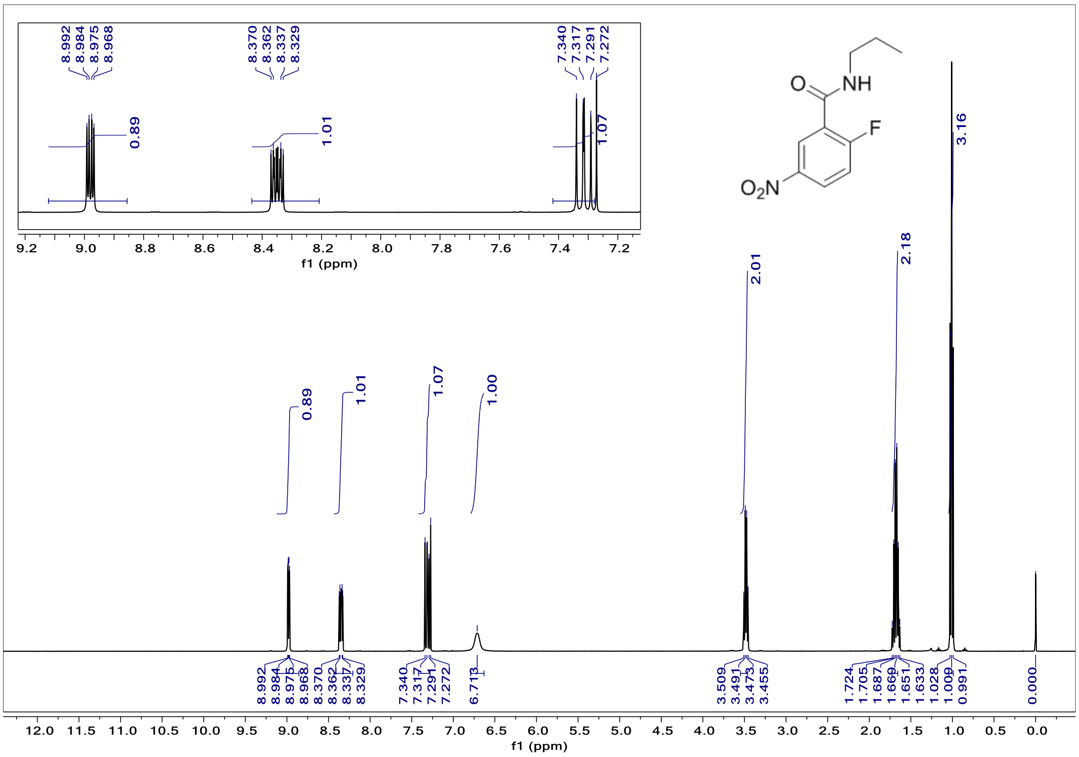

2-Fluoro-5-nitro-N-propylbenzamide (**1l**): ^{1}H NMR (400 MHz, $CDCl_3$): δ 8.98 (dd, *J* = 6.8, 3.0 Hz, 1H), 8.35 (ddd, *J* = 8.8, 4,4, 3.0 Hz, 1H), 7.31 (dd, *J* = 10.4, 8.8 Hz, 1H), 6.71 (s_{br}, 1H), 3.48 (tq, *J* = 7.6, 1.2 Hz, 2H), 1.68 (apparent hex, *J* = 7.6 Hz, 2H), 1.01 (t, *J* = 7.6 Hz, 3H).

^{1}H NMR spectrum of **1m**

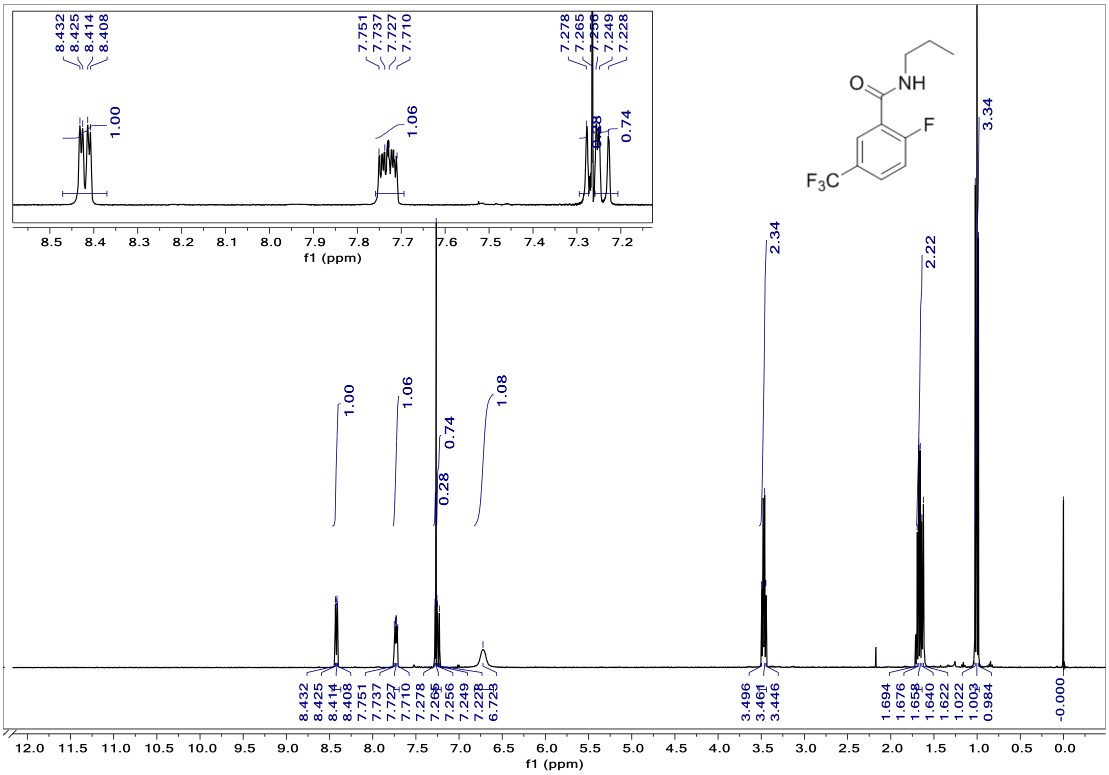

2-Fluoro-5-trifluoromethyl-N-propylbenzamide (**1m**): ^{1}H NMR (400 MHz, $CDCl_3$): δ 8.44 (dd, J = 6.8, 2.8 Hz, 1H), 7.7,-7.75 (m, 1H), 7.22-7.28 (m, 1H), 6.72 (s_{br}, 1H), 3.47 (tq, J = 7.6, 1.2 Hz, 2H), 1.67 (apparent hex, J = 7.6 Hz, 2H), 1.00 (t, J = 7.6 Hz, 3H).

3. Copies of NMR spectra of 2

^{1}H NMR spectrum of **2a**

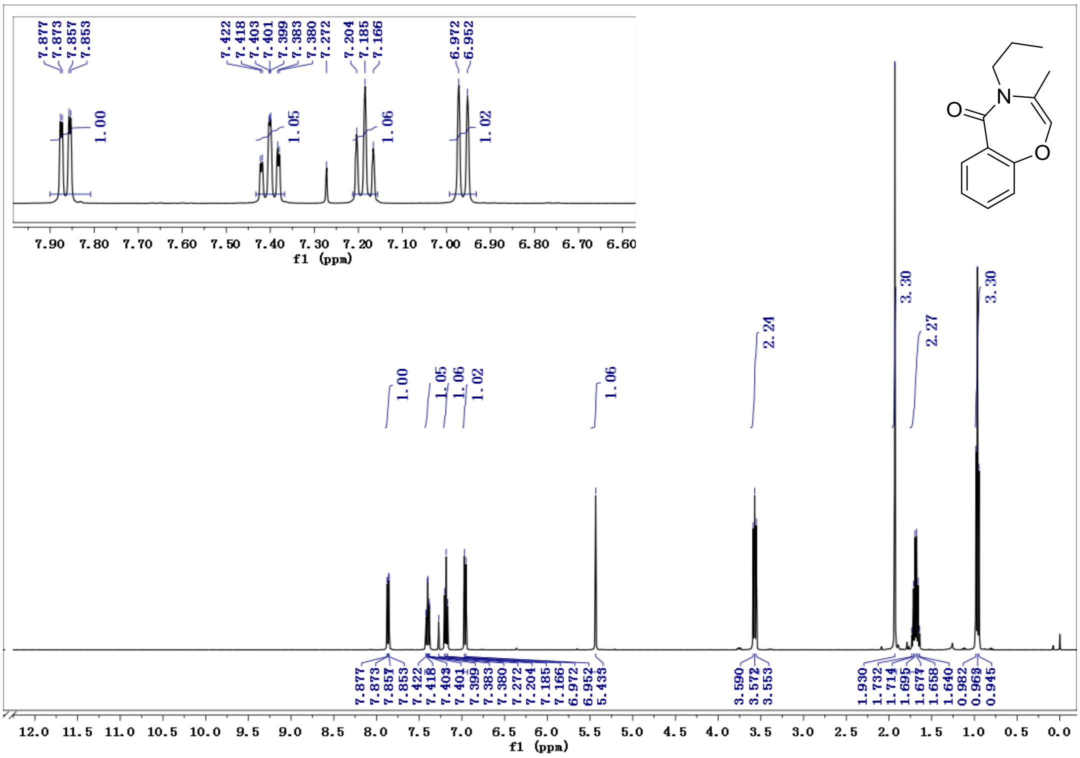

^{13}C NMR spectrum of **2a**

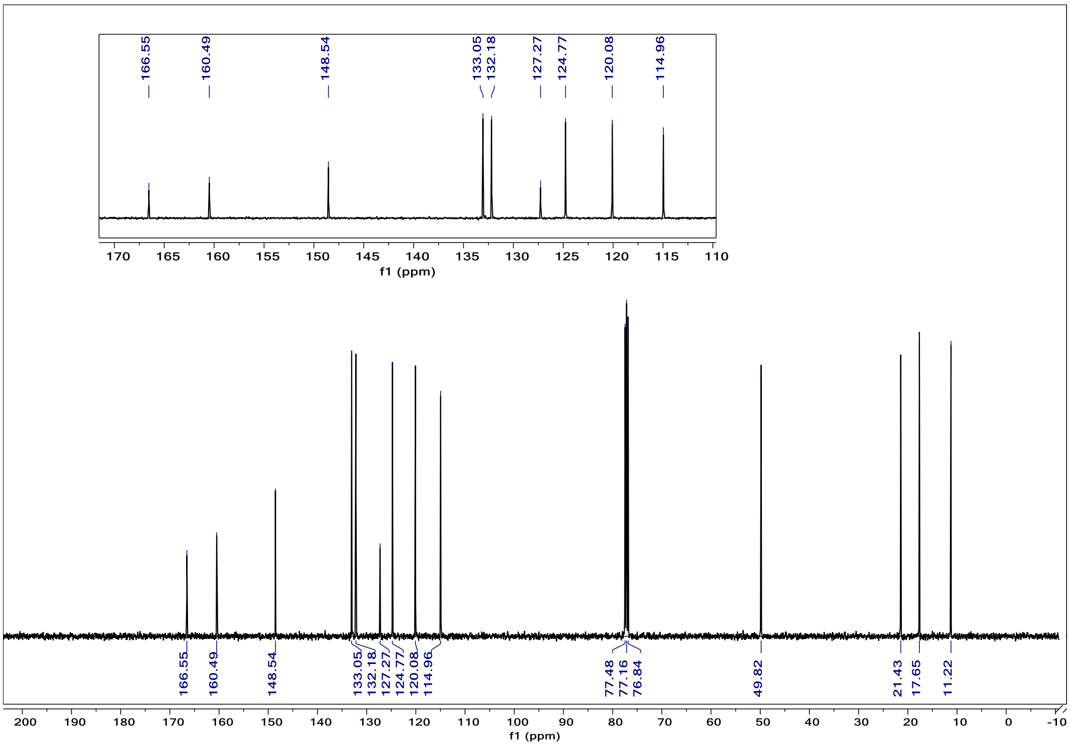

^{1}H NMR spectrum of **2b**

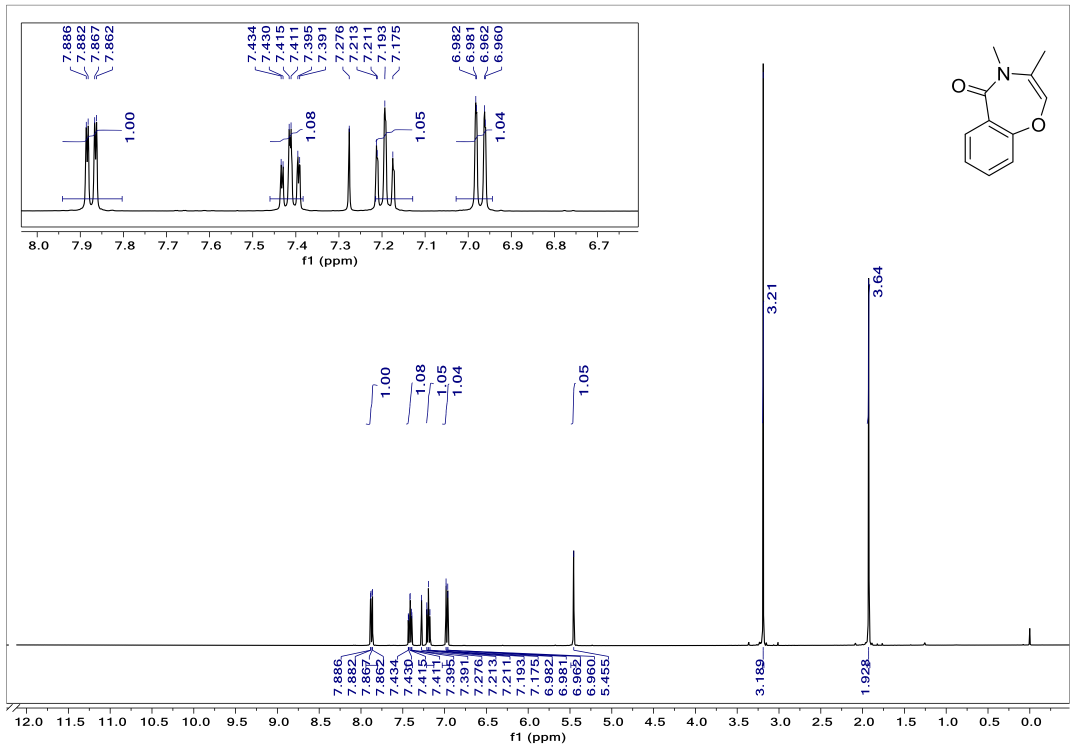

^{13}C NMR spectrum of **2b**

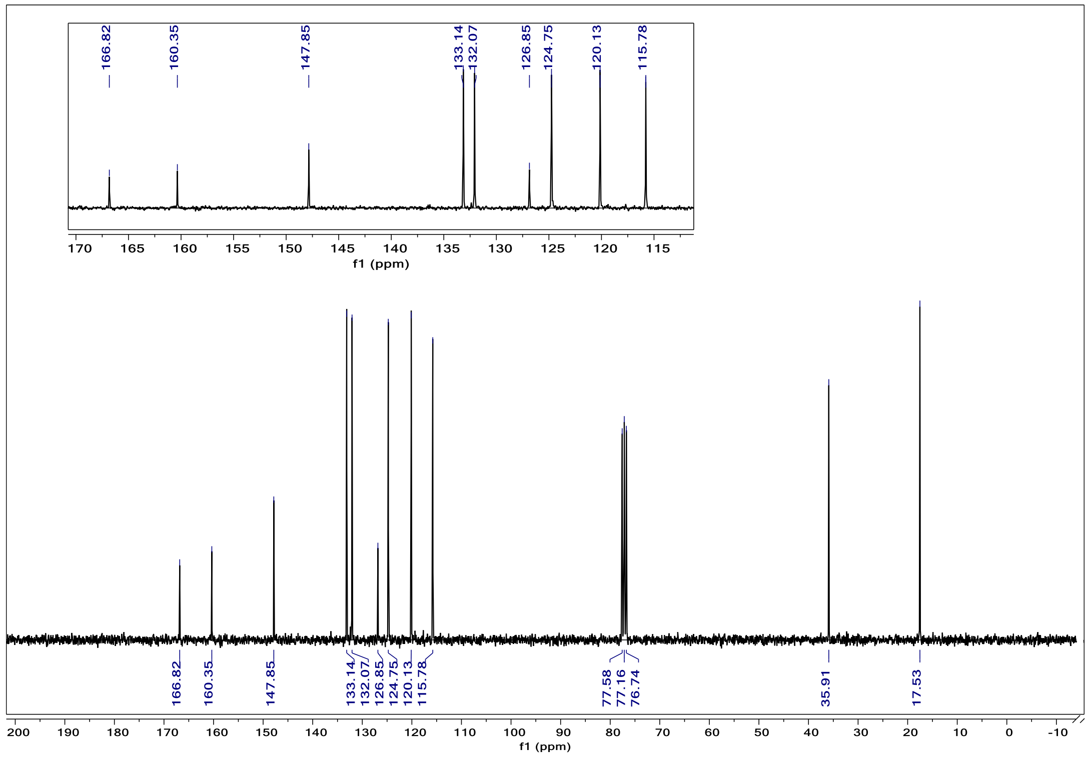

^{1}H NMR spectrum of **2c**

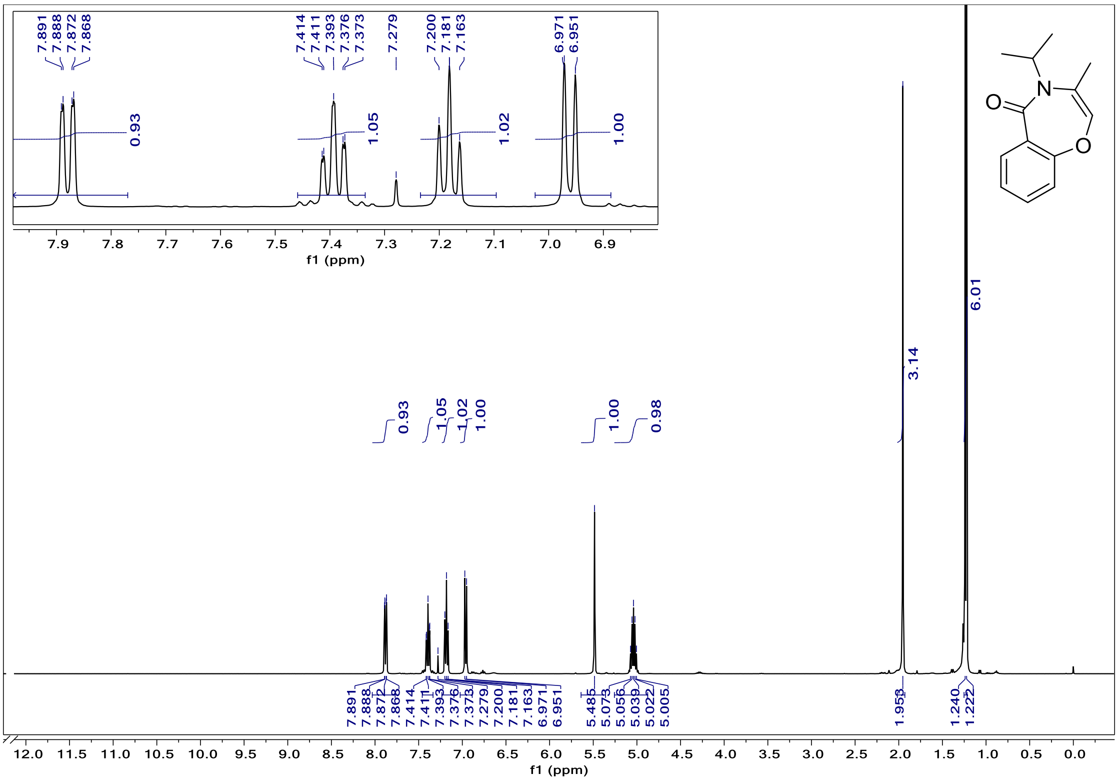

^{13}C NMR spectrum of **2c**

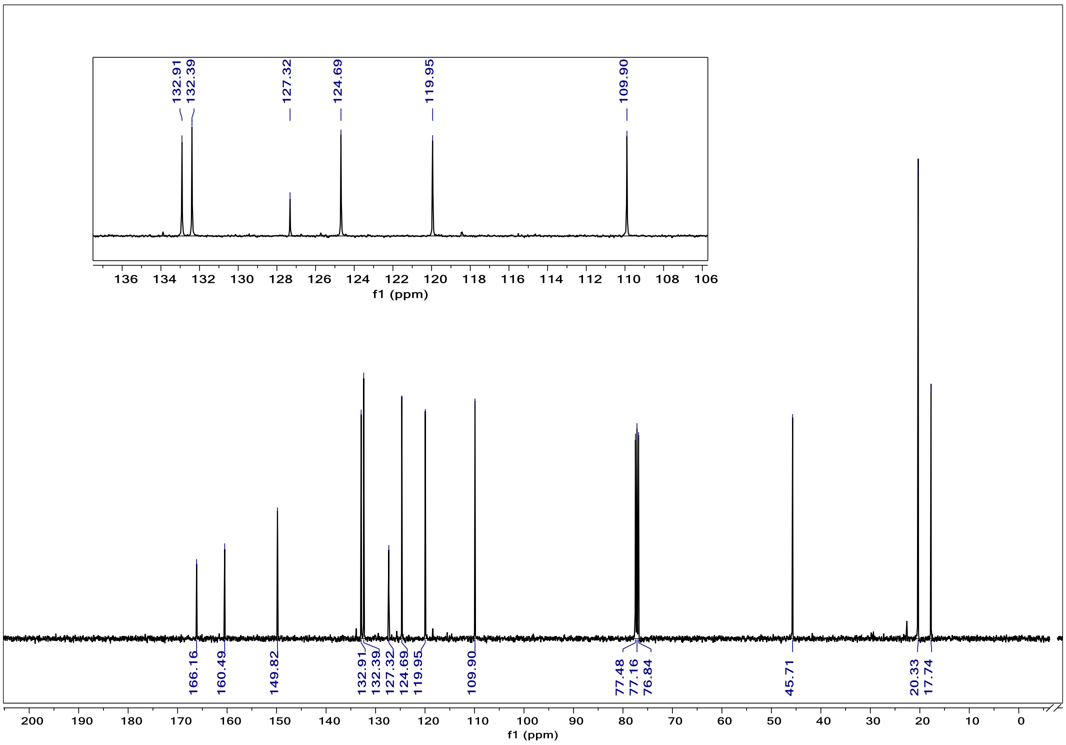

^{1}H NMR spectrum of **2d**

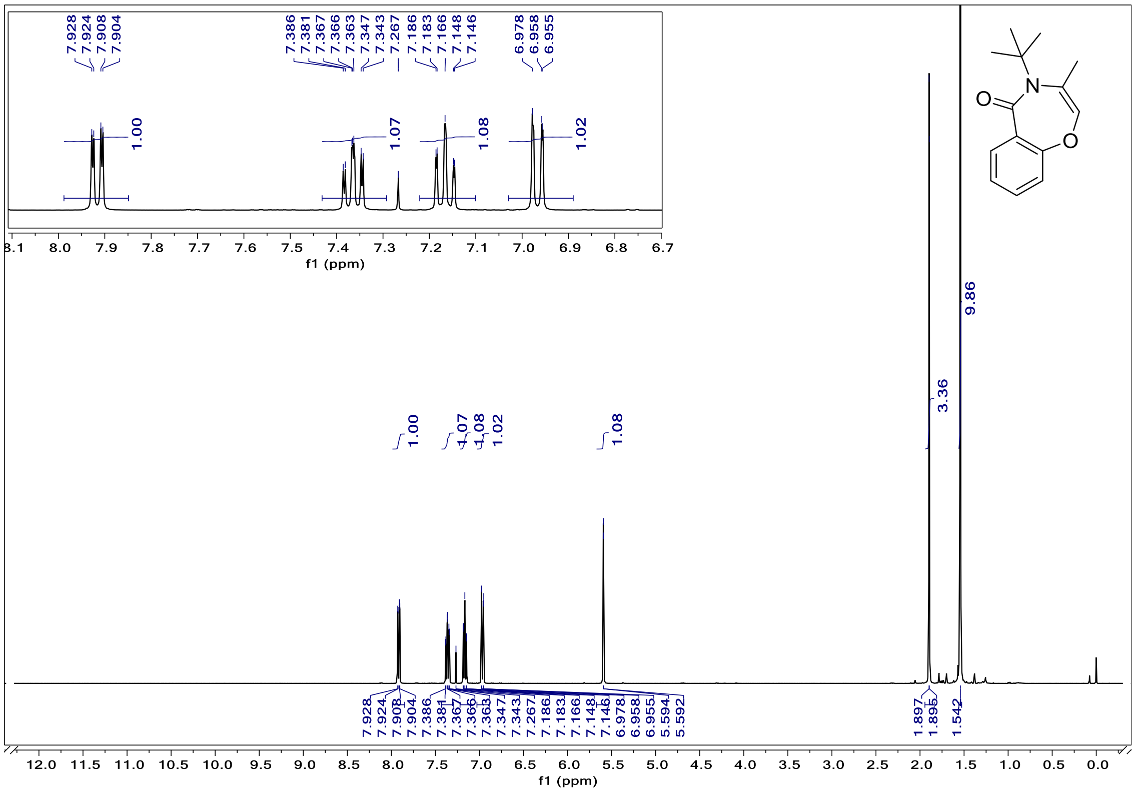

^{13}C NMR spectrum of **2d**

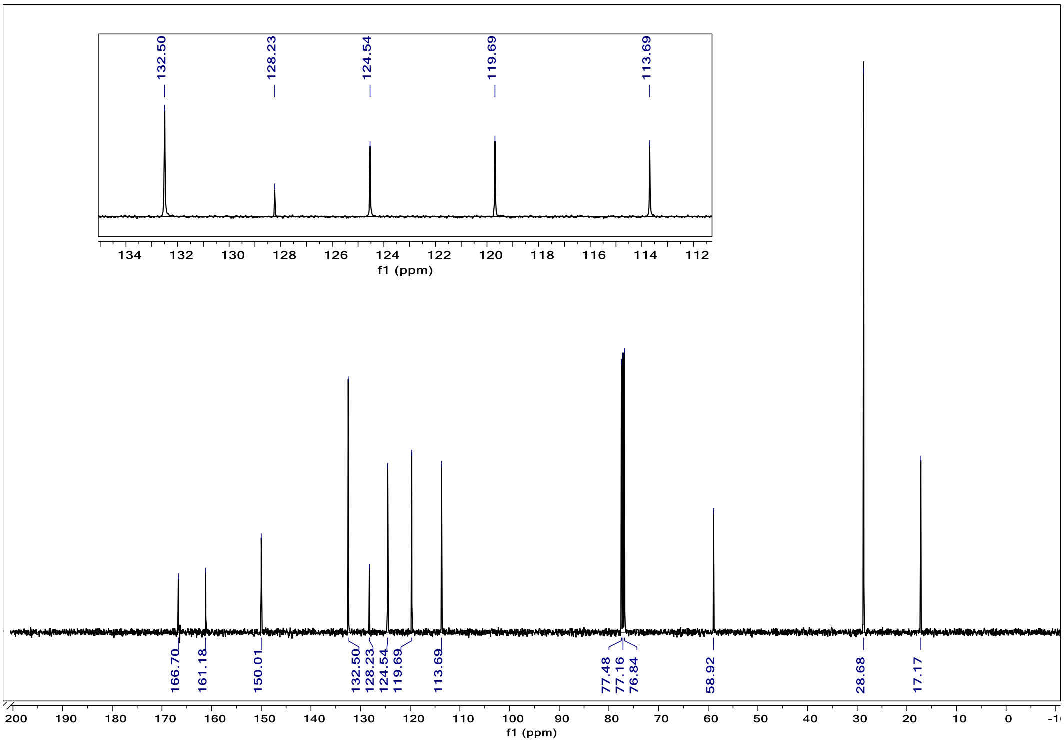

^{1}H NMR spectrum of **2e**

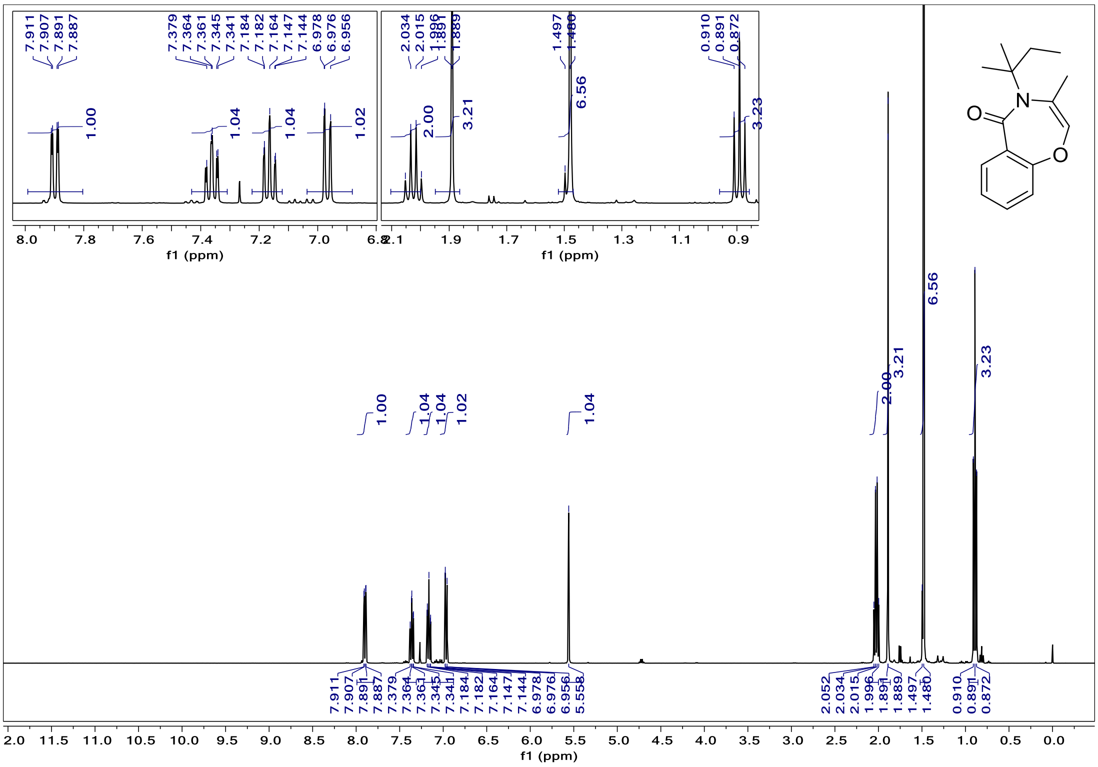

^{13}C NMR spectrum of **2e**

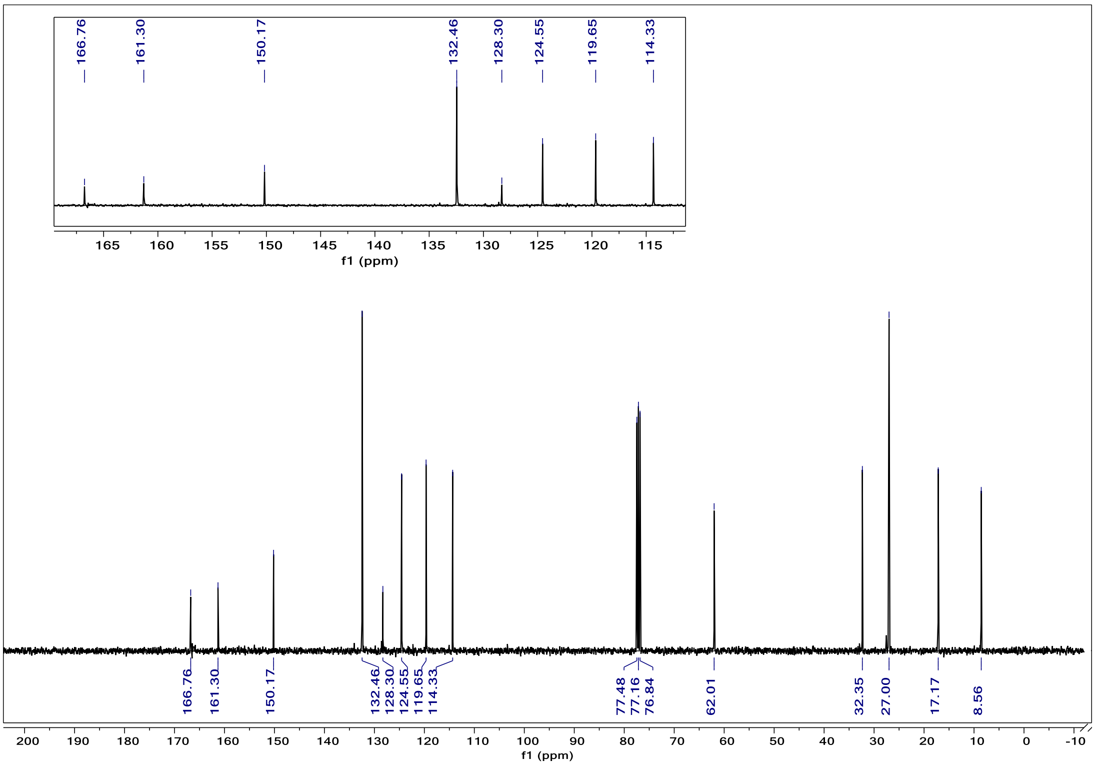

^{1}H NMR spectrum of **2f**

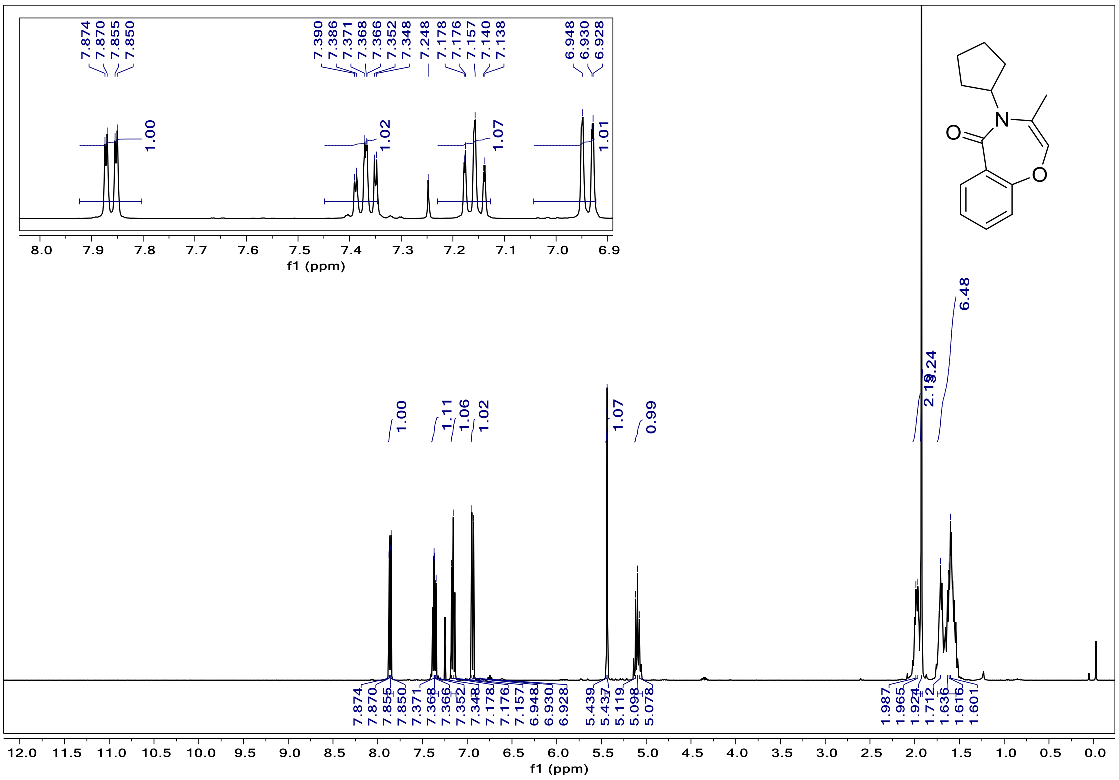

^{13}C NMR spectrum of **2f**

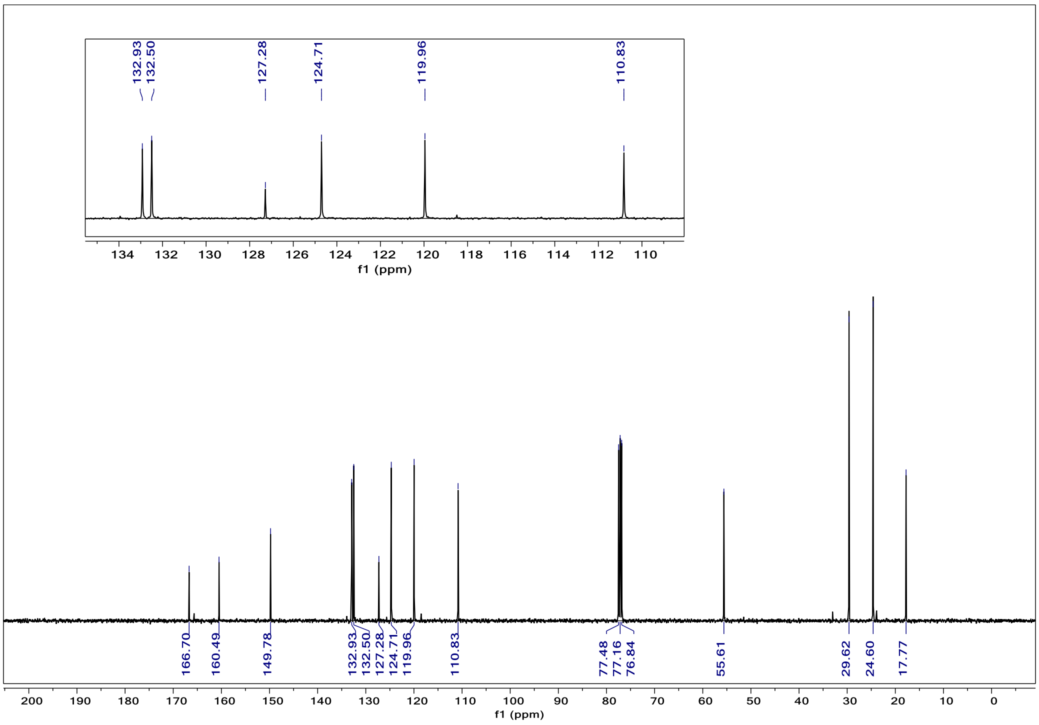

^{1}H NMR spectrum of **2g**

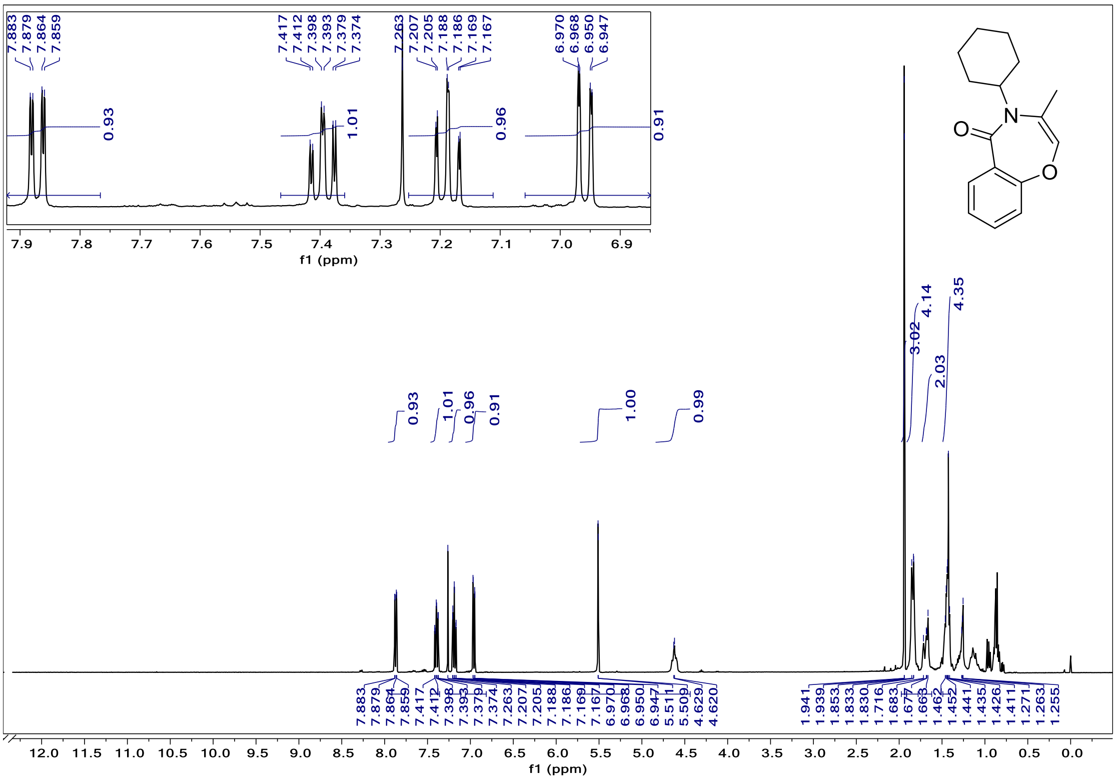

^{13}C NMR spectrum of **2g**

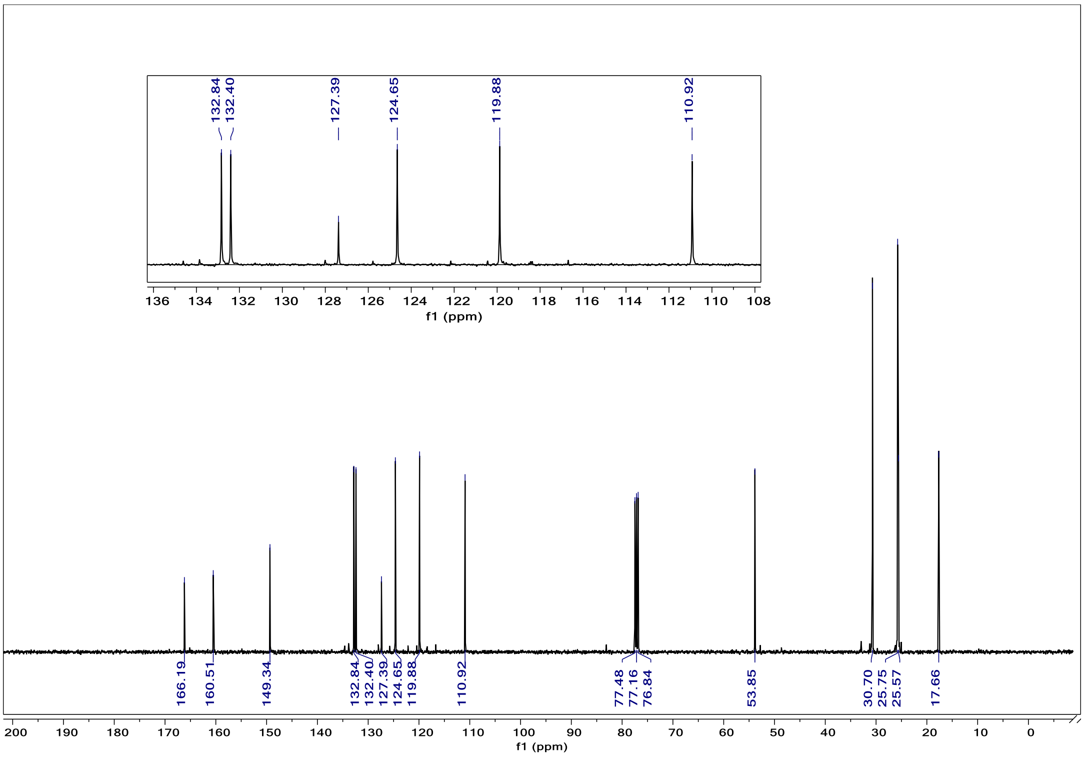

^{1}H NMR spectrum of **2h**

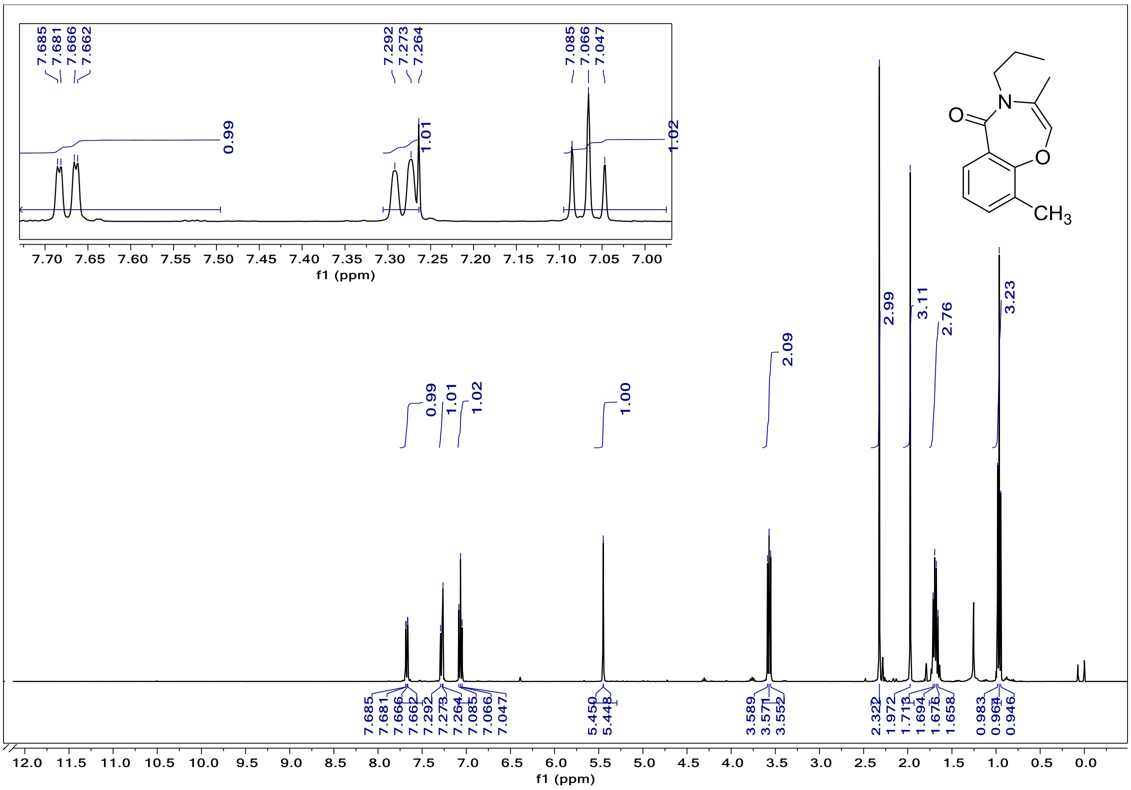

^{13}C NMR spectrum of **2h**

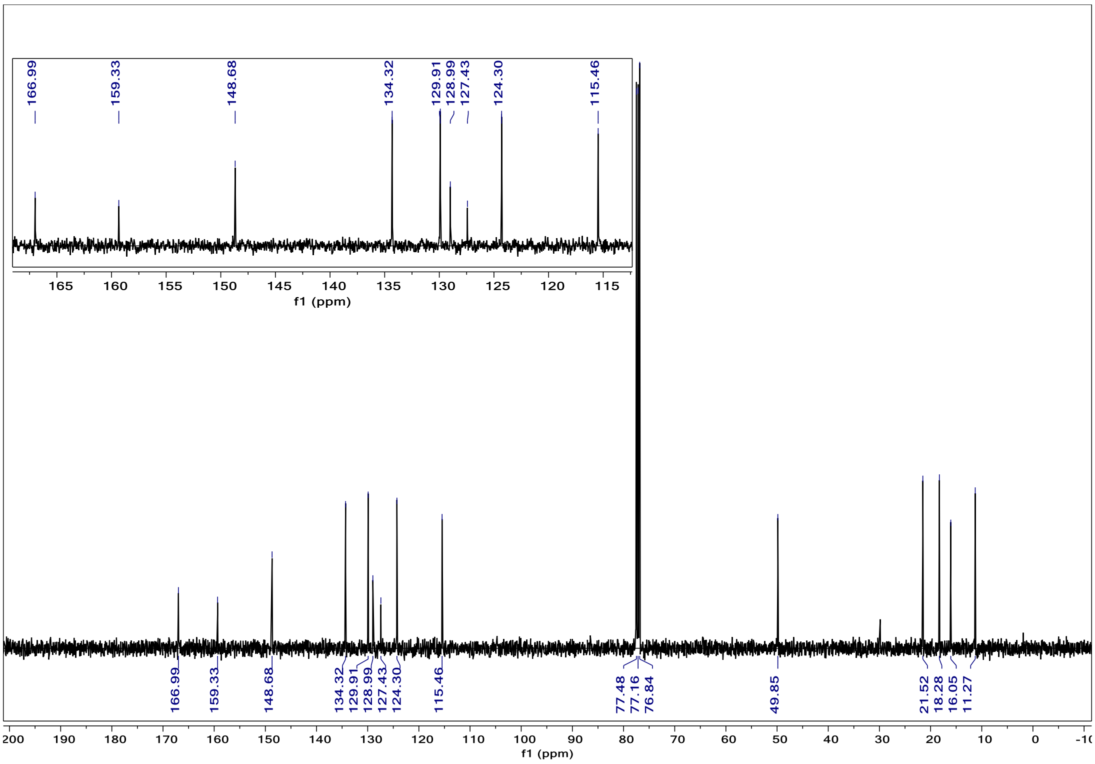

^{1}H NMR spectrum of **2i**

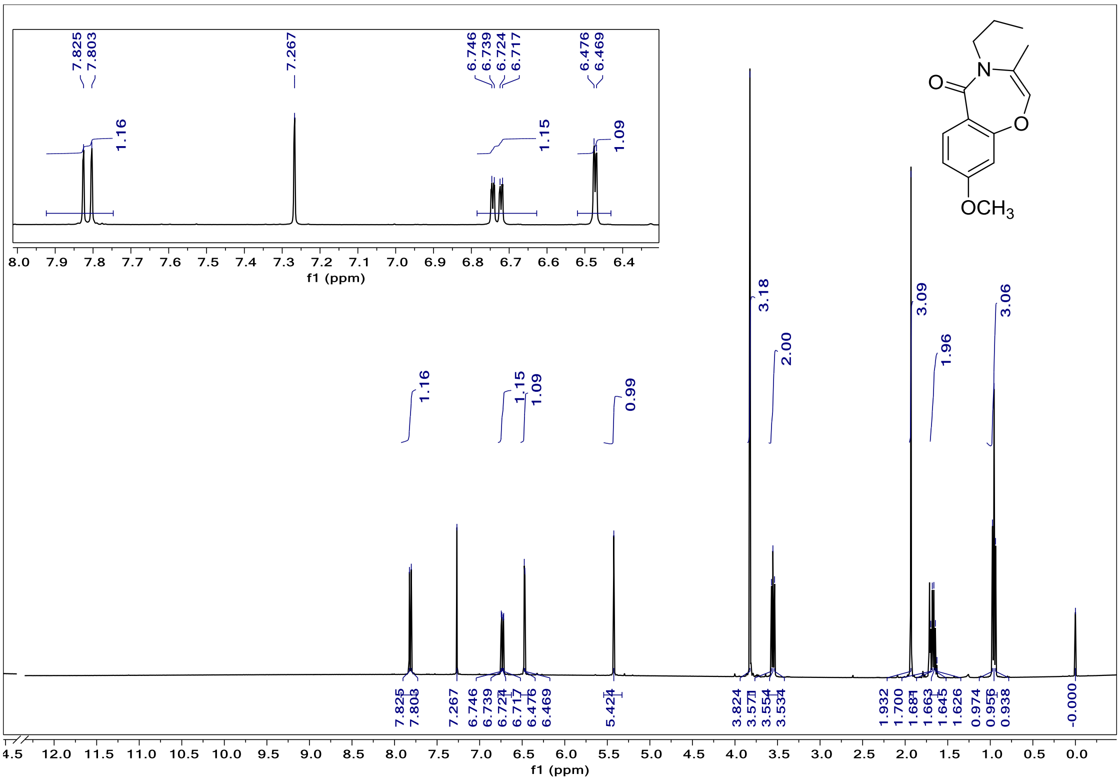

^{13}C NMR spectrum of **2i**

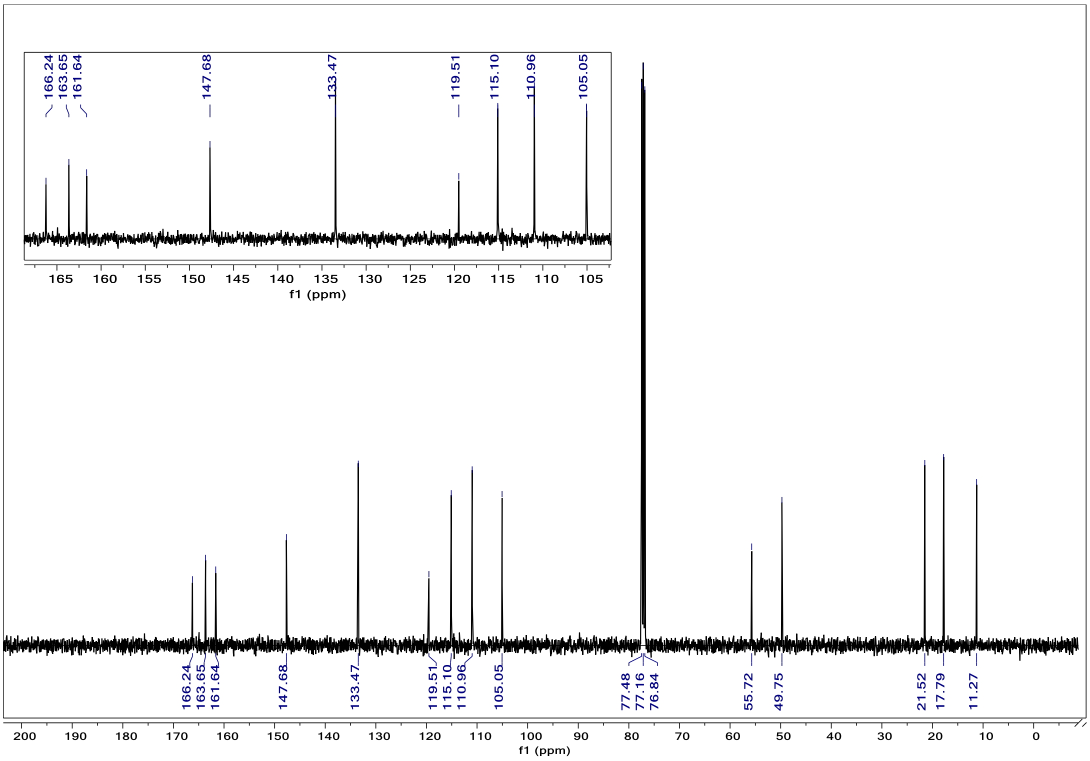

^{1}H NMR spectrum of **2j**

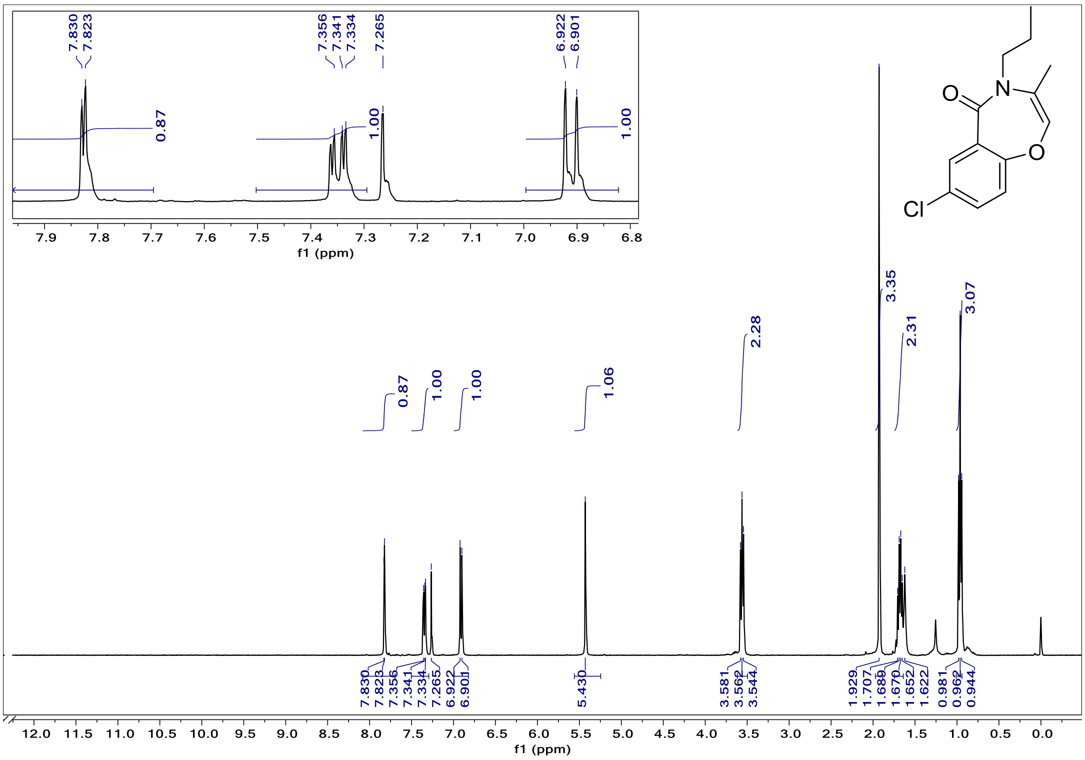

^{13}C NMR spectrum of **2j**

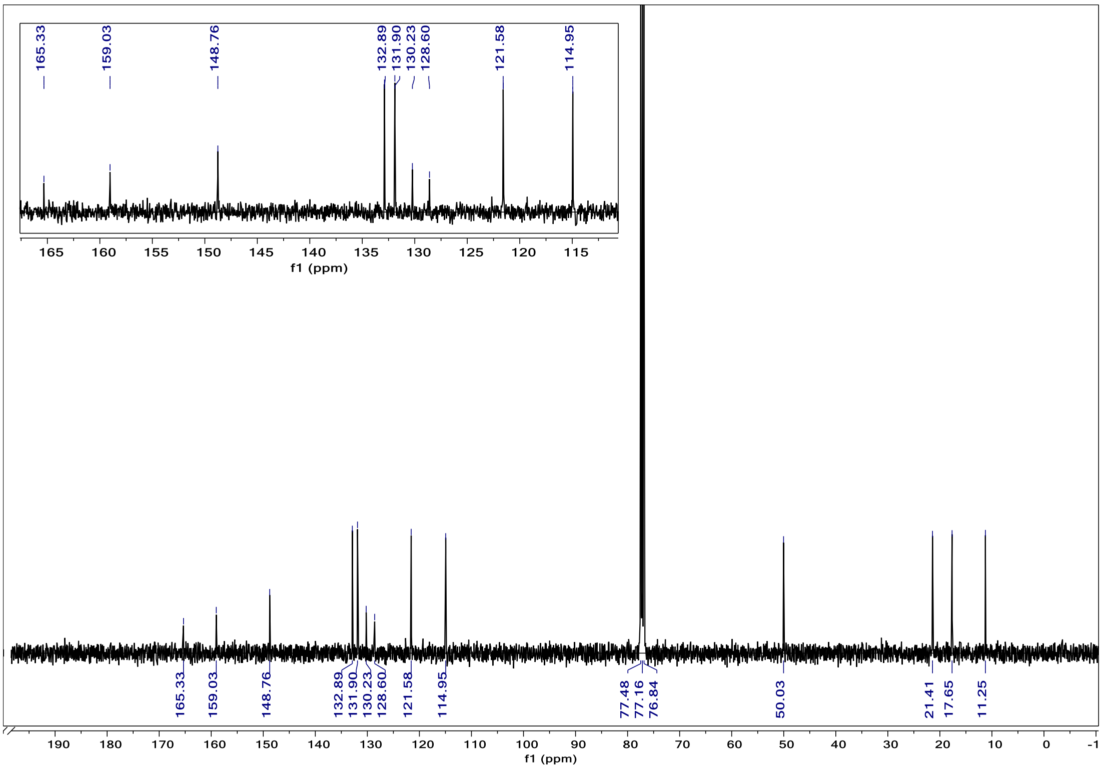

^{1}H NMR spectrum of **2k**

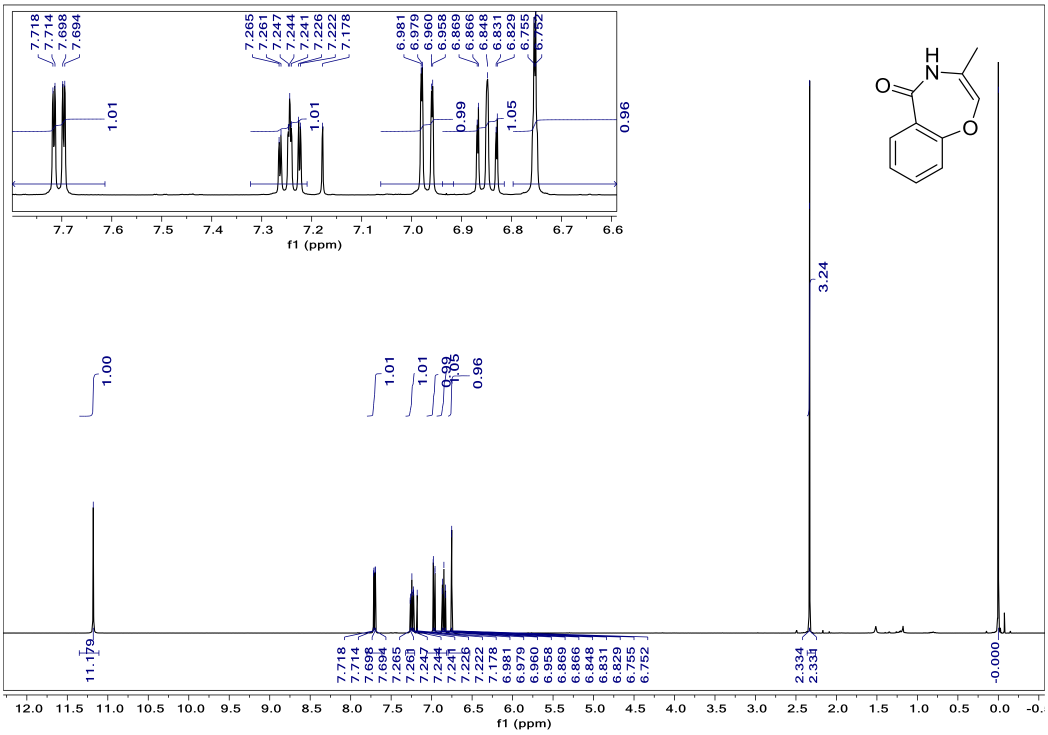

^{13}C NMR spectrum of **2k**

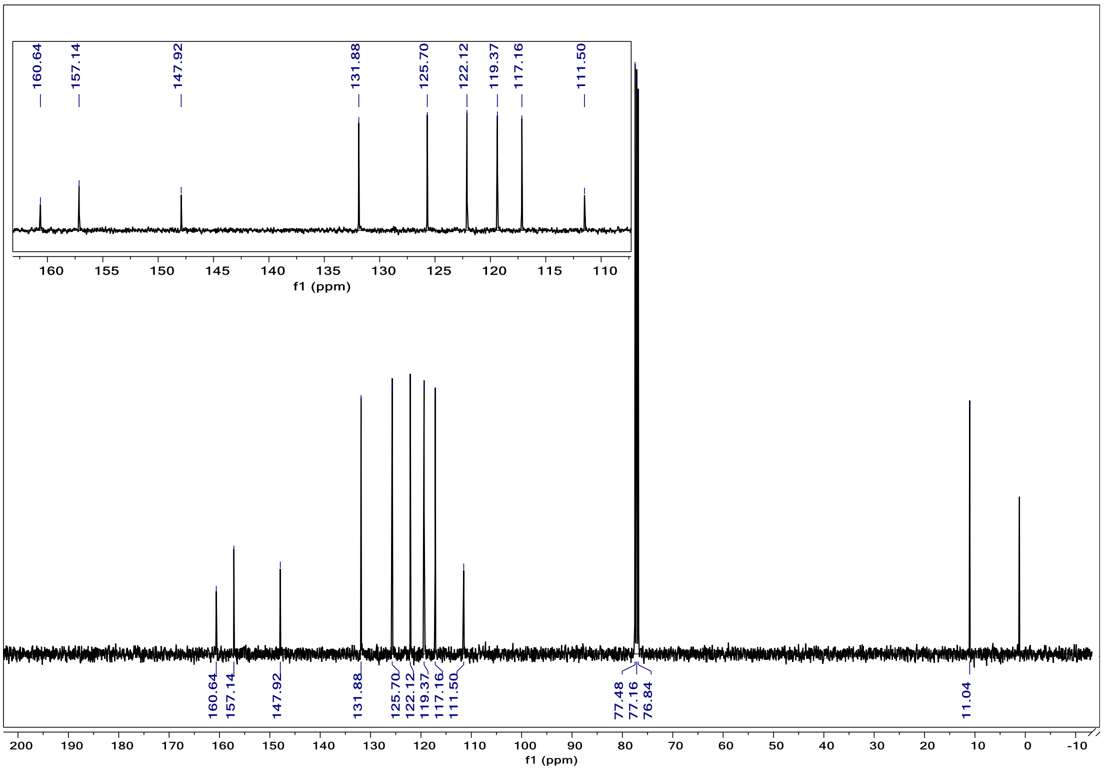

4. Copies of NMR spectra of 3

^{1}H NMR spectrum of **3a**

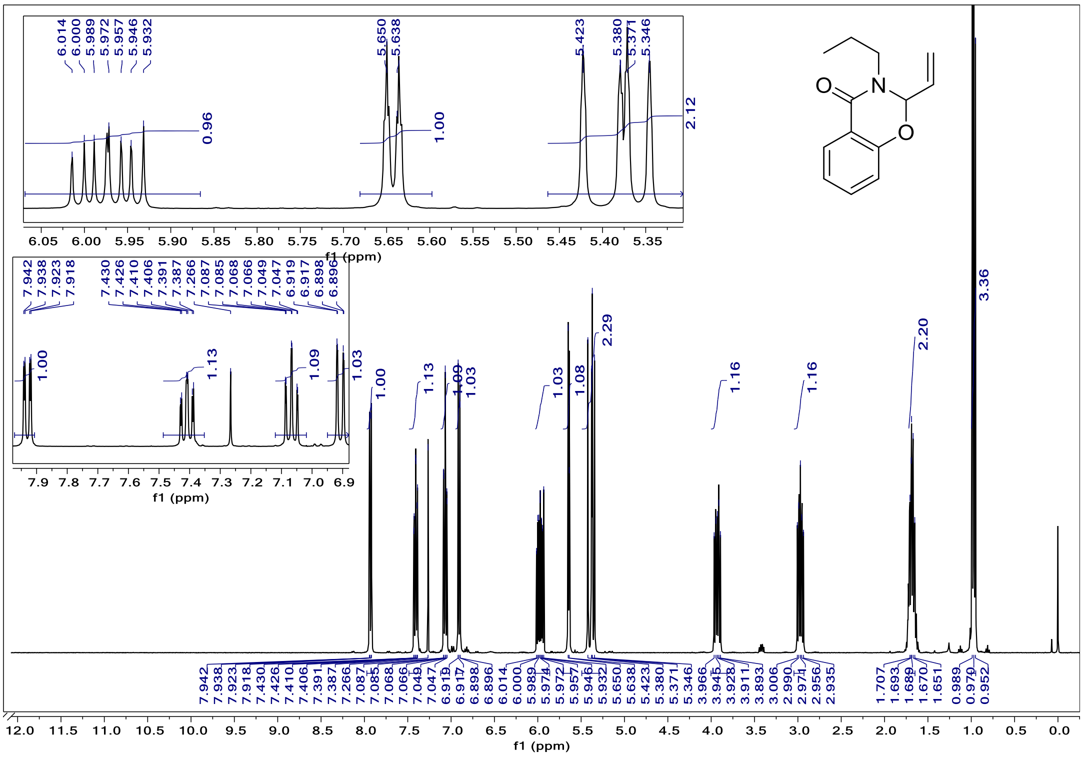

^{13}C NMR spectrum of **3a**

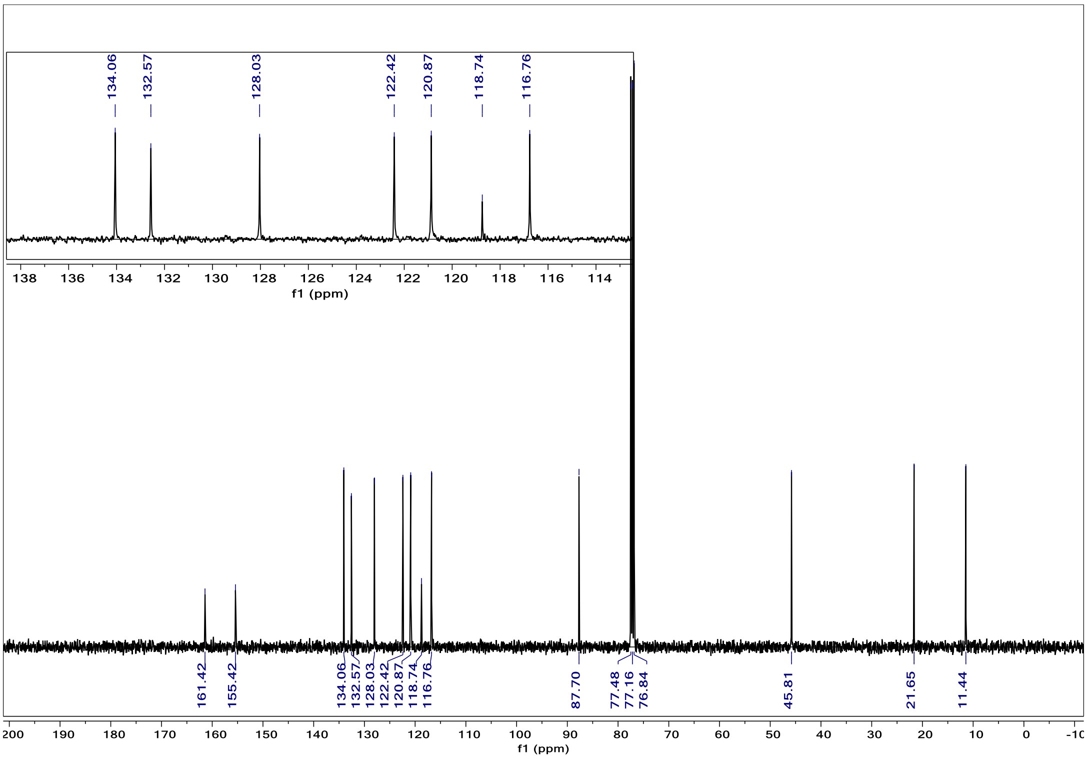

^{1}H NMR spectrum of **3b**

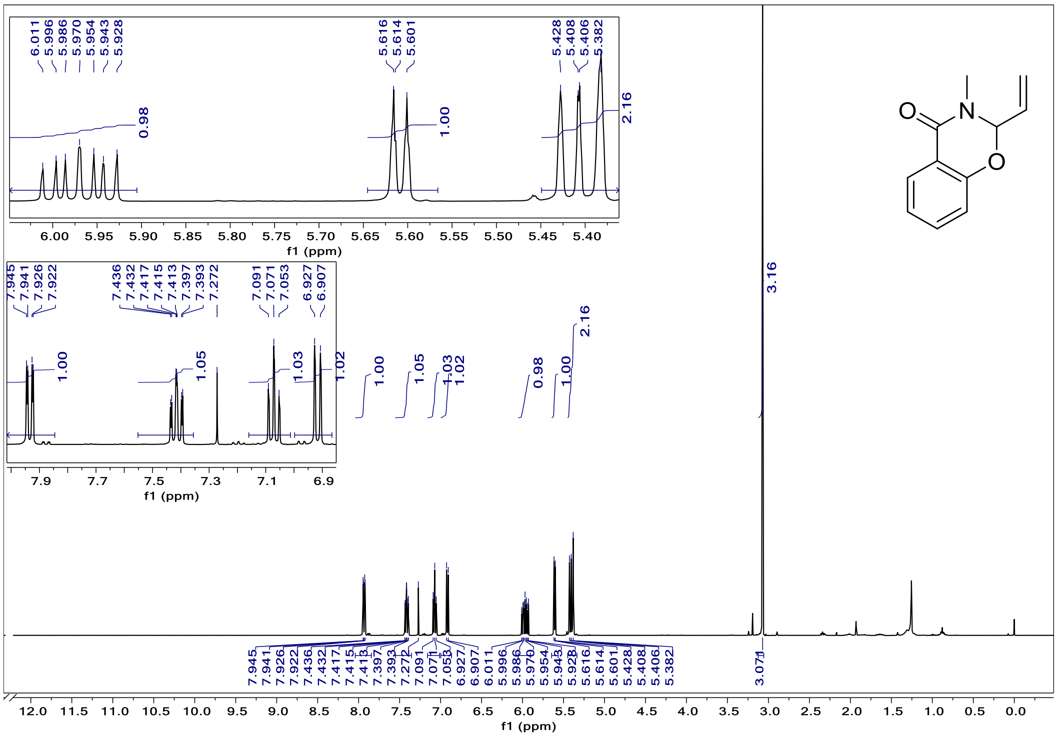

^{13}C NMR spectrum of **3b**

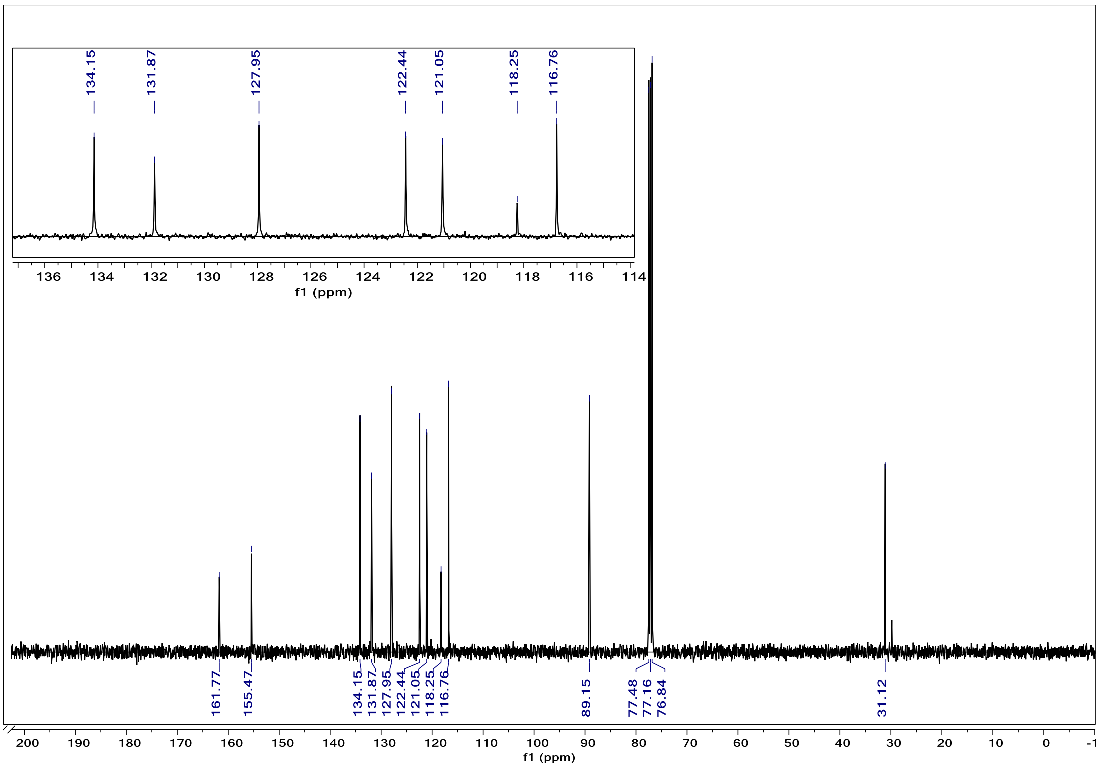

^{1}H NMR spectrum of **3c**

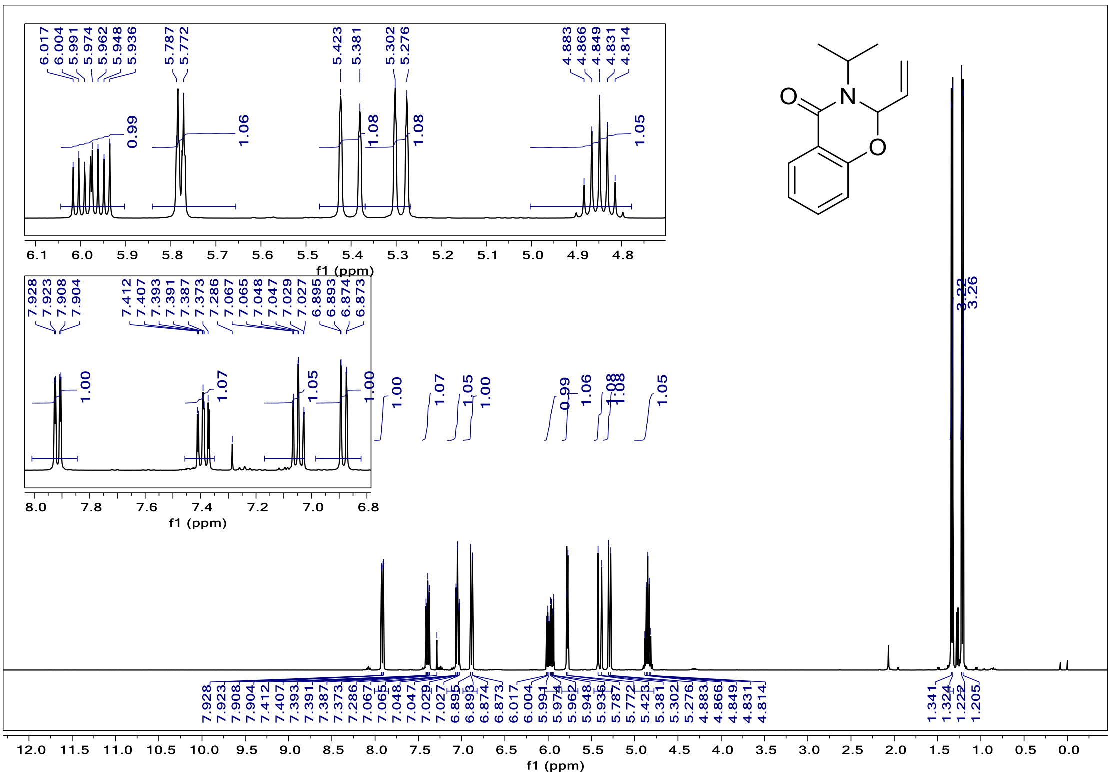

^{13}C NMR spectrum of **3c**

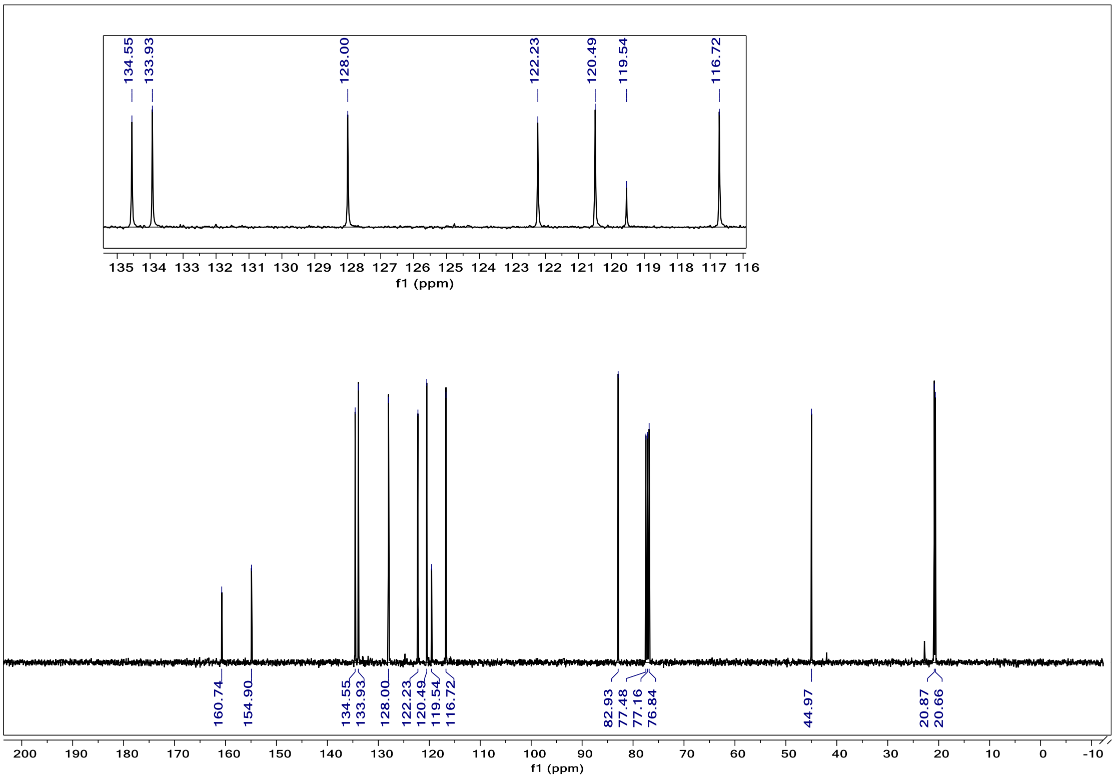

^{1}H NMR spectrum of **3d**

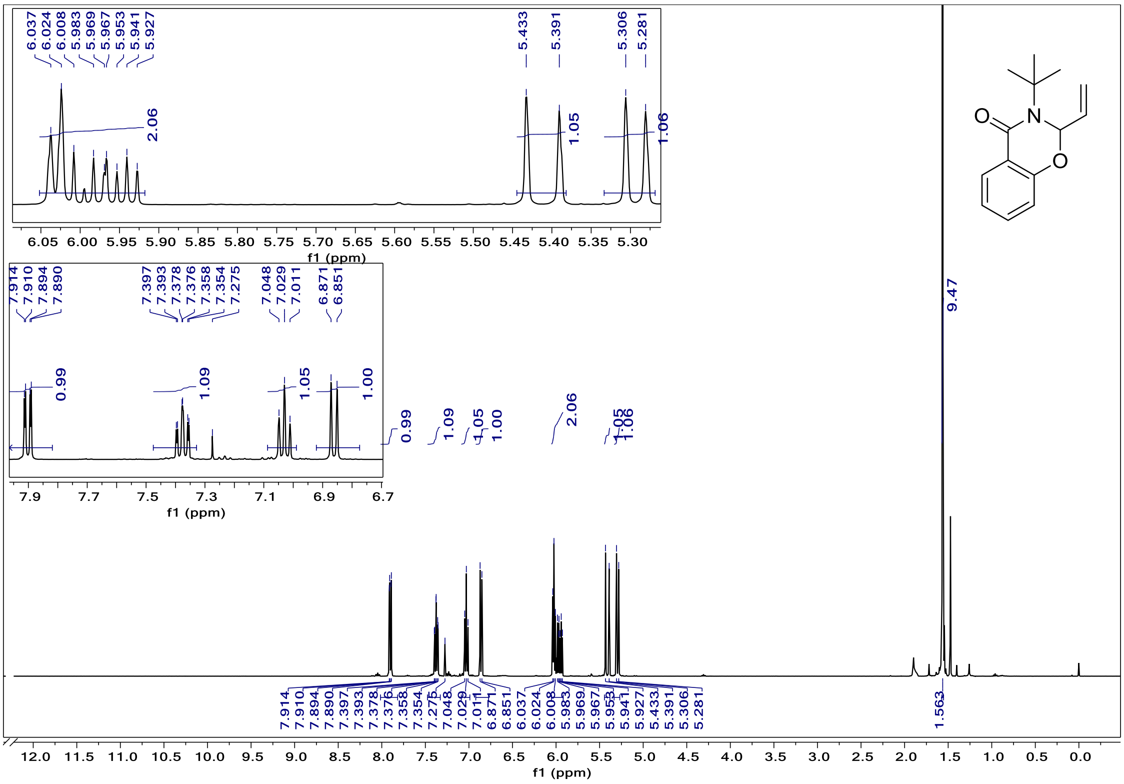

^{13}C NMR spectrum of **3d**

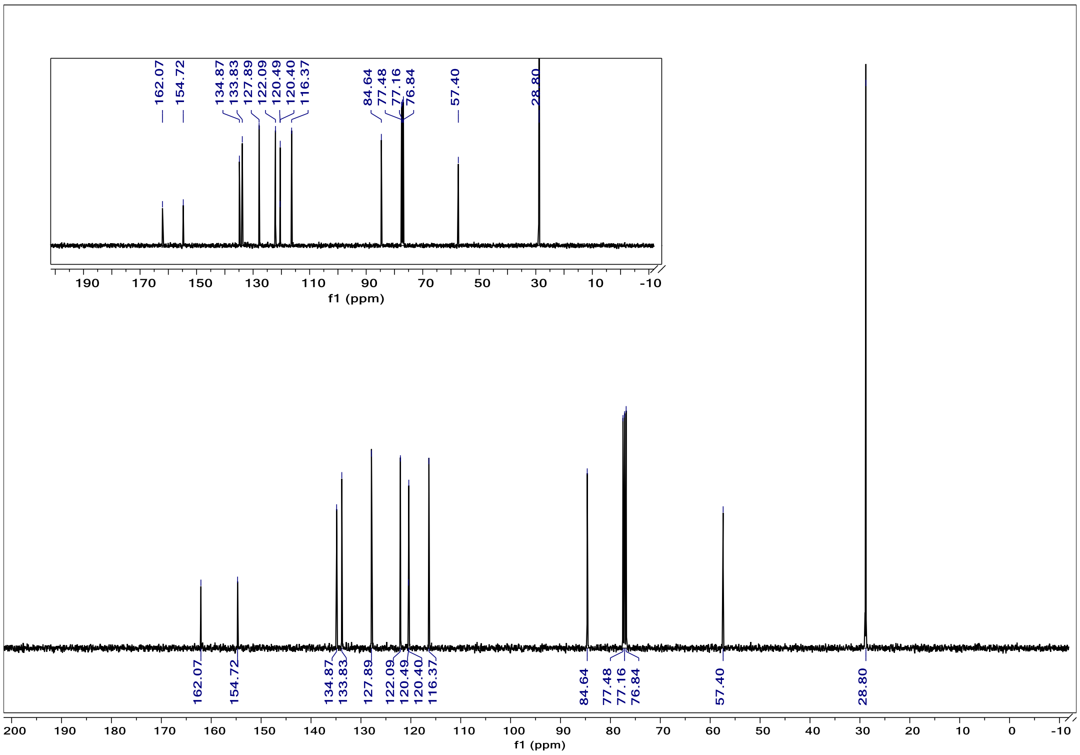

^{1}H NMR spectrum of **3g**

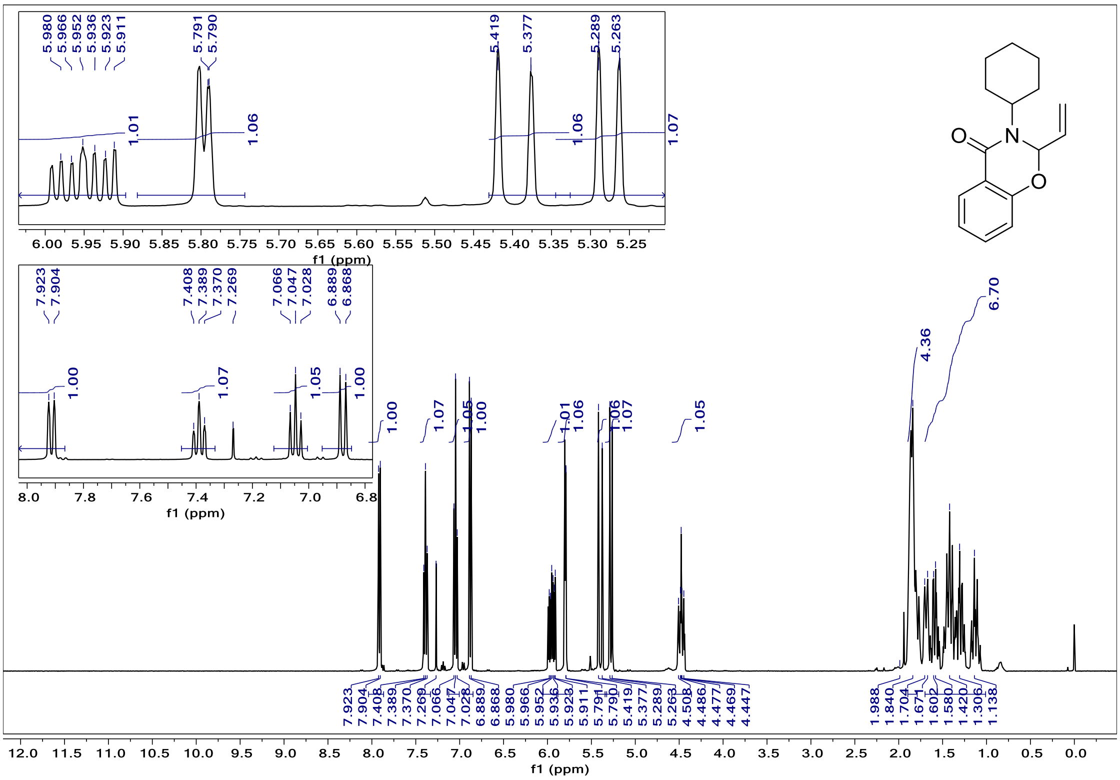

^{13}C NMR spectrum of **3g**

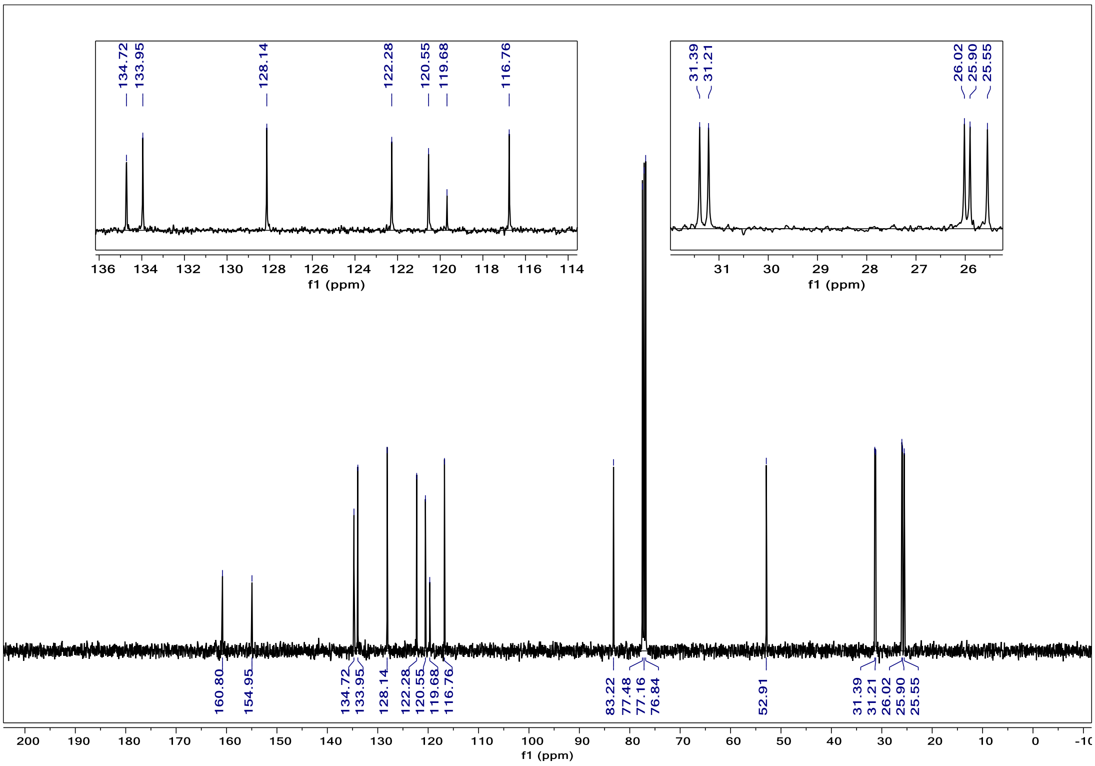

^{1}H NMR spectrum of **3h**

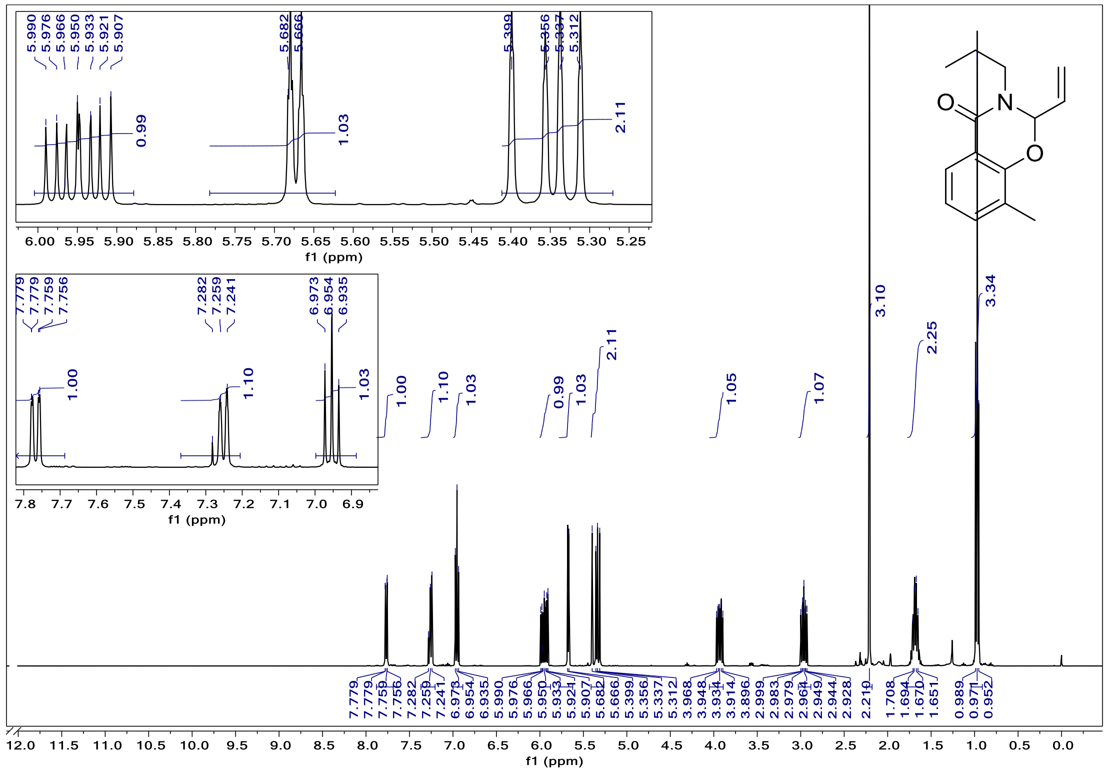

^{13}C NMR spectrum of **3h**

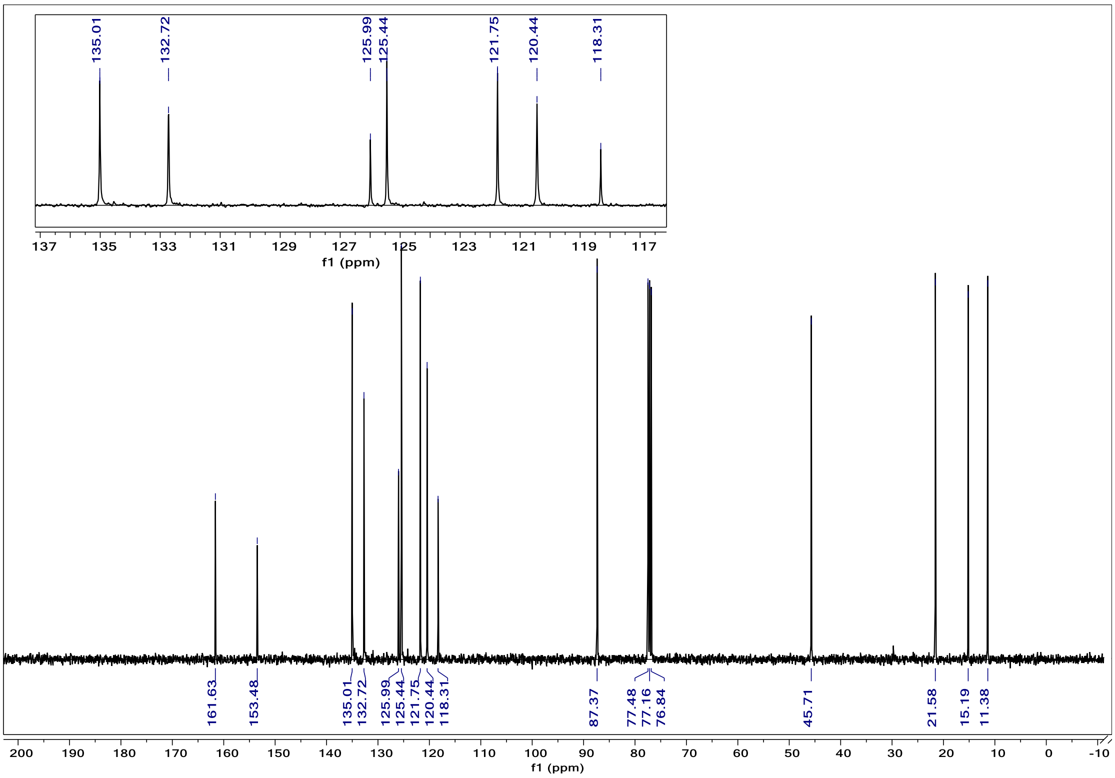

^{1}H NMR spectrum of **3i**

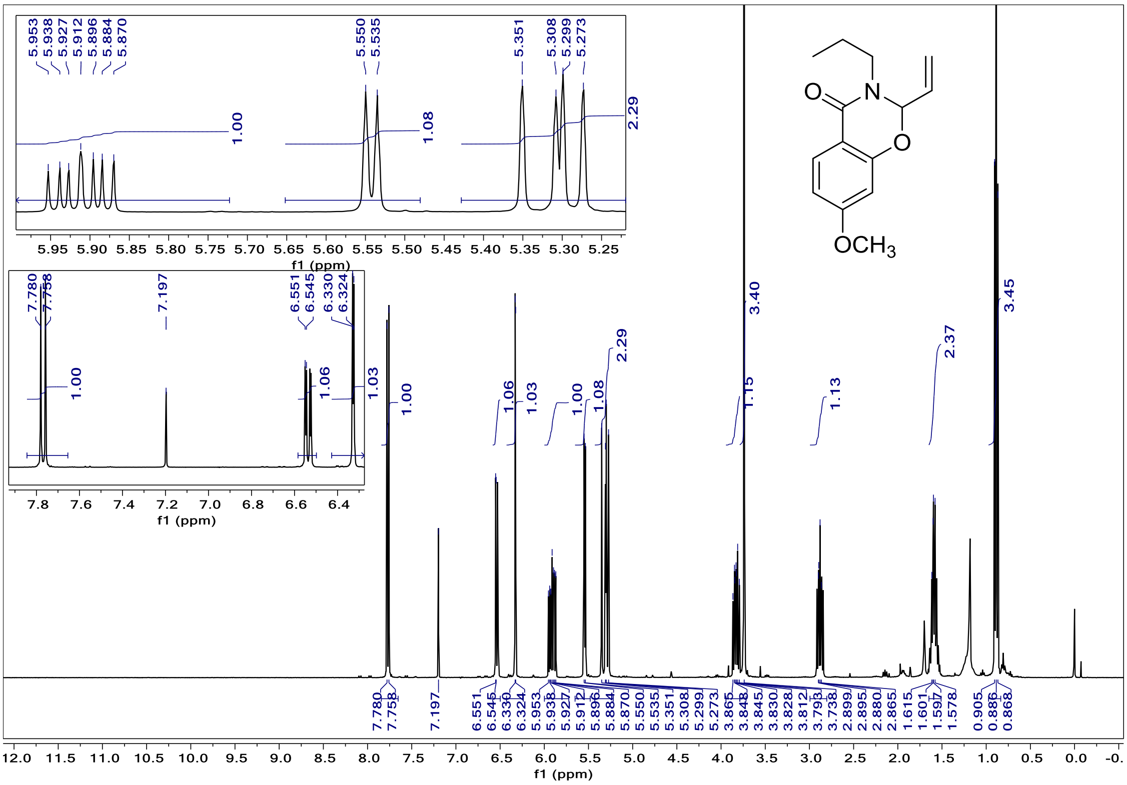

^{13}C NMR spectrum of **3i**

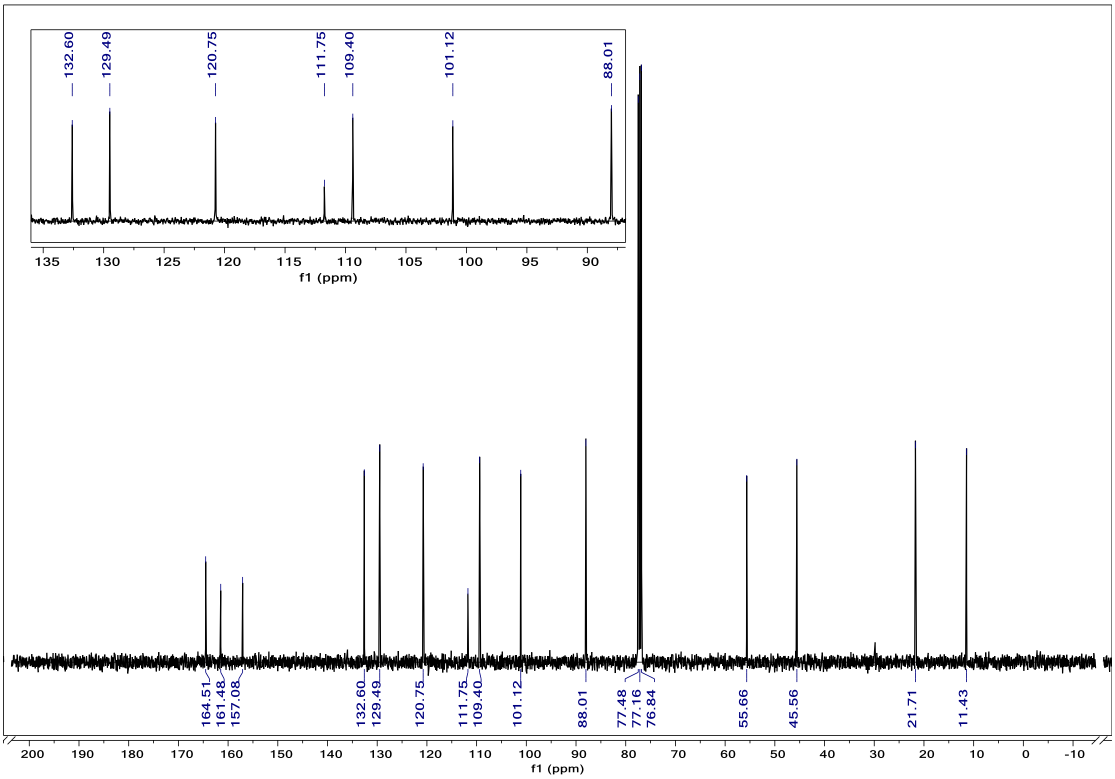

^{1}H NMR spectrum of **3j**

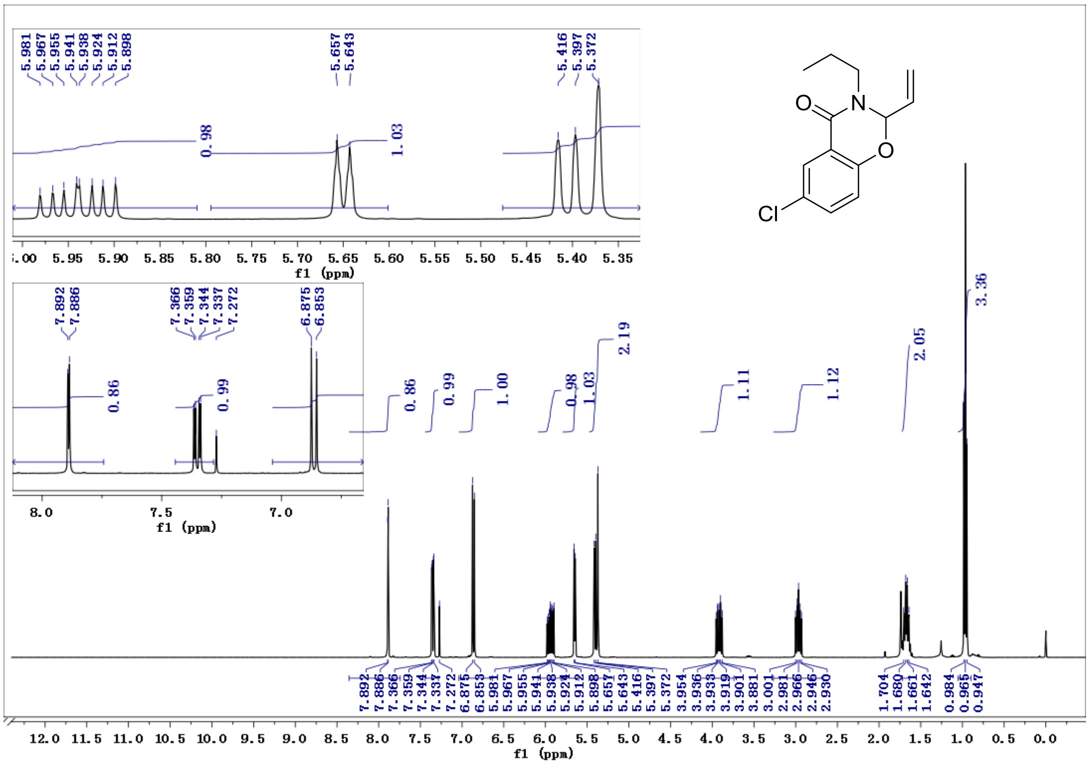

^{13}C NMR spectrum of **3j**

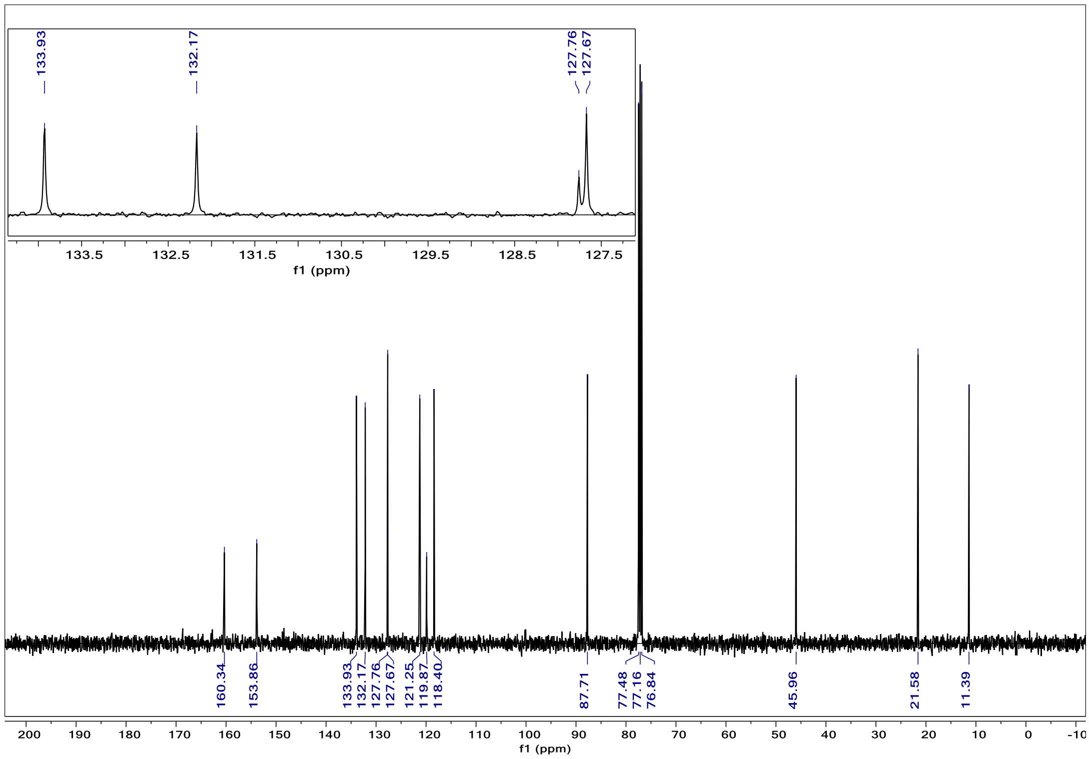

^{1}H NMR spectrum of **3l**

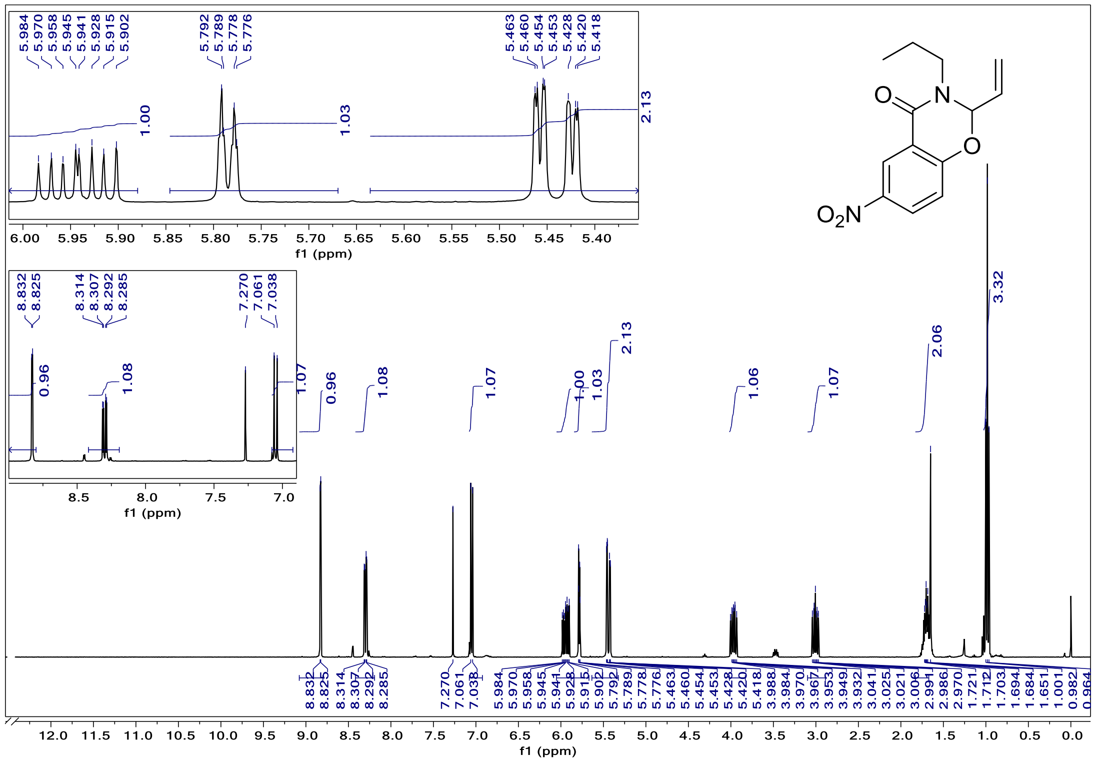

^{13}C NMR spectrum of **3l**

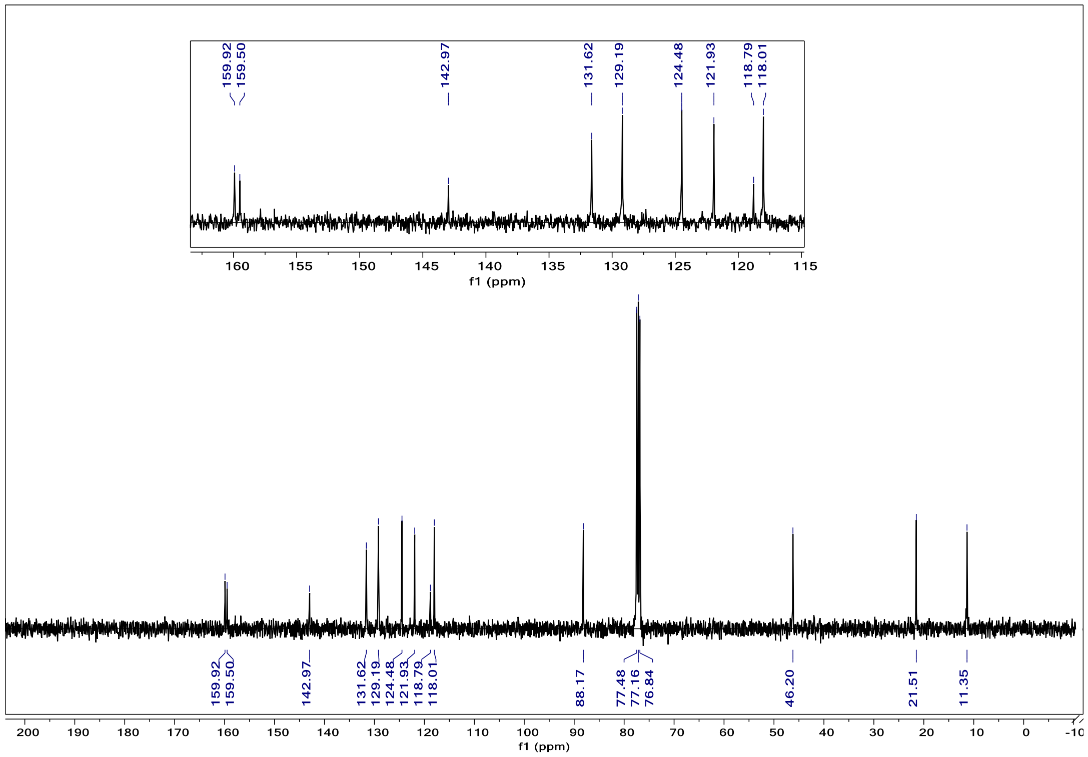

^{1}H NMR spectrum of **3m**

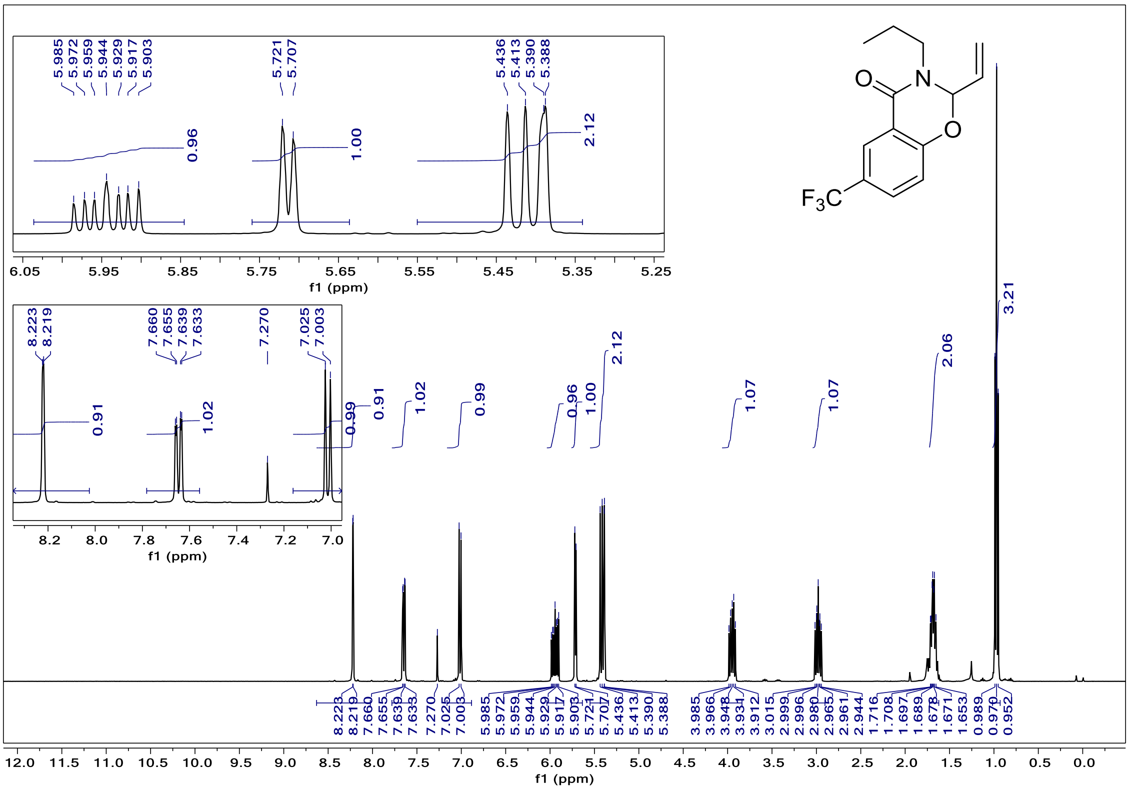

^{13}C NMR spectrum of **3m**

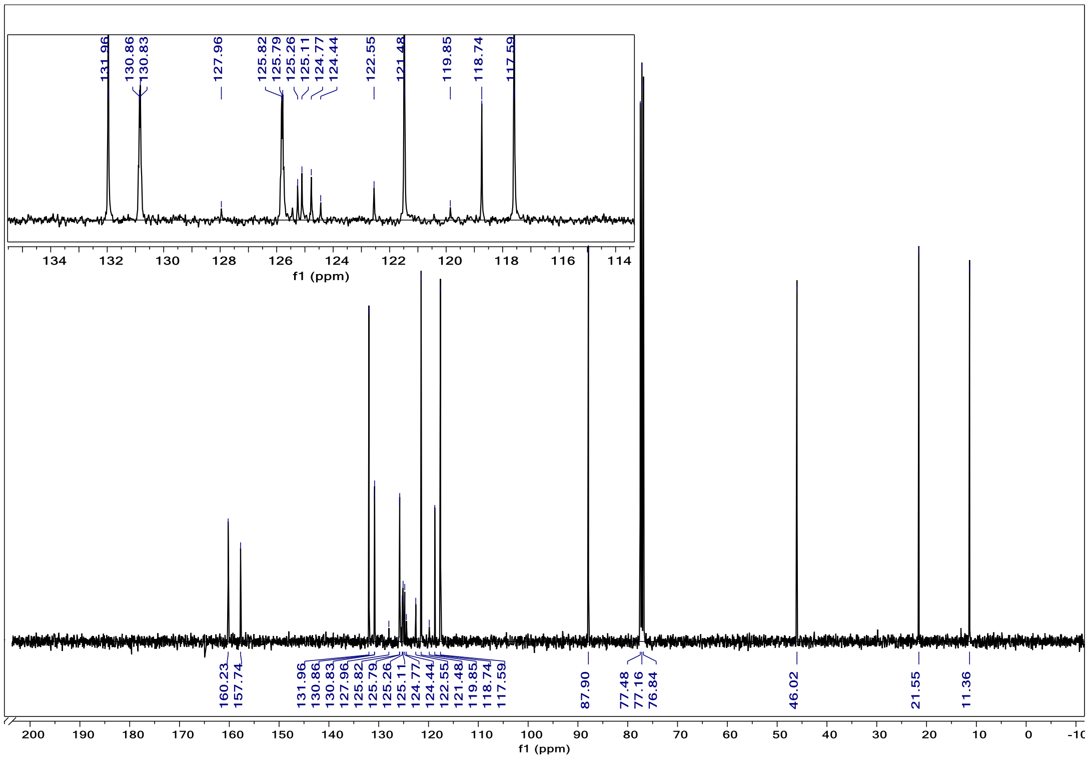

5. X-ray structural details of 2g and 3g

Crystal Structure of $C_{16}H_{19}NO_2$

The low temperature (173 ± 2°K) single-crystal X-ray experiments were performed on a SuperNova diffractometer with Cu K_α radiation. Unit cell was obtained and refined by 4943 reflections with 5.3° < θ < 71.2°. No decay was observed in data collection. Raw intensities were corrected for Lorentz and polarization effects, and for absorption by empirical method. Direct phase determination yielded the positions of all non-hydrogen atoms. All non-hydrogen atoms were subjected to anisotropic refinement. All hydrogen atoms were generated geometrically with C-H bonds of 0.93–0.98 Å according to criteria described in the SHELXTL manual (SHELXTL. Structure Determination Programs, Version 5.10, Bruker AXS Inc., Madison, WI, USA, 1997). They were included in the refinement with U_{iso}(H) = 1.2U_{eq} or 1.5U_{eq} (for methyl C) of their parent atoms. The final full-matric least-square refinement on F^2 converged with $R1$ = 0.0783 and $wR2$ = 0.1804 for 4525 observed reflections [I ≥ 2σ(I)]. The final difference electron density map shows no features. Details of crystal parameters, data collection and structure refinement are given in Table S2-1.

Data collection was controlled by CrysAlisPro, Agilent Technologies, Version 1.171.36.32 (Oxford Diffraction Ltd., Abingdon, Oxfordshire, OX, UK, 2013). Computations were performed using the SHELXTL NT ver. 5.10 program package (Bruker AXS Inc., Madison, WI, USA) on an IBM PC 586 computer. Analytic expressions of atomic scattering factors were employed, and anomalous dispersion corrections were incorporated [2,3]. Crystal drawings were produced with XP (SHELXTL. Structure Determination Programs, Version 5.10, Bruker AXS Inc., Madison, WI, USA).

Table S2-1. Details of Data Collection, Processing and Structure Refinement.

Sample code	**2g**
Molecular formula	$C_{16}H_{19}NO_2$
Molecular weight	257.32
Color and habit	colorless block
Crystal size	0.20 × 0.22 × 0.30 mm
Crystal system	monoclinic
Space group	*Pn* (No. 7)
Unit cell parameters	a = 6.5092(2) Å α = 90.00°
	b = 6.5112(2) Å β = 92.429(3)°
	c = 32.8651(9) Å γ = 90.00°
	V = 1391.66(7) Å^3 Z = 4 F(000) = 552
Density (calcd)	1.228 g/cm^3
Diffractometer	SuperNova, Dual, Cu at home/near, AtlasS2
Radiation	Cu K$_\alpha$, λ = 1.54178 Å
Temperature	173±2K
Scan type	ω-scan
Data collection range	$-7 < h < 7$, $-7 < k < 5$, $-38 < l < 40$; θ_{max} = 71.3°
Reflections measured	Total: 9158 Unique (n): 4618 Observed [I ≥ 2σ(I)]: 4525
Absorption coefficient	0.641 mm^{-1}
Minimum and maximum transmission	0.837, 1.000
No. of variables, *p*	345
Weighting scheme	$w = \dfrac{1}{\sigma^2(F_o^2)+(0.01P)^2+4.5P}$ $P = (F_o^2 + 2F_c^2)/3$
$R1 = \dfrac{\Sigma\|\|F_o\|-\|F_c\|\|}{\Sigma\|F_o\|}$ (for all reflections)	0.0792 0.0783 (for observed data)
$wR2 = \sqrt{\dfrac{\Sigma[w(F_o^2-F_c^2)^2]}{\Sigma w(F_o^2)^2}}$ (for all reflections)	0.1808 0.1804 (for observed data)
Goof = S = $\sqrt{\dfrac{\Sigma[w(F_o^2-F_c^2)^2]}{n-p}}$	1.129
Largest and mean Δ/σ	0.000, 0.000
Residual extrema in final difference map	-0.368 to 0.465 e Å^{-3}

Table S2-2. Atomic coordinates and equivalent isotropic temperature factors* (Å^2).

Atoms	*x*	*y*	*z*	$U_{eq.}$
O(1)	1.1150(7)	0.1624(8)	0.43578(16)	0.0316(12)
O(2)	0.7661(7)	0.1040(7)	0.33275(14)	0.0263(10)
N(1)	0.7816(8)	0.2226(8)	0.41622(17)	0.0223(11)
C(1)	0.9631(10)	0.1176(10)	0.41397(19)	0.0227(13)
C(2)	0.5970(10)	0.1751(10)	0.3935(2)	0.0239(14)
C(3)	0.5866(10)	0.1266(10)	0.3544(2)	0.0247(14)
C(4)	0.8799(11)	-0.0643(10)	0.3468(2)	0.0253(14)
C(5)	0.9045(12)	-0.2286(12)	0.3207(2)	0.0325(16)
C(6)	1.0272(14)	-0.3946(12)	0.3334(3)	0.0408(19)
C(7)	1.1219(13)	-0.3932(12)	0.3719(3)	0.0381(18)
C(8)	1.1002(12)	-0.2271(11)	0.3972(2)	0.0303(16)
C(9)	0.9729(10)	-0.0605(10)	0.3855(2)	0.0225(13)
C(10)	0.7728(11)	0.3854(10)	0.4478(2)	0.0243(14)
C(11)	0.7023(11)	0.2949(10)	0.4881(2)	0.0258(14)
C(12)	0.7140(13)	0.4597(11)	0.5211(2)	0.0325(16)
C(13)	0.5794(13)	0.6440(12)	0.5085(2)	0.0383(18)
C(14)	0.6406(13)	0.7286(11)	0.4674(2)	0.0348(17)
C(15)	0.6297(12)	0.5631(11)	0.4347(2)	0.0300(16)
C(16)	0.3920(13)	0.0875(14)	0.3299(2)	0.0385(19)
O(3)	1.1552(7)	0.5931(8)	0.12773(15)	0.0295(11)
O(4)	0.8499(7)	0.5264(7)	0.23087(14)	0.0252(10)
N(2)	0.8301(8)	0.6539(8)	0.14775(16)	0.0207(11)
C(17)	1.0142(10)	0.5483(10)	0.1495(2)	0.0230(14)
C(18)	0.6563(10)	0.6054(10)	0.1701(2)	0.0238(14)
C(19)	0.6625(10)	0.5531(10)	0.2092(2)	0.0230(13)
C(20)	0.9587(10)	0.3587(10)	0.2159(2)	0.0226(13)
C(21)	0.9862(11)	0.1917(11)	0.2413(2)	0.0290(15)
C(22)	1.1033(12)	0.0287(11)	0.2277(2)	0.0326(16)
C(23)	1.1874(12)	0.0330(11)	0.1896(2)	0.0318(16)
C(24)	1.1570(10)	0.2046(11)	0.1648(2)	0.0270(15)
C(25)	1.0335(10)	0.3683(10)	0.1777(2)	0.0221(13)
C(26)	0.8070(10)	0.8154(10)	0.1160(2)	0.0227(13)
C(27)	0.7220(11)	0.7248(10)	0.0756(2)	0.0253(14)
C(28)	0.7179(12)	0.8913(11)	0.0427(2)	0.0323(16)
C(29)	0.5897(14)	1.0748(13)	0.0551(2)	0.0393(19)
C(30)	0.6690(12)	1.1602(11)	0.0962(2)	0.0338(17)
C(31)	0.6715(12)	0.9930(10)	0.1290(2)	0.0280(15)
C(32)	0.4783(12)	0.5135(13)	0.2339(2)	0.0340(17)

*$U_{eq.}$ defined as one third of the trace of the orthogonalized **U** tensor.

Table S2-3. Bond lengths (Å) and bond angles (º).

Molecule **I**		Molecule **II**	
O(1)-C(1)	1.232(8)	O(3)-C(17)	1.223(9)
O(2)-C(4)	1.390(8)	O(4)-C(19)	1.397(8)
O(2)-C(3)	1.401(8)	O(4)-C(20)	1.402(8)
N(1)-C(1)	1.370(9)	N(2)-C(17)	1.380(8)
N(1)-C(2)	1.421(8)	N(2)-C(18)	1.411(9)
N(1)-C(10)	1.485(8)	N(2)-C(26)	1.486(8)
C(1)-C(9)	1.493(9)	C(17)-C(25)	1.497(9)
C(2)-C(3)	1.325(10)	C(18)-C(19)	1.327(10)
C(3)-C(16)	1.494(10)	C(19)-C(32)	1.499(9)
C(4)-C(5)	1.383(10)	C(20)-C(25)	1.367(9)
C(4)-C(9)	1.385(9)	C(20)-C(21)	1.378(9)
C(5)-C(6)	1.397(12)	C(21)-C(22)	1.392(11)
C(6)-C(7)	1.383(12)	C(22)-C(23)	1.386(11)
C(7)-C(8)	1.375(11)	C(23)-C(24)	1.392(10)
C(8)-C(9)	1.408(10)	C(24)-C(25)	1.411(9)
C(10)-C(15)	1.535(9)	C(26)-C(31)	1.527(9)
C(10)-C(11)	1.537(10)	C(26)-C(27)	1.532(9)
C(11)-C(12)	1.526(9)	C(27)-C(28)	1.533(9)
C(12)-C(13)	1.532(11)	C(28)-C(29)	1.523(11)
C(13)-C(14)	1.526(11)	C(29)-C(30)	1.530(11)
C(14)-C(15)	1.522(10)	C(30)-C(31)	1.533(10)
C(4)-O(2)-C(3)	111.1(5)	C(19)-O(4)-C(20)	111.1(5)
C(1)-N(1)-C(2)	125.1(5)	C(17)-N(2)-C(18)	125.5(5)
C(1)-N(1)-C(10)	116.9(5)	C(17)-N(2)-C(26)	116.3(5)
C(2)-N(1)-C(10)	117.7(5)	C(18)-N(2)-C(26)	117.7(5)
O(1)-C(1)-N(1)	121.5(6)	O(3)-C(17)-N(2)	121.9(6)
O(1)-C(1)-C(9)	119.7(6)	O(3)-C(17)-C(25)	120.3(6)
N(1)-C(1)-C(9)	118.8(6)	N(2)-C(17)-C(25)	117.7(6)
C(3)-C(2)-N(1)	124.7(6)	C(19)-C(18)-N(2)	124.7(6)
C(2)-C(3)-O(2)	120.6(6)	C(18)-C(19)-O(4)	121.1(6)
C(2)-C(3)-C(16)	124.8(7)	C(18)-C(19)-C(32)	125.2(6)
O(2)-C(3)-C(16)	114.6(6)	O(4)-C(19)-C(32)	113.7(6)
C(5)-C(4)-C(9)	121.6(7)	C(25)-C(20)-C(21)	123.4(6)
C(5)-C(4)-O(2)	118.6(6)	C(25)-C(20)-O(4)	119.4(6)
C(9)-C(4)-O(2)	119.7(6)	C(21)-C(20)-O(4)	117.2(6)
C(4)-C(5)-C(6)	119.6(7)	C(20)-C(21)-C(22)	117.8(7)
C(7)-C(6)-C(5)	119.6(7)	C(23)-C(22)-C(21)	121.2(7)
C(8)-C(7)-C(6)	120.3(7)	C(22)-C(23)-C(24)	119.5(7)
C(7)-C(8)-C(9)	121.0(7)	C(23)-C(24)-C(25)	119.9(7)

(Table S2-3. continued)

C(4)-C(9)-C(8)	117.7(6)	C(20)-C(25)-C(24)	118.1(6)
C(4)-C(9)-C(1)	124.1(6)	C(20)-C(25)-C(17)	125.5(6)
C(8)-C(9)-C(1)	117.8(6)	C(24)-C(25)-C(17)	116.1(6)
N(1)-C(10)-C(15)	112.6(5)	N(2)-C(26)-C(31)	112.4(5)
N(1)-C(10)-C(11)	110.5(5)	N(2)-C(26)-C(27)	111.0(5)
C(15)-C(10)-C(11)	109.3(6)	C(31)-C(26)-C(27)	110.1(6)
C(12)-C(11)-C(10)	109.6(6)	C(26)-C(27)-C(28)	109.5(5)
C(11)-C(12)-C(13)	110.3(6)	C(29)-C(28)-C(27)	111.0(6)
C(14)-C(13)-C(12)	110.7(7)	C(28)-C(29)-C(30)	110.7(6)
C(15)-C(14)-C(13)	111.3(6)	C(29)-C(30)-C(31)	110.9(6)
C(14)-C(15)-C(10)	109.1(6)	C(26)-C(31)-C(30)	109.2(6)

Table S2-4. Anisotropic thermal parameters* (Å^2).

Atoms	U_{11}	U_{22}	U_{33}	U_{23}	U_{13}	U_{12}
O(1)	0.021(3)	0.038(3)	0.035(3)	-0.009(2)	-0.006(2)	0.005(2)
O(2)	0.025(2)	0.028(3)	0.026(2)	0.005(2)	0.0039(19)	0.000(2)
N(1)	0.020(3)	0.020(3)	0.026(3)	-0.005(2)	0.000(2)	0.001(2)
C(1)	0.025(3)	0.023(3)	0.021(3)	-0.003(3)	0.003(3)	-0.003(3)
C(2)	0.014(3)	0.023(3)	0.035(4)	-0.002(3)	0.000(3)	0.001(3)
C(3)	0.020(3)	0.024(3)	0.030(4)	0.002(3)	0.003(3)	-0.001(3)
C(4)	0.027(3)	0.021(3)	0.028(3)	0.004(3)	-0.003(3)	-0.006(3)
C(5)	0.035(4)	0.036(4)	0.027(4)	-0.007(3)	0.006(3)	-0.006(3)
C(6)	0.048(5)	0.029(4)	0.045(5)	-0.013(3)	-0.001(4)	0.004(4)
C(7)	0.040(5)	0.027(4)	0.047(5)	-0.002(3)	0.002(4)	0.011(3)
C(8)	0.033(4)	0.027(4)	0.031(4)	0.002(3)	-0.004(3)	0.005(3)
C(9)	0.019(3)	0.022(3)	0.027(3)	-0.002(3)	0.004(3)	-0.003(3)
C(10)	0.028(3)	0.019(3)	0.026(3)	-0.003(3)	0.000(3)	-0.001(3)
C(11)	0.029(4)	0.022(3)	0.026(3)	-0.001(3)	0.004(3)	0.001(3)
C(12)	0.042(4)	0.029(4)	0.026(4)	-0.003(3)	0.005(3)	0.006(3)
C(13)	0.043(5)	0.035(4)	0.038(4)	-0.011(3)	0.006(4)	0.006(4)
C(14)	0.043(5)	0.019(3)	0.043(4)	-0.003(3)	-0.001(4)	0.005(3)
C(15)	0.034(4)	0.024(4)	0.032(4)	-0.001(3)	-0.001(3)	0.008(3)
C(16)	0.036(4)	0.051(5)	0.028(4)	0.002(3)	-0.006(3)	-0.008(4)
O(3)	0.023(2)	0.034(3)	0.032(3)	0.006(2)	0.008(2)	0.002(2)
O(4)	0.027(2)	0.025(2)	0.023(2)	-0.0053(19)	0.0012(19)	0.000(2)
N(2)	0.020(3)	0.021(3)	0.022(3)	0.004(2)	0.003(2)	-0.002(2)
C(17)	0.021(3)	0.023(3)	0.025(3)	-0.002(3)	-0.003(3)	0.000(3)
C(18)	0.017(3)	0.020(3)	0.035(4)	-0.001(3)	0.002(3)	0.001(3)
C(19)	0.016(3)	0.023(3)	0.030(4)	-0.004(3)	0.003(3)	-0.005(3)
C(20)	0.020(3)	0.020(3)	0.028(3)	-0.003(3)	-0.002(3)	-0.002(3)
C(21)	0.033(4)	0.027(4)	0.027(3)	0.003(3)	0.002(3)	0.001(3)
C(22)	0.035(4)	0.025(4)	0.038(4)	0.005(3)	0.000(3)	0.004(3)
C(23)	0.033(4)	0.026(4)	0.036(4)	0.000(3)	-0.001(3)	0.008(3)
C(24)	0.023(3)	0.029(4)	0.029(4)	-0.001(3)	0.002(3)	0.006(3)
C(25)	0.023(3)	0.017(3)	0.026(3)	-0.003(2)	-0.002(3)	0.002(3)
C(26)	0.026(3)	0.018(3)	0.024(3)	0.003(3)	0.001(3)	-0.001(3)
C(27)	0.027(4)	0.017(3)	0.031(4)	-0.001(3)	-0.002(3)	0.000(3)
C(28)	0.038(4)	0.032(4)	0.027(4)	0.003(3)	-0.002(3)	-0.002(3)
C(29)	0.045(5)	0.034(4)	0.039(4)	0.004(3)	-0.006(4)	0.007(4)
C(30)	0.037(4)	0.025(4)	0.040(4)	0.004(3)	0.001(3)	0.002(3)
C(31)	0.036(4)	0.017(3)	0.031(4)	-0.003(3)	0.005(3)	0.002(3)
C(32)	0.032(4)	0.043(4)	0.028(4)	-0.001(3)	0.013(3)	-0.011(3)

*The exponent takes the form: $-2\pi^2\Sigma\Sigma U_{ij}h_ih_j\mathbf{a}_i^*\mathbf{a}_j^*$

Table S2-5. Coordinates and isotropic temperature factors* (Å^2) for H atoms.

Atoms	*x*	*y*	*z*	$U_{eq.}$
H(2)	0.4747	0.1783	0.4072	0.029
H(5)	0.8398	-0.2284	0.2950	0.039
H(6)	1.0451	-0.5054	0.3160	0.049
H(7)	1.2007	-0.5051	0.3807	0.046
H(8)	1.1706	-0.2247	0.4224	0.036
H(10)	0.9117	0.4408	0.4525	0.029
H(11A)	0.5621	0.2453	0.4845	0.031
H(11B)	0.7896	0.1799	0.4961	0.031
H(12A)	0.8554	0.5040	0.5255	0.039
H(12B)	0.6677	0.4030	0.5464	0.039
H(13A)	0.4363	0.6024	0.5067	0.046
H(13B)	0.5944	0.7507	0.5290	0.046
H(14A)	0.5494	0.8407	0.4594	0.042
H(14B)	0.7795	0.7824	0.4700	0.042
H(15A)	0.6715	0.6197	0.4090	0.036
H(15B)	0.4897	0.5135	0.4310	0.036
H(16A)	0.2764	0.1171	0.3461	0.058
H(16B)	0.3871	-0.0538	0.3216	0.058
H(16C)	0.3873	0.1742	0.3062	0.058
H(18)	0.5282	0.6104	0.1565	0.029
H(21)	0.9285	0.1881	0.2667	0.035
H(22)	1.1255	-0.0851	0.2444	0.039
H(23)	1.2636	-0.0779	0.1808	0.038
H(24)	1.2182	0.2113	0.1398	0.032
H(26)	0.9440	0.8709	0.1113	0.027
H(27A)	0.5841	0.6729	0.0790	0.030
H(27B)	0.8080	0.6114	0.0676	0.030
H(28A)	0.8572	0.9360	0.0381	0.039
H(28B)	0.6606	0.8348	0.0173	0.039
H(29A)	0.4473	1.0333	0.0569	0.047
H(29B)	0.5959	1.1812	0.0345	0.047
H(30A)	0.5814	1.2725	0.1042	0.041
H(30B)	0.8070	1.2137	0.0936	0.041
H(31A)	0.7249	1.0489	0.1547	0.034
H(31B)	0.5328	0.9442	0.1328	0.034
H(32A)	0.3570	0.5625	0.2193	0.051
H(32B)	0.4942	0.5839	0.2595	0.051
H(32C)	0.4657	0.3687	0.2386	0.051

*The exponent takes the form: $-8\pi^2 U \sin^2\theta/\lambda^2$

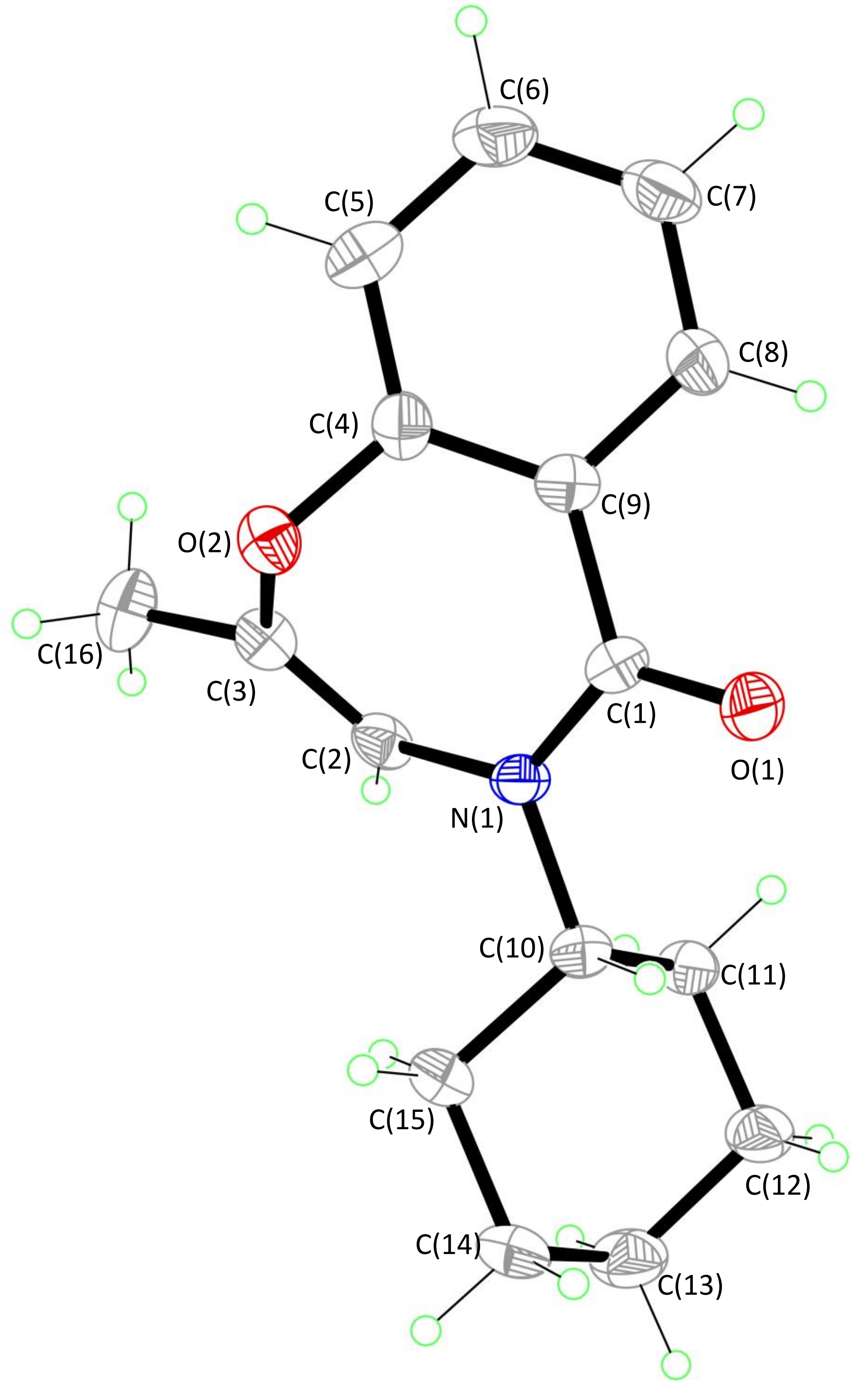

Molecule **I**

ORTEP drawing of $C_{16}H_{19}NO_2$ with 50% probability ellipsoids, showing the atomic numbering scheme.

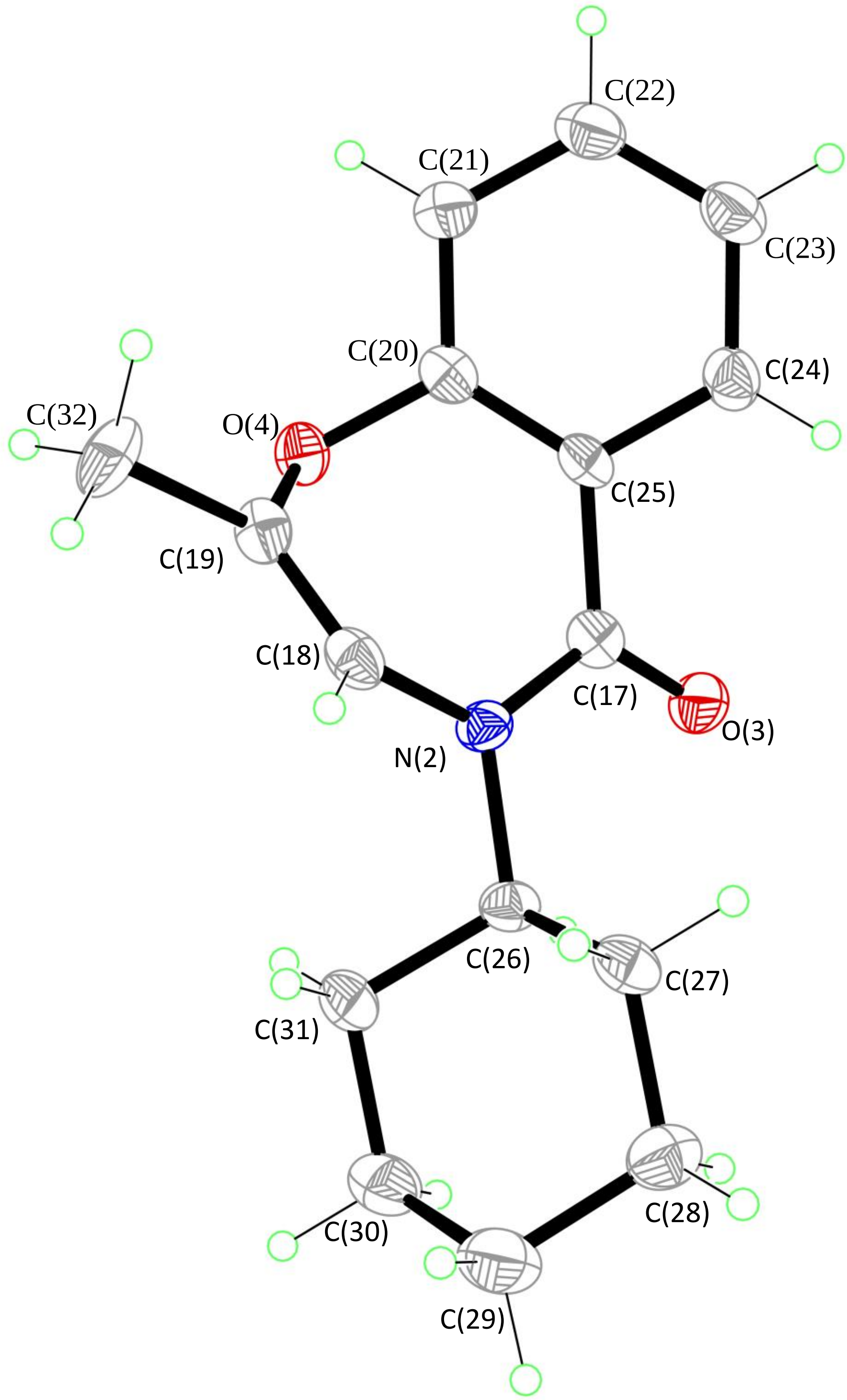

Molecule **II**

ORTEP drawing of $C_{16}H_{19}NO_2$ with 50% probability ellipsoids, showing the atomic numbering scheme.

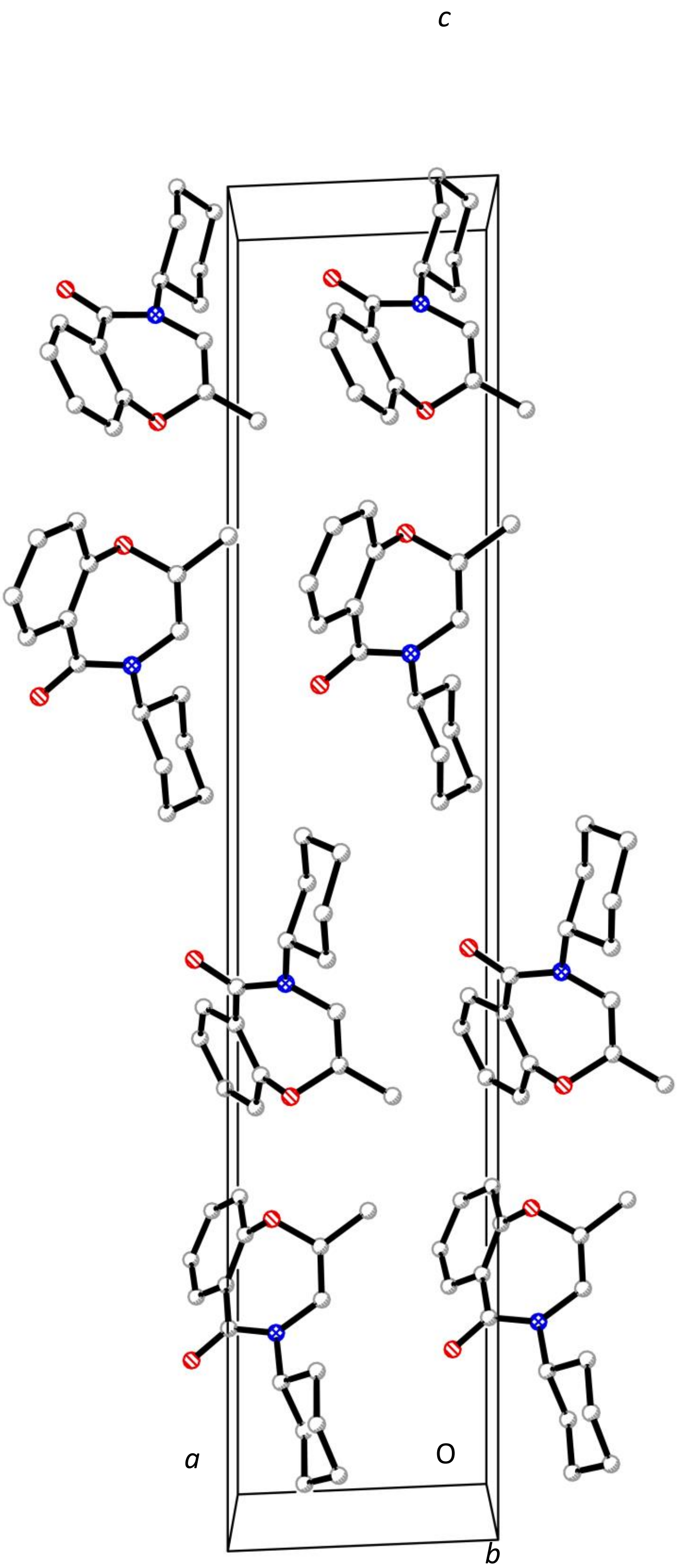

A packing view along the *b* direction

Crystal Structure of $C_{16}H_{19}NO_2$

The low temperature (173 ± 2°K) single-crystal X-ray experiments were performed on a SuperNova diffractometer with Cu K_α radiation. Unit cell was obtained and refined by 4943 reflections with 5.3° < θ < 71.2°. No decay was observed in data collection. Raw intensities were corrected for Lorentz and polarization effects, and for absorption by empirical method. Direct phase determination yielded the positions of all non-hydrogen atoms. All non-hydrogen atoms were subjected to anisotropic refinement. All hydrogen atoms were generated geometrically with C-H bonds of 0.93–0.98 Å according to criteria described in the SHELXTL manual (SHELXTL. Structure Determination Programs, Version 5.10, Bruker AXS Inc., Madison, WI, USA, 1997). They were included in the refinement with $U_{iso}(H) = 1.2U_{eq}$ of their parent atoms. The final full-matric least-square refinement on F^2 converged with $R1 = 0.0395$ and $wR2 = 0.0815$ for 2237 observed reflections [$I \geq 2\sigma(I)$]. The final difference electron density map shows no features. Details of crystal parameters, data collection and structure refinement are given in Table S3-1.

Data collection was controlled by CrysAlisPro, Agilent Technologies, Version 1.171.36.32 (Oxford Diffraction Ltd., Abingdon, Oxfordshire, OX, UK, 2013). Computations were performed using the SHELXTL NT ver. 5.10 program package (Bruker AXS Inc., Madison, WI, USA) on an IBM PC 586 computer. Analytic expressions of atomic scattering factors were employed, and anomalous dispersion corrections were incorporated [2,3]. Crystal drawings were produced with XP (SHELXTL. Structure Determination Programs, Version 5.10, Bruker AXS Inc., Madison, WI, USA).

Table S3-1. Details of Data Collection, Processing and Structure Refinement.

Sample code	**3g**
Molecular formula	$C_{16}H_{19}NO_2$
Molecular weight	257.32
Color and habit	colorless block
Crystal size	0.20 × 0.25 × 0.30 mm
Crystal system	monoclinic
Space group	$P2_1/c$ (No. 14)
Unit cell parameters	a = 12.2516(4) Å α = 90.00°
	b = 8.9803(2) Å β = 107.083(3)°
	c = 12.5810(4) Å γ = 90.00°
	V = 1323.13(7) Å^3 Z = 4 F(000) = 552
Density (calcd)	1.292 g/cm^3
Diffractometer	SuperNova, Dual, Cu at home/near, AtlasS2
Radiation	Cu K$_\alpha$, λ = 1.54178 Å
Temperature	173±2K
Scan type	ω-scan
Data collection range	-14 < h < 12, -10 < k < 8, -15 < l < 14; θ_{max} = 71.5°
Reflections measured	Total: 4794 Unique (n): 2495 Observed [I ≥ 2σ(I)]: 2237
Absorption coefficient	0.675 mm^{-1}
Minimum and maximum transmission	0.834, 1.000
No. of variables, p	172
Weighting scheme	$w = \dfrac{1}{\sigma^2(F_o^2) + (0.001P)^2 + 1.2P}$ $P = (F_o^2 + 2F_c^2)/3$
$R1 = \dfrac{\Sigma\|\|F_o\|-\|F_c\|\|}{\Sigma\|F_o\|}$ (for all reflections)	0.0440 0.0395 (for observed data)
$wR2 = \sqrt{\dfrac{\Sigma[w(F_o^2 - F_c^2)^2]}{\Sigma w(F_o^2)^2}}$ (for all reflections)	0.0839 0.0815 (for observed data)
Goof = S = $\sqrt{\dfrac{\Sigma[w(F_o^2 - F_c^2)^2]}{n - p}}$	1.017
Largest and mean Δ/σ	0.000, 0.000
Residual extrema in final difference map	-0.246 to 0.236 e Å^{-3}

Table S3-2. Atomic coordinates and equivalent isotropic temperature factors* (Å^2).

Atoms	*x*	*y*	*z*	$U_{eq.}$
O(1)	0.75990(9)	0.14968(13)	0.02237(9)	0.0269(3)
O(2)	0.60713(9)	0.02934(12)	0.25157(9)	0.0251(3)
N(1)	0.74999(11)	0.16686(14)	0.20035(10)	0.0209(3)
C(1)	0.72761(12)	0.09955(17)	0.09910(12)	0.0209(3)
C(2)	0.71947(13)	0.09304(17)	0.29122(12)	0.0219(3)
C(3)	0.59691(12)	-0.06619(17)	0.16334(13)	0.0219(3)
C(4)	0.52465(13)	-0.18757(18)	0.15087(14)	0.0268(4)
C(5)	0.51384(14)	-0.28301(19)	0.06197(14)	0.0292(4)
C(6)	0.57451(14)	-0.25791(19)	-0.01406(1)	0.0297(4)
C(7)	0.64518(14)	-0.13489(18)	-0.00147(1)	0.0262(3)
C(8)	0.65738(12)	-0.03768(17)	0.08730(12)	0.0212(3)
C(9)	0.81198(13)	0.30915(17)	0.22123(12)	0.0215(3)
C(10)	0.73363(14)	0.43686(18)	0.23107(15)	0.0283(4)
C(11)	0.79679(15)	0.58592(18)	0.24525(15)	0.0309(4)
C(12)	0.90569(14)	0.58182(19)	0.34255(14)	0.0294(4)
C(13)	0.98185(14)	0.45271(19)	0.33097(14)	0.0291(4)
C(14)	0.91822(13)	0.30461(18)	0.32195(13)	0.0249(3)
C(15)	0.80351(13)	-0.02475(17)	0.34711(13)	0.0234(3)
C(16)	0.84976(19)	-0.0317(2)	0.45467(15)	0.0448(5)

*$U_{eq.}$ defined as one third of the trace of the orthogonalized **U** tensor.

Table S3-3. Bond lengths (Å) and bond angles (°).

O(1)-C(1)	1.2310(18)	C(5)-C(6)	1.391(2)
O(2)-C(3)	1.3792(18)	C(6)-C(7)	1.384(2)
O(2)-C(2)	1.4377(18)	C(7)-C(8)	1.391(2)
N(1)-C(1)	1.3630(19)	C(9)-C(10)	1.524(2)
N(1)-C(2)	1.4620(19)	C(9)-C(14)	1.527(2)
N(1)-C(9)	1.4702(19)	C(10)-C(11)	1.530(2)
C(1)-C(8)	1.485(2)	C(11)-C(12)	1.523(2)
C(2)-C(15)	1.499(2)	C(12)-C(13)	1.522(2)
C(3)-C(4)	1.384(2)	C(13)-C(14)	1.529(2)
C(3)-C(8)	1.395(2)	C(15)-C(16)	1.306(2)
C(4)-C(5)	1.384(2)		
C(3)-O(2)-C(2)	112.21(11)	C(7)-C(6)-C(5)	119.56(15)
C(1)-N(1)-C(2)	120.15(12)	C(6)-C(7)-C(8)	120.61(15)
C(1)-N(1)-C(9)	120.26(12)	C(7)-C(8)-C(3)	118.82(14)
C(2)-N(1)-C(9)	119.45(12)	C(7)-C(8)-C(1)	121.58(14)
O(1)-C(1)-N(1)	123.22(14)	C(3)-C(8)-C(1)	119.51(14)
O(1)-C(1)-C(8)	122.36(14)	N(1)-C(9)-C(10)	111.34(12)
N(1)-C(1)-C(8)	114.39(13)	N(1)-C(9)-C(14)	113.25(12)
O(2)-C(2)-N(1)	110.35(12)	C(10)-C(9)-C(14)	110.88(13)
O(2)-C(2)-C(15)	109.73(12)	C(9)-C(10)-C(11)	111.03(13)
N(1)-C(2)-C(15)	112.86(12)	C(12)-C(11)-C(10)	111.48(14)
O(2)-C(3)-C(4)	119.02(14)	C(13)-C(12)-C(11)	111.00(14)
O(2)-C(3)-C(8)	119.79(13)	C(12)-C(13)-C(14)	110.87(13)
C(4)-C(3)-C(8)	121.18(15)	C(9)-C(14)-C(13)	109.28(13)
C(3)-C(4)-C(5)	119.05(15)	C(16)-C(15)-C(2)	123.29(16)
C(4)-C(5)-C(6)	120.76(15)		

Table S3-4. Anisotropic thermal parameters* (Å^2).

Atoms	U_{11}	U_{22}	U_{33}	U_{23}	U_{13}	U_{12}
O(1)	0.0328(6)	0.0274(6)	0.0221(5)	0.0002(5)	0.0106(5)	-0.0053(5)
O(2)	0.0240(5)	0.0240(6)	0.0305(6)	-0.0050(5)	0.0131(5)	-0.0030(5)
N(1)	0.0267(7)	0.0158(6)	0.0212(6)	0.0000(5)	0.0084(5)	-0.0025(5)
C(1)	0.0213(7)	0.0184(8)	0.0219(7)	0.0008(6)	0.0047(6)	0.0033(6)
C(2)	0.0268(8)	0.0180(8)	0.0227(7)	-0.0027(6)	0.0100(6)	-0.0024(6)
C(3)	0.0198(7)	0.0197(8)	0.0250(8)	-0.0010(6)	0.0046(6)	0.0024(6)
C(4)	0.0214(8)	0.0262(9)	0.0328(9)	0.0021(7)	0.0080(7)	-0.0013(7)
C(5)	0.0239(8)	0.0224(8)	0.0368(9)	-0.0008(7)	0.0020(7)	-0.0048(7)
C(6)	0.0345(9)	0.0231(9)	0.0273(8)	-0.0055(7)	0.0023(7)	-0.0017(7)
C(7)	0.0294(8)	0.0242(8)	0.0245(8)	-0.0010(7)	0.0073(7)	0.0005(7)
C(8)	0.0204(7)	0.0181(8)	0.0234(7)	0.0005(6)	0.0040(6)	0.0009(6)
C(9)	0.0271(8)	0.0162(7)	0.0217(7)	0.0001(6)	0.0076(6)	-0.0029(6)
C(10)	0.0244(8)	0.0188(8)	0.0365(9)	-0.0017(7)	0.0007(7)	0.0008(6)
C(11)	0.0345(9)	0.0172(8)	0.0386(9)	-0.0017(7)	0.0071(8)	0.0006(7)
C(12)	0.0336(9)	0.0232(8)	0.0314(9)	-0.0067(7)	0.0098(7)	-0.0085(7)
C(13)	0.0239(8)	0.0300(9)	0.0317(9)	0.0000(7)	0.0057(7)	-0.0054(7)
C(14)	0.0223(8)	0.0218(8)	0.0298(8)	0.0022(7)	0.0065(6)	0.0019(6)
C(15)	0.0282(8)	0.0175(8)	0.0257(8)	0.0000(6)	0.0097(6)	-0.0034(6)
C(16)	0.0679(14)	0.0350(11)	0.0280(9)	0.0043(8)	0.0088(9)	0.0127(10)

*The exponent takes the form: $-2\pi^2\Sigma\Sigma U_{ij}h_ih_j\mathbf{a}_i^*\mathbf{a}_j^*$

Table S3-5. Coordinates and isotropic temperature factors* (Å^2) for H atoms.

Atoms	*x*	*y*	*z*	$U_{eq.}$
H(2)	0.7181	0.1687	0.3469	0.026
H(4)	0.4839	-0.2048	0.2015	0.032
H(5)	0.4655	-0.3649	0.0530	0.035
H(6)	0.5676	-0.3234	-0.0730	0.036
H(7)	0.6849	-0.1171	-0.0529	0.031
H(9)	0.8379	0.3298	0.1561	0.026
H(10A)	0.7054	0.4198	0.2945	0.034
H(10B)	0.6685	0.4403	0.1648	0.034
H(11A)	0.8158	0.6093	0.1776	0.037
H(11B)	0.7471	0.6639	0.2576	0.037
H(12A)	0.9468	0.6747	0.3455	0.035
H(12B)	0.8861	0.5716	0.4115	0.035
H(13A)	1.0492	0.4503	0.3951	0.035
H(13B)	1.0065	0.4672	0.2652	0.035
H(14A)	0.9676	0.2237	0.3139	0.030
H(14B)	0.8963	0.2875	0.3890	0.030
H(15)	0.8237	-0.0964	0.3030	0.028
H(16A)	0.8309	0.0387	0.5006	0.054
H(16B)	0.9015	-0.1071	0.4853	0.054

*The exponent takes the form: $-8\pi^2 U \sin^2\theta/\lambda^2$

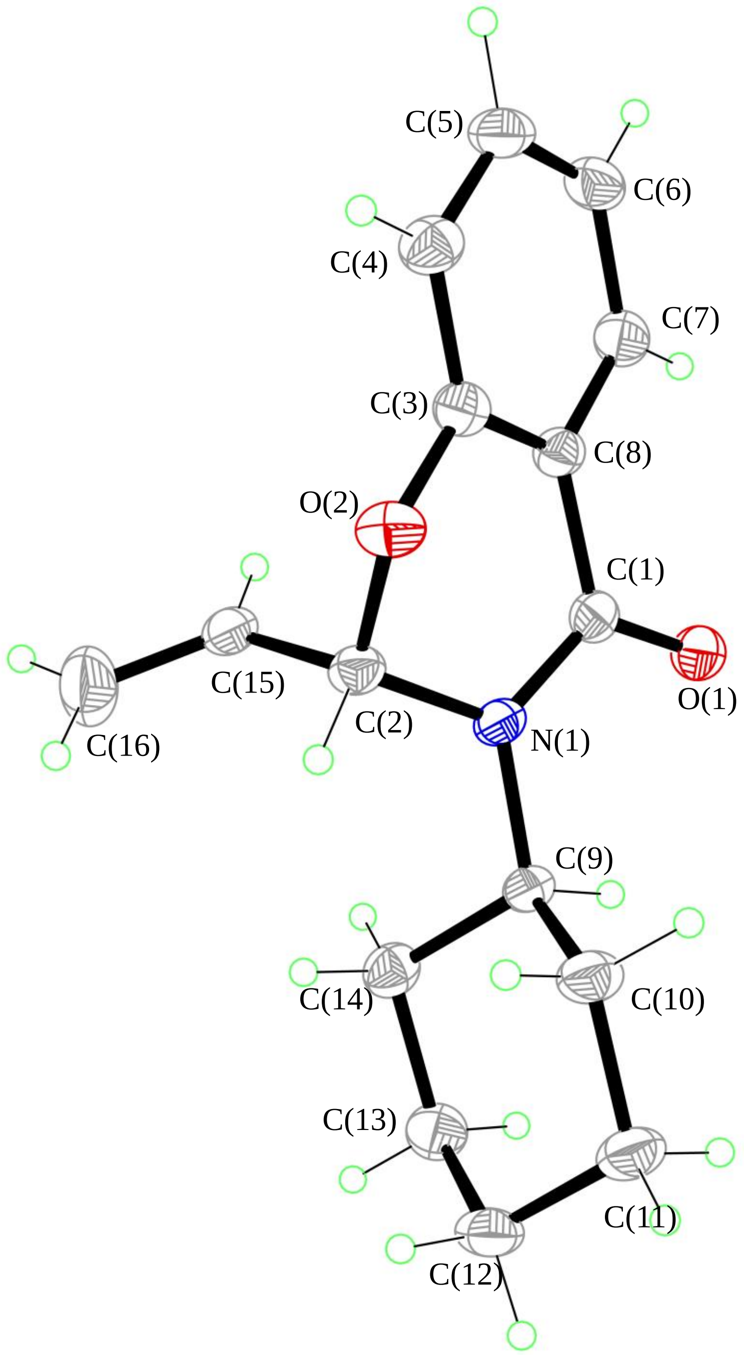

ORTEP drawing of $C_{16}H_{19}NO_2$ with 50% probability ellipsoids, showing the atomic numbering scheme.

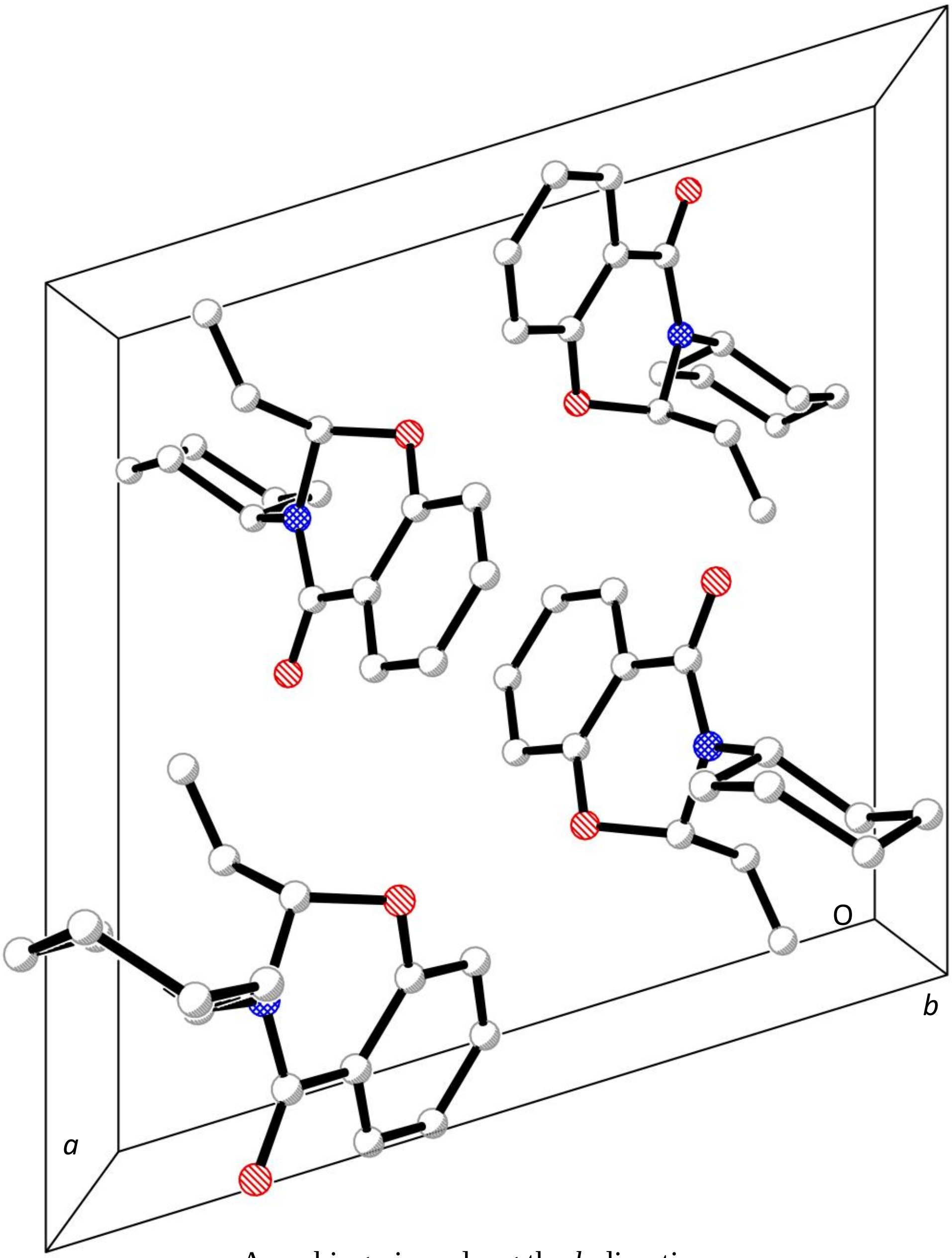

A packing view along the *b* direction

6. The results from the reactions of 1a with propargyl alcohol in either KOD/D_2O/DMSO or KOD/D_2O/MeCN

The reaction of **1a** with propargyl alcohol in either KOD/D_2O/DMSO or KOD/D_2O/MeCN have been performed, and the products are called as **2a-D** and **3a-D**.

We have compared the GC-MS results of **2a-D** and **3a-D** with **2a** and **3a**, respectively. **2a-D** and **3a-D** have been isolated, and their ^{1}H-NMR spectra have been compared with ones of **2a** and **3a**.

On the basis of the comparison, it seems to observe the D-incorporated into **2a-D** and **3a-D** in low percentages, but these observations cannot confirm whether D is really incorporated or not.

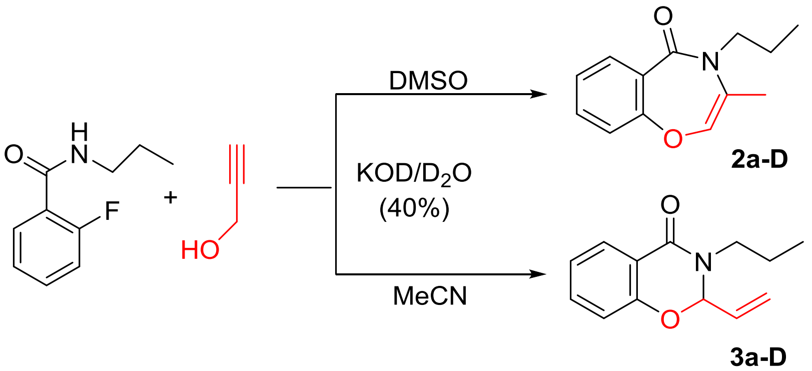

Possible D incorporated position in **2a** Possible D incorporated position in **3a**

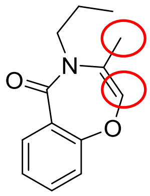

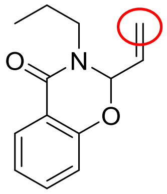

GC-MS results

According to GC-MS charts, the peak of 218 (from one D isotope) in **2a-D** sample seems to be higher than **2a**. It may imply that one D is incorporated into **2a-D**.

GC-MS of **2a**:

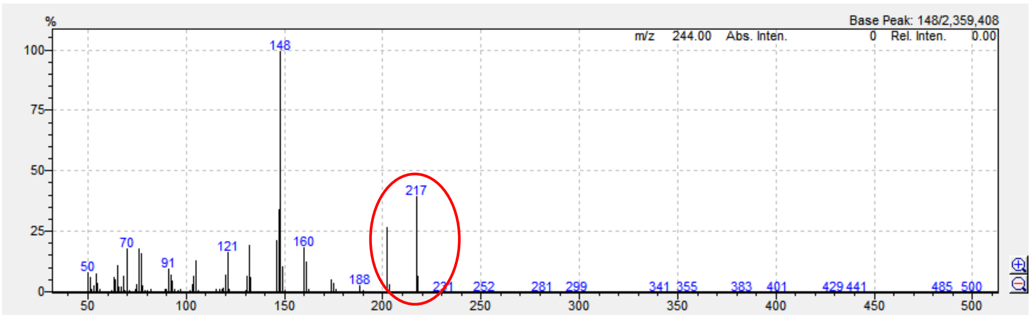

GC-MS of 2a-D

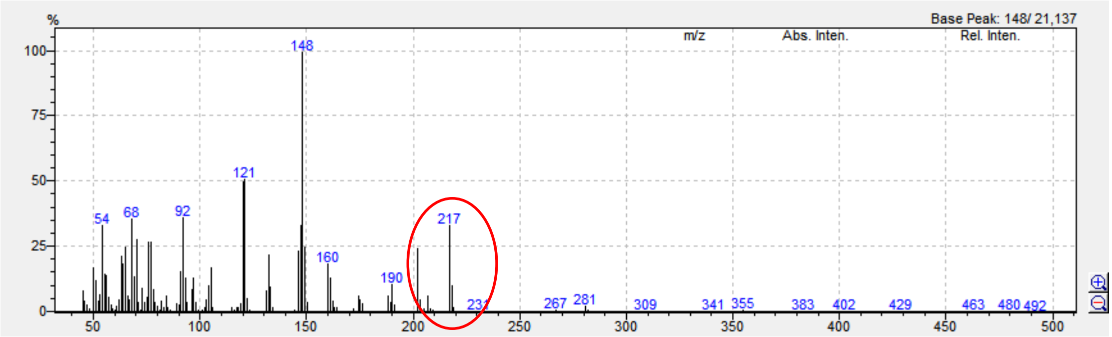

The similar phenomenon can be also observed, when we compare the GC-MS spectra of **3a** with **3a-D**.

GC-MS of **3a**

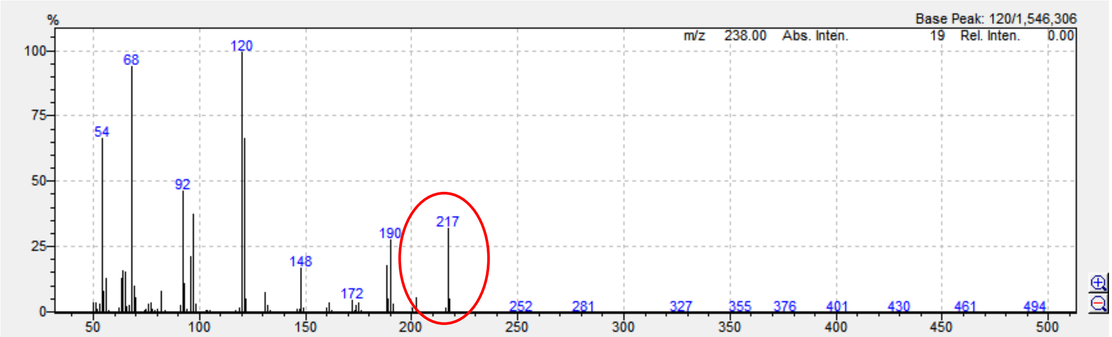

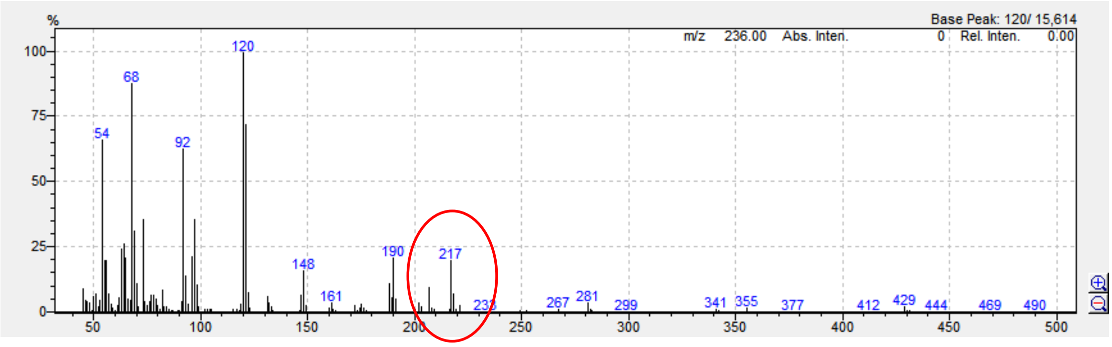

^{1}H NMR results

Slight splitting peaks seem to be observed at 5.43 ppm and 1.93 ppm. We assume that it is from D-incorporated in **2a-D** into vinyl and methyl group.

^{1}H NMR of **2a**

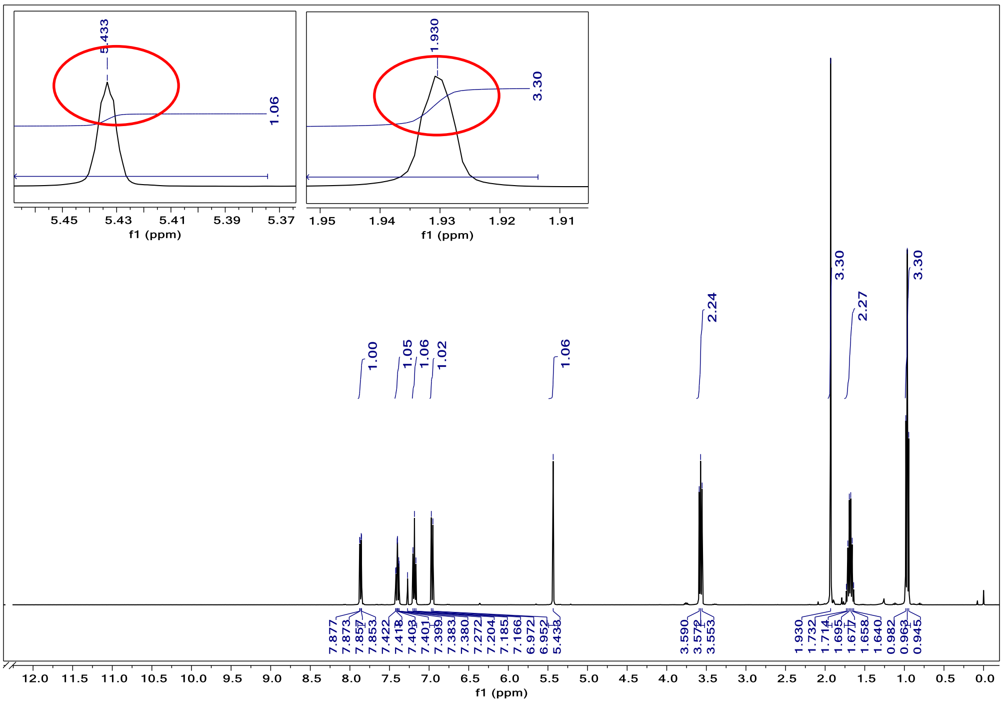

^{1}H NMR of **2a-D**

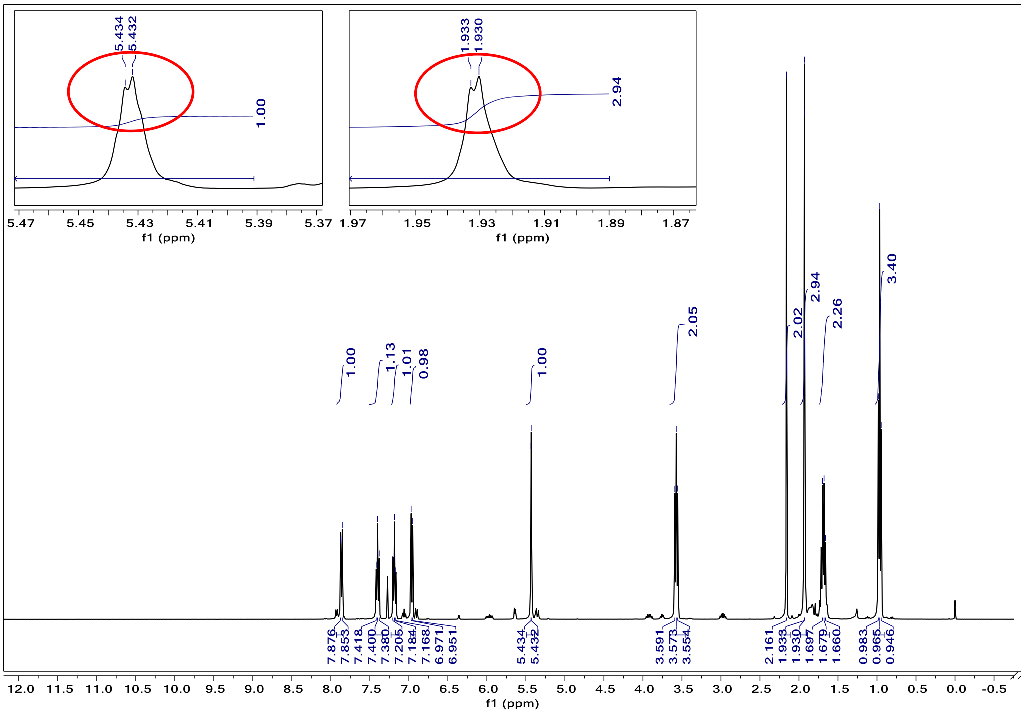

Also, slight splitting peaks seem to be observed at 5.35 ppm, and we assume that it is from D-incorporated in **3a-D** into vinyl group.

^{1}H NMR of **3a**

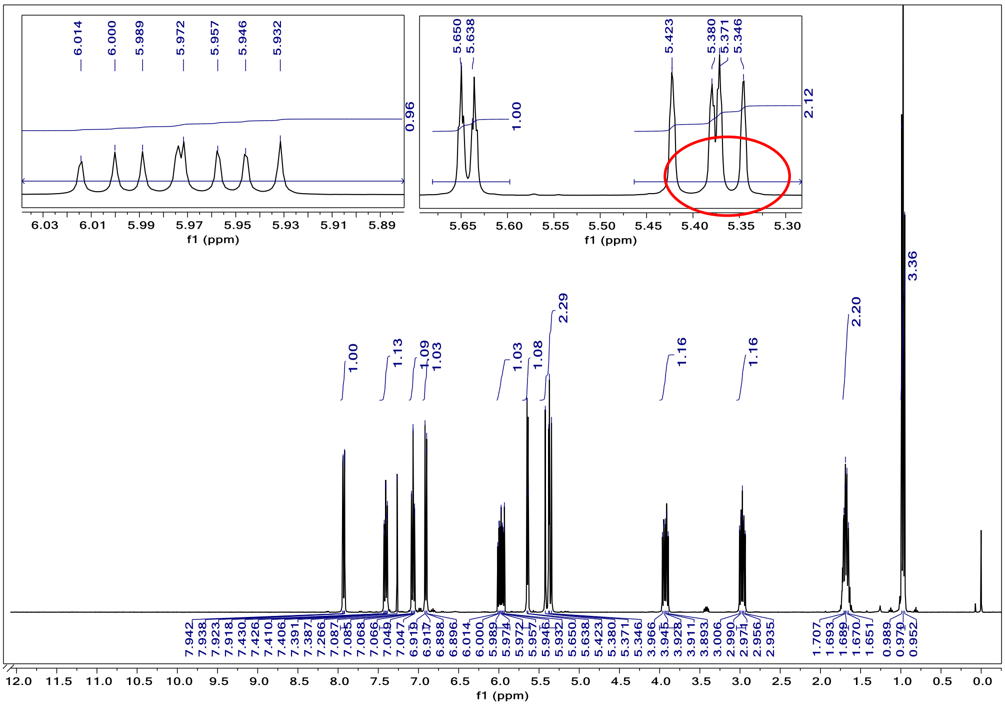

^{1}H NMR of **3a-D**

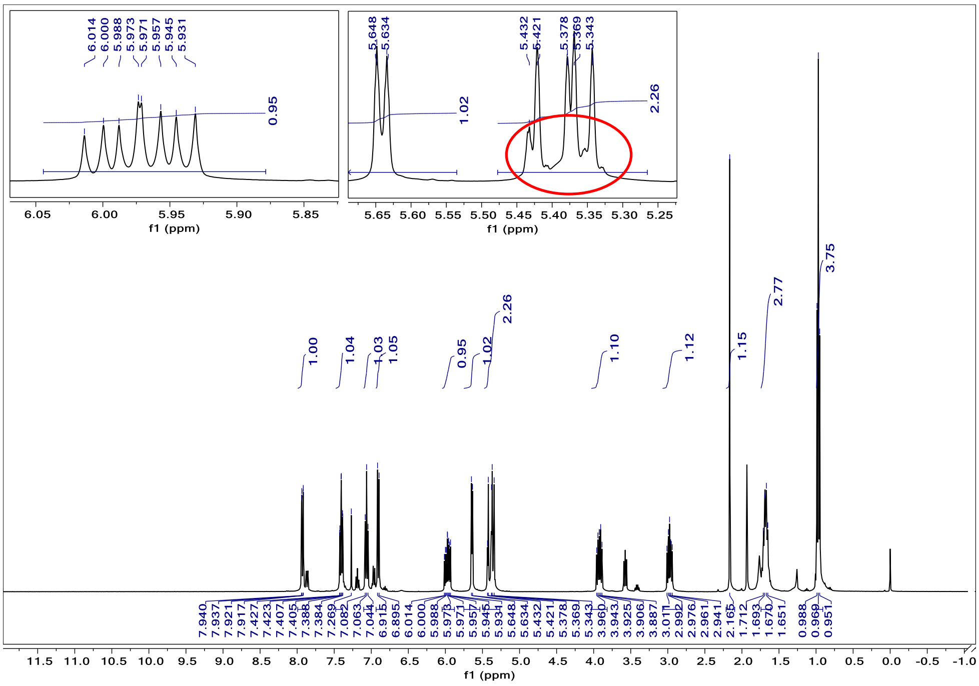

References

1. Seth, K.; Nautiyal, M.; Purohit, P.; Parikh, N.; Chakraborti, A.K. Palladium catalyzed Csp^2–H activation for direct aryl hydroxylation: The unprecedented role of 1,4-dioxane as a source of hydroxyl radicals. *Chem. Commun.* **2015**, *51*, 191–194.
2. Creagh, D.C. Tables 4.2.6.8. In *International Tables for Crystallography*; Wilson, A.J.C., Prince, E., Eds.; Kluwer Academic Publisher: Dordrecht, the Netherlands, 1999; Volume C, pp. 255–257.
3. Maslen, E.N.; Fox, A.G.; O'Keefe, M.A. Tables 6.1.1.4. In *International Tables for Crystallography*; Wilson, A.J.C., Prince, E., Eds.; Kluwer Academic Publisher: Dordrecht, the Netherlands, 1999; Volume C, pp. 572–574.
